# Multi-Locus Genome Wide Association Mapping for Yield and Its Contributing Traits in Hexaploid Wheat under Different Water Regimes

**DOI:** 10.1038/s41598-019-55520-0

**Published:** 2019-12-20

**Authors:** Vijay Gahlaut, Vandana Jaiswal, Sukhwinder Singh, H. S. Balyan, P. K. Gupta

**Affiliations:** 1 Department of Genetics and Plant Breeding, Ch. Charan Singh University, Meerut, India; 2 Department of Plant Molecular Biology, University of Delhi, South Campus, New Delhi, India; 3 School of Life Sciences, Jawaharlal Nehru University, New Delhi, India; 4 CIMMYT, Mexico, DF Mexico

**Keywords:** Quantitative trait, Agricultural genetics, Plant breeding

## Abstract

Multi-locus genome wide association study was undertaken using a set of 320 diverse spring wheat accessions, which were each genotyped for 9,626 SNPs. The association panel was grown in replicated trials in four environments [two each in irrigated (IR) and rainfed (RF) environments], and phenotypic data were recorded for five traits including days to heading, days to maturity, plant height, thousand grain weight and grain yield. Forty-six significant marker-trait associations (MTAs) were identified for five traits. These included 20 MTAs in IR and 19 MTAs in RF environments; seven additional MTAs were common to both the environments. Five of these MTAs were co-localized with previously known QTL/MTAs and the remaining MTAs were novel and add to the existing knowledge. Three desirable haplotypes for agronomic traits, one for improvement in RF environment and two for improvement in IR environment were identified. Eighteen (18) promising candidate genes (CGs) involved in seven different biological activities were also identified. The expression profiles of four (Trehalose-6-Phosphate, APETALA2/Ethylene-responsive factor, DNA-binding One Zinc Finger and Gibberellin-dioxygenases) of the 18 genes showed that they were induced by drought stress in the wheat seedlings. The MTAs, haplotypes and CG-based markers may be used in marker-assisted breeding for drought tolerance in wheat.

## Introduction

Wheat (*Triticum aestivum* L.) is an important staple crop that is widely grown in a range of environments and provides about 20% of the daily required protein and calories for 4.5 billion people world-over^1^. In recent years, the annual growth in wheat production has declined from 3% in the past to <0.7% in recent years, which is a cause of concern^2^. It has also been recognized that currently there is limited water supply for irrigation in 70% of the land area under wheat cultivation; this area with limited water supply may increase in future and would thus become the major cause of limiting global wheat production^3–5^. Therefore, development of drought-resilient and water-use efficient cultivars is a thrust area of research for wheat breeders world-over to meet the future demands of wheat production.

The development of drought-resilient and high yielding wheat cultivars continues to be a challenge, also partly due to low heritability and large “genotype × environment” interactions associated with yield as a trait, particularly under drought^6^. Therefore, newer and high throughput (HT) genomics/phenomics approaches are currently being utilized to study the genetics of yield and its contributing traits under water stress. Hopefully, the information generated from these newer approaches will prove useful for further improvement in wheat productivity not only under drought, but also under limited water availability^6,7^.

Genetic basis of drought tolerance has been studied in several crops. In *Arabidopsis* also, SWI2/SNF2 remodeling was found to maintain equilibrium between plant growth and drought stress tolerance^8^. Transcription factors like NAC, DREB, WRKY, MYB, bZIP have also been reported to play a major role in drought stress tolerance^9^. Similarly, CDPK7, CIPK03 and CIPK12 genes in rice were reported to be involved in signal transduction and protein kinase activity to provide tolerance against drought stress^10,11^. Further, in brassica, the gene *BnPIP1* involved in ion and osmotic homeostasis^12^ and the gene *BnPtdIns-PLC2* was found to provide tolerance against drought though phospholipid metabolism^10^. A number of QTLs have also been reported for drought stress tolerance in several crops including rice^13^, barley^14^ and sorghum^15^.

A number of studies involving QTL analysis for different agronomic traits including grain yield and its component traits under favourable and stress (e.g. rainfed, drought, heat) environments were conducted in the recent past. These involved interval mapping and genome wide association studies (GWAS) in hexaploid and durum wheat^16^. Interval mapping also allowed identification of stable QTLs, which showed expression across multiple environments^17–21^. Similarly, GWAS identified marker-trait associations (MTAs) for yield and its component traits^22–30^. However, limited efforts have been made to study the genetics of grain yield and its components under drought conditions experienced by wheat crop in Indian sub-continent. Also, only few studies involving multi-locus and multi-trait GWA analysis have been conducted^27,31–33^; more such studies need to be undertaken to overcome the limitations of routine single locus single trait GWAS.

The present study was conducted to identify MTAs involving yield and related traits using LD-based multi-locus GWA mapping using GBS (genotyping by sequencing)-derived SNP markers in a large association panel consisting of 320 spring wheat accessions. The Wheat association mapping panel grown under different water regimes [i.e. irrigated (IR) and rainfed (RF] during growing seasons (2011–12 and 2012–13) and phenotypic data were collected for five different traits. Post-GWAS haplotype analysis was also undertaken to study the joint effect of associated SNPs and to identify desirable genotypes. Candidate genes (CGs) underlying some of the MTAs were identified and annotated through expression analysis. The results from the present study should prove useful for genetic improvement of yield and yield contributing traits via marker-assisted breeding to enhance productivity under limited water availability.

## Results

### Phenotypic variability in the wheat association mapping (WAM) panel

**Figure 1:**
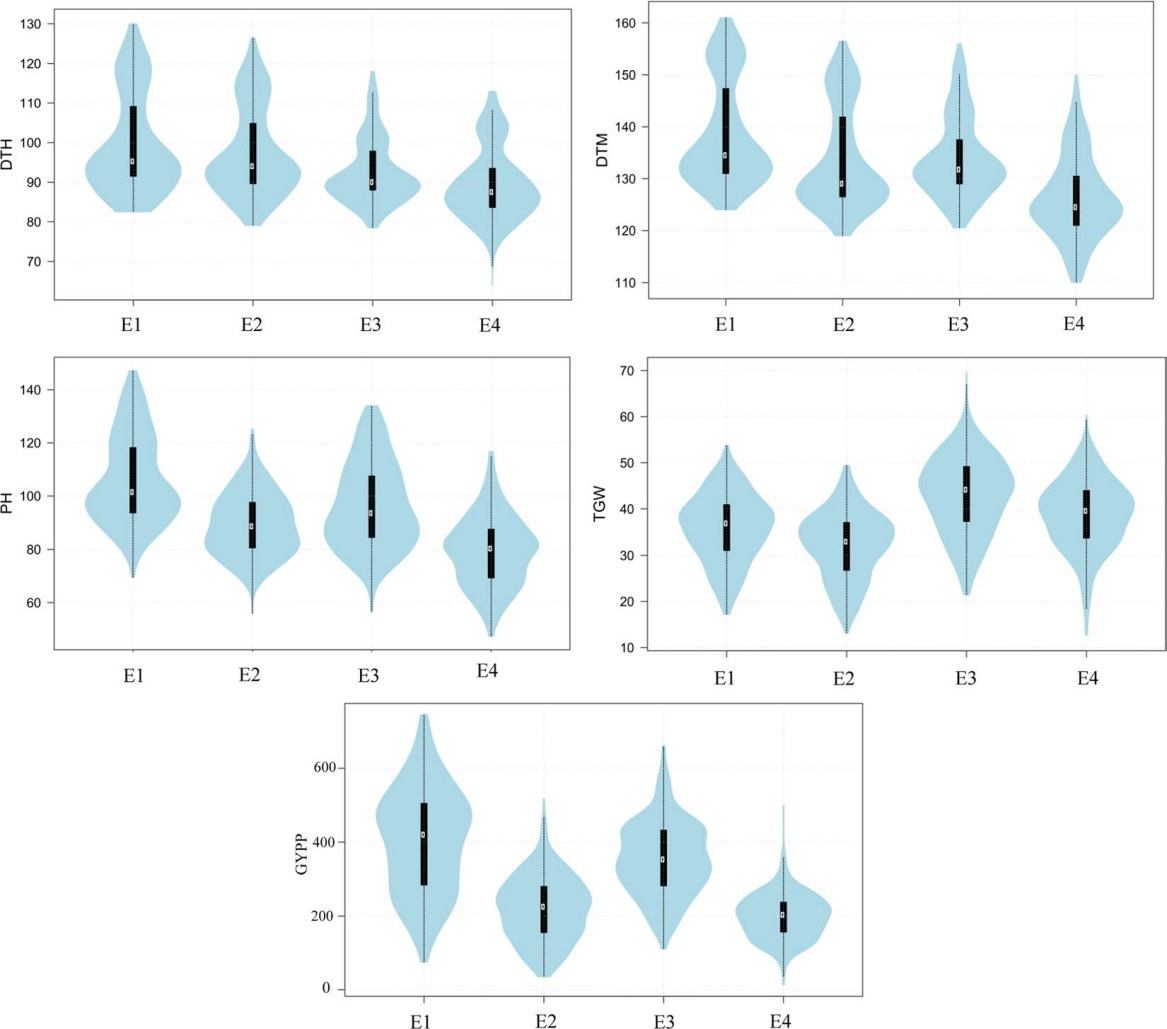
Violin plots showing the distribution of values for five grain yield related traits (DTH, DTM, PH, TGW, GYPP) in four environments (E1-E4).

A wide range of variability for each of the five traits was available in the WAM panel in all the four environments including both locations (Meerut and Powerkheda) each with two environmental conditions (IR and RF); the data is summarised in Supplementary Table S1. Coefficient of variation (CV) ranged from 5.6% [for days to maturity (DTM) in E3] to 39.7% [for grain yield per plot (GYPP) in E2]. Skewness and kurtosis for each of the five traits under all the four environmental conditions (E1-E4) were within the acceptable range of normal distribution (±2). The violin plots showing distribution of all the five traits in different environments are presented in Fig. 1. Continuous variation was observed for all the five traits at both the locations. Taken together, the extent of available variability for the different traits suggested suitability of WAM panel for conducting GWAS.

Pearson’s correlation coefficient analysis revealed that all the five traits were significantly correlated among themselves in IR as well as RF environments (Supplementary Table S2). Grain yield (GY) and thousand grain weight (TGW) were negatively correlated with days to heading (DTH), DTM and plant height (PH); however, positive correlation was observed between GY and TGW. Similarly, DTM, DTH and PH were positively correlated with each other.

### Distribution of SNPs across genome

**Figure 2:**
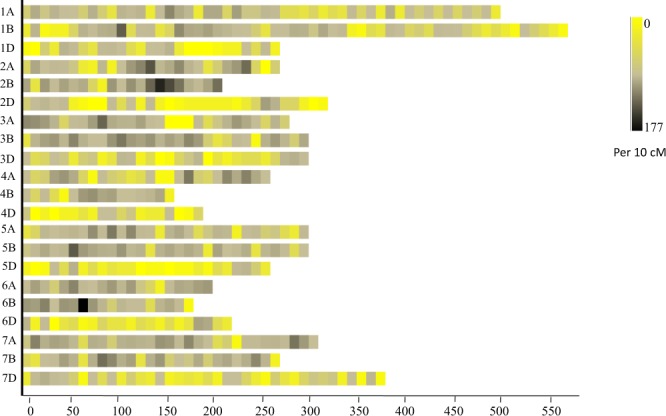
Single nucleotide polymorphism (SNP) density on the 21 wheat chromosomes. The x-axis shows length of chromosomes in cM. The different colors depict SNP density (the number of SNPs per window i.e. 10 cM).

A set of 9627 mapped SNPs were utilized during the present study; these were distributed on all the 21 wheat chromosomes, spanning 5943.1 cM (Supplementary Table S3). Maximum number of 4501 SNPs (total length 1953.3 cM) were available in sub-genome B, followed by sub-genome A (3969 SNPs; 2084.2 cM), and sub-genome D (1157 SNPs; 1157 cM). For individual chromosome, number of SNPs ranged from 77 (4D) to 874 (2B). Heat map showing SNP density in all the 21 chromosomes is shown in Fig. 2. The maximum SNP density was observed on sub-genome B (25 SNPs/10 cM) followed by sub-genome A (20 SNPs/10 cM) and sub-genome D (7 SNPs/10 cM). The average SNP density per 10 cM on individual chromosomes ranged from 4 (on 4D) to 42 (on 2B). Whole genome average SNP density was 17 SNPs/10 cM (Supplementary Table S3).

### Genetic diversity, population structure and LD

Genetic diversity based on SNP genotypic data suggested that WAM panel is diverse (whole genome average gene diversity = 0.283). Maximum genetic diversity was observed for sub-genome B (0.33) followed by sub-genome A (0.30), and sub-genome D (0.22) (Supplementary Table S4). SNPs showed moderate to high polymorphism with average polymorphic information content (PIC) of 0.24. For individual sub-genomes, PIC ranged from 0.22 (sub-genome D) to 0.33 (sub-genome B). The number of sub-populations was three with G1 containing 57 genotypes, G2 containing 85 genotypes and G3 containing 15 genotypes; the remaining 65% (163 genotypes) were distributed in more than one sub-population and were therefore treated as admixture (Supplementary Fig. S1). LD analysis was conducted as a part of another study using the same set of data; LD decay distance ranged from 2 cM to 20 cM in different genomic regions with a genome-wide LD decay of 3 cM^27^.

### Marker–trait associations (MTAs)

Using Bonferroni correction (−log p value ≥ 6.0), 46 significant MTAs were identified for five traits (DTH, DTM, PH, TGW and GY) in four different environments; 39 (85%) MTAs were detected in individual environment only; the remaining 7 (15%) occurred in two or more environments. MTAs were distributed on 18 wheat chromosomes excluding chromosomes 1D, 3D, and 7D (Table 1; Supplementary Fig. S2). Q-Q plots showing appropriate model fitting for GWAS tests are shown in Supplementary Fig. S2. The details of the MTAs are given in Table 1 and a summary of the results is presented here. Number of MTAs for individual traits were 5 (PH), 6 (GYPP), 10 (TGW), 11 (DTM) and 14 (DTH). The effect of SNPs involved in individual MTAs ranged from −4.1 to 4.9 for DTH, −3.3 to 3.9 for DTM, −4.6 to 4.00 for PH, −2.8 to 2.1 for TGW and −29.3 to 32.3 for GYPP (Table 1).

**Table 1:** List of significant SNP markers (qualified Bonferroni criteria) associated with yield and its related traits, desirable allele and SNP effects.

Trait	Environment	SNP	Clone ID	Chromosome	Position (cM)	SNP alleles	Desirable allele	−log (p)	SNP effect
DTH	E1, E2	SNP_404	1103347	1A	247.886	G/T	G	8.5–8.1	3.7 to 4.7
	E1	SNP_647	1096003	1B	64.812	G/T	G	7.1	4.9
	E1, E2	SNP_5304	1003062	4B	60.121	T/G	G	8.3–6.7	−4.1 to −4.3
	E2	SNP_2860	1026541	2B	179.515	C/G	C	9.3	4.5
	E2	SNP_7937	1129498	6D	9.626	T/C	T	8.0	2
	E2	SNP_6361	1050557	5B	85.982	G/C	C	6.4	−2.7
	E2	SNP_3398	1063624	3A	75.969	G/T	G	6.1	3.4
	E3	SNP_1204	986158	1B	384.128	A/T	A	10.2	2.7
	E3	SNP_5593	1110448	4D	123.880	G/T	T	7.7	−4.0
	E3	SNP_5815	1133812	5A	96.128	C/G	C	6.6	1.8
	E3, E4	SNP_4482	1069547	3B	253.745	C/A	C	12.1–6.2	2.0 to 2.9
	E3	SNP_4509	1134753	3B	264.027	C/G	G	6.1	−1.9
	E4	SNP_6544	3023142	5B	171.943	G/C	C	7.6	−2.6
	E4	SNP_2075	1083327	2A	237.185	G/C	G	7.5	2.2
DTM	E1, E2, E4	SNP_404	1103347	1A	247.886	G/T	G	9.4–6.7	3.0 to 3.9
	E1, E2	SNP_5304	1003062	4B	60.121	T/G	G	6.4–6.1	−3.2 to −3.3
	E1, E2	SNP_3398	1063624	3A	75.969	G/T	G	6.2–6.1	3.6 to 3.7
	E2	SNP_1302	100001553	1B	520.423	T/C	C	7.3	−3.1
	E2	SNP_647	1096003	1B	64.812	G/T	G	7.0	3.8
	E3	SNP_5369	1270494	4B	76.346	T/C	C	8.1	−2.7
	E3	SNP_9088	992291	7B	114.753	C/T	C	7.7	1.4
	E3	SNP_2054	1125402	2A	231.786	C/G	G	7.0	−3.1
	E3	SNP_7797	1093068	6B	82.603	C/T	T	6.5	−1.7
	E3	SNP_7068	1073429	6A	88.944	C/T	C	6.1	2.2
	E4	SNP_2003	1021420	2A	224.605	G/A	A	8.7	−2.2
PH	E2	SNP_5988	1090844	5A	177.183	G/C	C	7.5	−3.0
	E3	SNP_5396	3028936	4B	80.804	C/T	C	6.5	4.0
	E3	SNP_7799	1080969	6B	83.710	C/G	C	6.1	3.8
	E4	SNP_4022	1127414	3B	94.818	A/G	G	7.6	−3.4
	E4	SNP_693	1113894	1B	91.804	T/G	G	6.8	−4.6
TGW	E1, E2	SNP_7068	1073429	6A	88.944	C/T	C	7.9–6.2	−2.1 to −2.8
	E1	SNP_7775	1026425	6B	75.404	G/C	G	6.0	−2.4
	E2	SNP_5008	1094628	4A	177.335	T/G	G	8.3	1.5
	E2	SNP_6346	1167608	5B	79.052	G/T	T	7.8	1.5
	E2	SNP_5294	985312	4B	57.883	G/A	G	7.7	−1.5
	E2	SNP_3294	3030495	3A	21.260	A/G	A	6.5	−1.7
	E2	SNP_3179	1228079	2D	289.675	T/C	T	6.4	−2.1
	E3	SNP_7797	1093068	6B	82.603	C/T	T	6.2	2.1
	E4	SNP_1829	977937	2A	150.126	G/A	G	6.9	−1.8
	E4	SNP_3449	1065193	3A	88.133	T/C	T	6.9	−1.8
GYPP	E1	SNP_5249	1126379	4A	250.448	T/A	T	7.0	−29.3
	E1	SNP_7747	985813	6B	69.052	C/A	C	6.9	−33.7
	E1	SNP_8465	1093542	7A	157.225	A/G	A	6.6	−29.3
	E3	SNP_6795	1091457	5D	100.955	A/G	G	8.2	32.3
	E3	SNP_4163	1202201	3B	117.678	G/A	A	7.1	26.9
	E3	SNP_2974	1217183	2B	203.502	A/G	A	6.1	−22.1

DTH, days to heading; DTM, days to maturity; PH, plant height; TGW, thousand grain weight; GYPP, grain yield per plot; E1 Meerut irrigated, E2, Meerut rainfed; E3, Powerkheda irrigated; E4, Powerkheda rainfed.

### Identification of superior haplotypes

**Figure 3:**
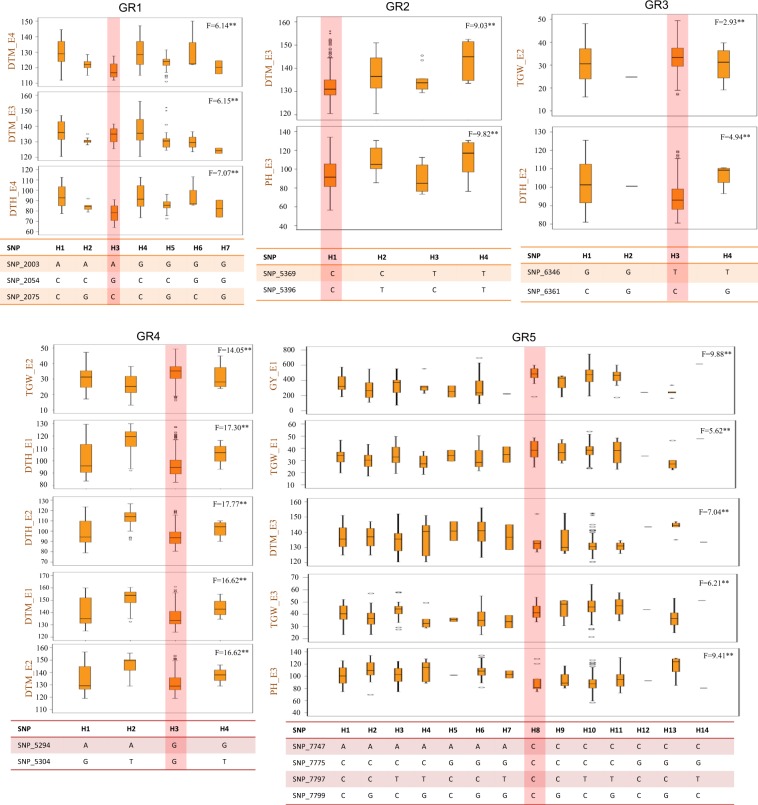
All possible haplotypes involving significantly associated SNPs in five genomic regions (GRs; GR1-GR5) for different agronomic traits. **Represents 0.01 level of significance level of F- test statistics.

In wheat, the genome-wide LD decay distance has been estimated to be 1.5–15 cM^26,27,34–37^. Therefore, in the present study also, we used an average decay of 10 cM and SNPs occurring within 10 cM were treated as haplotype blocks. Five genomic regions (GR1 to GR5), each with varying number of haplotypes for different traits involving four chromosomes (2A, 4B, 5B and 6B) were identified. Each haplotype was associated with two to four traits out of five traits considered during present study. The number of SNPs in individual haplotypes ranged from two to five. The number of observed haplotypes in individual GRs ranged from 4 (GR2, GR3 and GR4) to 14 (GR5). Univariate general linear model (GLM) suggested that there were significant differences for associated phenotypes among haplotypes (Fig. 3).

Out of five GRs, GR3 including four haplotypes (H1, H2, H3 and H4) was solely identified in RF environment and was associated with TGW and DTH. The GR2 (four haplotypes associated with PH and DTM) and GR5 (14 haplotypes associated with TGW, PH, DTM and GY) were uniquely identified in the IR environment. GR1 and GR4 were associated with DTH and DTM; and these were identified in both RF and IR environments. In each of the five GRs, most desirable haplotypes were also identified (highlighted in Fig. 3). For example, in each of GR1, GR2 and GR4, H3s were most suitable since each of these were associated with early flowering, maturity and reduced PH; H3 in GR4 was also associated with higher TGW. Similarly, in case of GR5, H8 was most desirable haplotype as it is associated with reduced PH, early maturity, higher TGW as well as GY.

### Joint effect of significant SNPs on associated phenotypes

**Figure 4:**
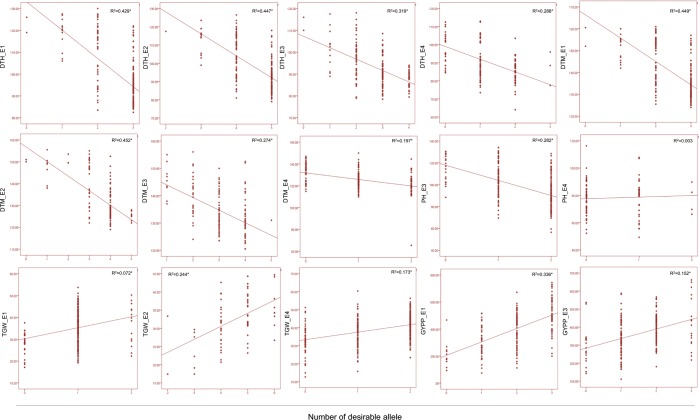
Linear regression analysis for phenotype (dependent variable) and number of desirable SNP alleles (independent variable). x-axis represents number of desirable SNP alleles and y-axis represents phenotypic values. R^2^ = regression coefficient; * represents 0.0001 level of significance.

For each of the five traits, more than one SNPs were found to be associated under one or more environmental conditions (except PH_E1, PH_E2, TGW_E3, GY_E2 and GY_E4). In order to determine the effect of number of desirable alleles of associated SNPs on phenotype, joint effect was estimated using linear regression. In case of DTH, DTM and PH, SNP alleles that lower the trait value were considered as desirable; however, in case of TGW and GY, SNP alleles that increases the trait value were considered as desirable. Interestingly, in all the cases, significant joint effects were observed except in case of PH_E4. For example, three SNPs (SNP_404, SNP_647 and SNP_5304) were associated with DTH under E1; significant difference were observed for DTH among genotypes with one, two, three and without any desirable alleles (Fig. 4). Similarly, for other four traits (DTM, PH, TGW, GYPP) also, significant joint effects were observed (Fig. 4).

On the basis of breeding value (estimated using effect of significant SNPs), contrasting genotypes were selected for each of the five traits under different environmental conditions (Supplementary Table S5). For three traits, including DTH, DTM and PH, genotypes with negative breeding value were considered as desirable, however, in case of TGW and GYPP, genotypes with positive breeding value were considered as desirable.

### Exploration of candidate gene (CG)

Significant MTAs identified during the present study were also used for identification of underlying CGs using Ensembl Plant database; for every MTA, a window of 1 Mb was used for identification of CGs. A search for CGs resulted in identification of 121 (ranges from 1 to 11 CGs/MTA) wheat CGs (Supplementary Table S6). This number of CGs was reduced to 68 genes using annotations based on gene ontology (GO), based on IWGSC RefSeq v1.0; the remaining 53 were placed in un-characterised category (Supplementary Table S6). Out of 68 characterized genes, 18 genes representing 16 MTAs were found to have putative role in drought stress on the basis of literature search (Table 2). These 18 CGs were involved in different biological activities like- transcription factors (AP2/ERF, bHLH, Dof, GRF, SBP, Zinc finger C2H2, WRKY), oxidoreductase activity, ubiquitination, trehalose-6 phosphatase activity, protein serine/threonine kinase activity, histone-lysine N-methyltransferase activity, amino-acid transmembrane activity (Table 2).

**Table 2:** Details of candidate genes (CGs) associated with significant MTAs and details of putative proteins identified from Ensembl wheat.

S. No.	Associated SNP ID (with trait and location details)	Chr; Genomic Location	Genes in 1 Mb region/Gene IDs (genomic location)	Description; Protein domain name/ID#	GO Annotation (GO IDs)		
					Biological process	Molecular function	Cellular component
1.	1003062 (DTH_E1, DTH_E2, DTM_E1, DTM_E2)	4B:39758253–39758321	TraesCS4B02G051500 (4B: 40,074,250–40,078,403)	Basic Helix-loop-helix DNA-binding domain (bHLH) (PF00010)	N/A	protein dimerization activity (GO: 0046983)	N/A
2.	1021420 (DTM_E4)	2A:755679307–755679375	TraesCS2A02G547600 (2A: 755,736,756–755,738,760)	Gibberellin-Dioxygenases Gene Family (GAox); Non-haem dioxygenase N-terminal domain/PF14226, Oxoglutarate/iron-dependent dioxygenase/PF03171	oxidation-reduction process (GO:0055114)	oxidoreductase activity (GO:0016491); 2. metal ion binding (GO:0046872	N/A
3.	1026541 (DTH_E2)	2B:772063506–772063574	TraesCS2B02G584900 (2B: 771,923,036–771,924,265)	F-box domain (IPR001810)	N/A	protein binding GO:0005515	N/A
4.	1050557 (DTH_E2)	5B:348450682–348450747	(a) TraesCS5B02G193100 (5B: 348,448,002–348,450,302)	Trehalose-phosphatase (PF02358)	trehalose biosynthetic process (GO:0005992); dephosphorylation (GO:0016311)	trehalose-phosphatase activity (GO:0004805); catalytic activity (GO:0003824); hydrolase activity (GO:0016787)	N/A
			(b) TraesCS5B02G193200 (5B: 348,570,451–348,572,272)	AP2/ERF gene (AP2 domain; PF00847)	transcription (GO: 0006351), regulation of transcription (GO: 0006355)	DNA-binding transcription factor activity (GO: 0003700)	Nucleus (GO:0005634)
5.	1063624 (DTH_E2, DTM_E1, DTM_E2)	3A:84182120–84182188	TraesCS3A02G116200 (3A: 83,977,019–83,983,489)	WRKY protein domain (PF03106)	regulation of transcription (GO: 0006355)	DNA-binding transcription factor activity (GO: 0003700)	Nucleus (GO:0005634)
6.	1065193 (TGW_E4)	3A:568445450–568445518	TraesCS3A02G323500 (3A: 568,094,977–568,098,215)	Bulb-type lectin domain (PF01453), S-locus glycoprotein domain (PF00954), PAN/Apple domain (PF08276), Serine-threonine/tyrosine-protein kinase, catalytic domain (PF07714)	protein phosphorylation (GO: 0006468)	protein serine/threonine kinase activity (GO: 0004674)	membrane (GO:0016020)
7.	1090844 (PH_E2)	5A:595082910–595082978	TraesCS5A02G401800 (5A: 594,770,622–594,772,285)	Dof domain, zinc finger (PF02701)	regulation of transcription (GO: 0006355)	DNA binding (GO:0003677)	Nucleus (GO:0005634)
8.	1091457 (GYPP_E3)	5D:391278717–391278776	TraesCS5D02G294400 (5D: 391,372,552–391,378,851)	Squamosa promoter binding protein domain (SBP; PF03110)	N/A	DNA binding (GO:0003677)	Nucleus (GO:0005634)
9.	1094628 (TGW_E2)	4A:667344925–667344993	(a) TraesCS4A02G389600 (4A: 666,980,143–666,983,528)	NB-ARC domain (PF00931)	N/A	ADP binding (GO: 0043531)	N/A
			(b) TraesCS4A02G389900 (4A: 667,174,383–667,174,854)	Cysteine-rich transmembrane CYSTM domain/PF12734	N/A	N/A	N/A
10.	1113894 (PH_E4)	1B:38599717–38599781	TraesCS1B02G055800 (1B: 38,832,030–38,835,862)	SET, SAD_SRA (PF02182); D2 Pre-SET (PF05033); D3, SET (PF00856)	Histone methylation (GO:0016571); Histone lysine methylation (GO:0034968)	Protein binding (GO:0005515); Zinc ion binding (GO:0008270); 3. Histone-lysine N-methyltransferase activity (GO:0018024)	Nucleus (GO:0005634); Chromosome (GO:0005694)
11.	1126379 (GYPP_E1)	4A:743768873–743768931	TraesCS4A02G498100 (4A: 743,748,235–743,749,019)	Late embryogenesis abundant protein (LEA2) (PF03168)	N/A	N/A	N/A
12.	1127414 (PH_E4)	3B:95751799–95751867	TraesCS3B02G123600 (3B: 95,459,819–95,462,522)	Zinc finger C2H2-type (IPR013087)	N/A	nucleic acid binding (GO:0003676)	N/A
13.	1134753 (DTH_E3)	3B:799744734–799744802	TraesCS3B02G568000 (3B: 799,521,193–799,524,789)	Ubiquitin (PF00240)	N/A	Protein binding (GO:0005515)	N/A
14	3023142 (DTH_E4)	5B:588883282–588883319	TraesCS5B02G414400 (5B: 588,831,208–588,832,584)	Cytochrome P450 (PF00067)	Oxidation-reduction process (GO:0055114)	Iron ion binding (GO:0005506); Oxidoreductase activity, acting on paired donors, with incorporation or reduction of molecular oxygen (GO:0016705)	N/A
15	977937 (TGW_E4)	2A:677497865–677497929	TraesCS2A02G422700 (2A: 677,530,470–677,532,528)	GRF zinc finger (PF06839)	N/A	Zinc ion binding (GO:0008270)	N/A
16	985312 (TGW_E2)	4B:37529691–37529759	TraesCS4B02G049000 (4B: 37,296,414–37,297,430)	Protein kinase domain (PF00069)	Protein phosphorylation (GO: 0006468)	protein kinase activity (GO:0004672); ATP binding (GO:0005524)	N/A

^#^Pfam database IDs. Chr., chromosome.

**Figure 5:**
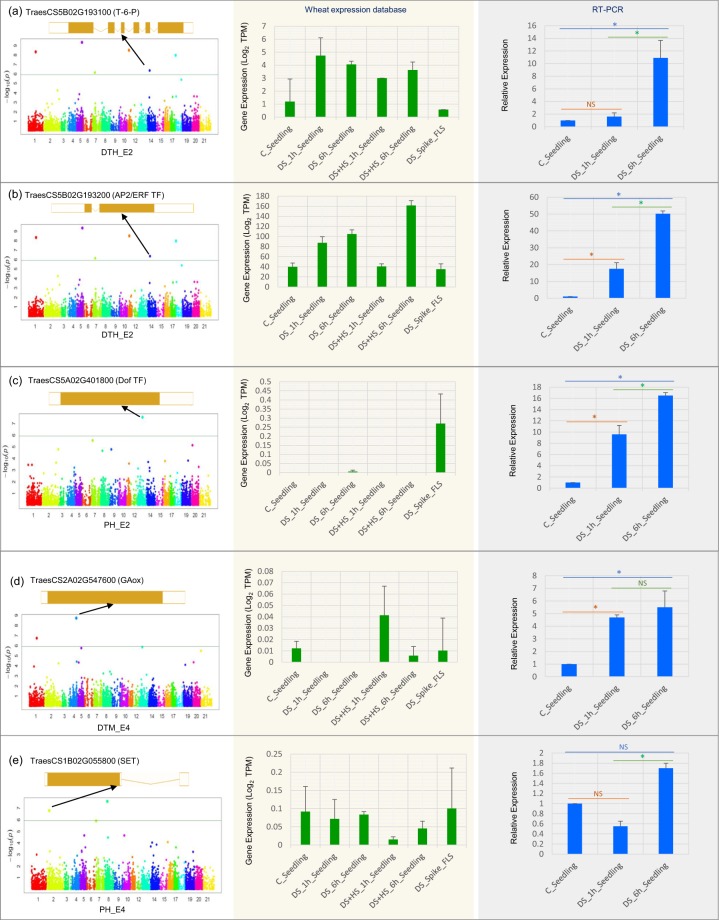
Manhattan plot showing candidate gene associated with MTA identified for different traits and expression profile of candidate genes (CGs) during drought stress in Wheat-Exp database and quantitative real-time PCR (qRT-PCR) analysis. (**a**) TraesCS5B02G193100 (Trehalose-6-Phosphate; T-6-P), (**b**) TraesCS5B02G193200 (APETALA2/Ethylene-responsive factor; AP2/ERF TF), (**c**) TraesCS5A02G401800 (DNA-binding One Zinc Finger; Dof TF), (**d**) TraesCS2A02G547600 (Gibberellin-dioxygenases; GAox), TraesCS1B02G055800 (Histone-lysine N-methyltransferase; SET). 2−ΔΔCt method was used to calculate the relative expression levels of the target genes. Statistically significant differences between control and treated were calculated based on Student’s t-tests: *p < 0.01, NS, not significant. DTH_E2, days to heading in Meerut rainfed environment; PH_E2, plant height Meerut rainfed environment; DTM_E4, days to maturity Powerkheda rainfed environment; PH_E4, plant height Powerkheda rainfed environment. C, control; DS, drought stress, HS, heat stress.

*In-silico* gene expression analysis was also conducted for the above mentioned 18 CGs using RNA‐Seq expression data from Wheat Expression Browser (http://www.wheat-expression.com/). This also provided further evidence of their potential involvement in the trait phenotype in different wheat developmental stages and tissues, and under drought stress condition (Supplementary Figs. S3 and S4). The results indicated variable expression of 10 of the 18 genes in different developmental stages and tissues. Four genes (TraesCS3B02G123600, TraesCS4A02G389900, TraesCS5B02G193100, TraesCS5B02G294400) had relatively higher expression (up to 3.6 Transcripts Per Million; TPM) in all tissues and at all developmental stages. Some CGs expressed uniquely in a specific tissue or developmental stages (Figure Supplementary Fig. S3). For example, TraesCS3A02G116200 expressed in stem tissues at all developmental stages; TraesCS3A02G323500 expressed in root tissues at all developmental stages and TraesCS5B02G414400 expressed in the spikes at reproductive stage (Supplementary Fig. S3). Under drought stress condition, only five of the 18 CGs showed higher expression (Supplementary Fig. S4). Interestingly, out these five CGs, four CGs (TraesCS3B02G123600, TraesCS4A02G389900, TraesCS5B02G193100, TraesCS5B02G193200, TraesCS5D2G294400) belong to MTAs that were identified only in RF environments (Table 2; Supplementary Fig. S4). To further ascertain the biological functions under DS (1 h and 6 h) of the above CGs, we examined their expression profiles using quantitative real-time PCR (qRT-PCR) analysis. Out of five gene examined, following four CGs showed higher expression under DS: (i) TraesCS5B02G193100 (Trehalose-6-Phosphate;T-6-P), (ii) TraesCS5B02G193200 (APETALA2/Ethylene-responsive factor; AP2/ERF TF), (iii) TraesCS5A02G401800 (DNA-binding One Zinc Finger; Dof TF) and (iv) TraesCS2A02G547600 (Gibberellin-dioxygenases; GAox)]; and remaining solitary CG [TraesCS1B02G055800 (SET)] did not show any significant change in its expression under DS as compared to control (Fig. 5). We also evaluated the correlation between RNA-Seq data (using log2 TPM values) and RT-qPCR data (using normalized Ct values) for the above mentioned five genes. As expected, significant negative correlation was observed between the two values (R^2^ = 0.795, P < 0.001).

### Identification of putatively important rare variants for GY

During GWAS rare variants (Minor allele frequency; MAF < 0.05) are mostly ignored due to statistical reasons. However, in order to examine the importance of rare variants in determining phenotypic variation in the present study, we selected 99 SNPs with MAF ranging from 0.03 to 0.04, and tested each individual SNP for association with GYPP in all the four environments using “t test”. Total seven SNPs were found associated with GYPP in E1 and E2 environments. These included two SNPs each uniquely identified in each of the E1 and E2 and three SNPs that were found in both the E1 and E2 environments (Supplementary Table S7). In the remaining two environments (E3 and E4), no significant association was observed. Under E1, out of five significant SNPs, major alleles for four SNPs were desirable (i.e. having higher mean value for GYPP) and the minor allele of the remaining one SNP was associated with higher mean GYPP. However, under E2, the major alleles for each of the five significant SNPs were associated with higher GYPP.

## Discussion

As mentioned earlier, studies on genetics of drought tolerance have been conducted in several crops including wheat. As a result, it is also widely known that drought tolerance is a complex quantitative trait that is influenced by genetic background and environmental conditions. In the present study, we evaluated a WAM panel [assembled at the International Maize and Wheat Improvement Center (CIMMYT) gene bank] under different water regimes in different locations of India in order to conduct GWAS. This diverse panel was never utilized earlier for a study of the genetics of drought tolerance using GWAS, although it was utilized for conducting GWAS for other traits like yield related trait^26^ and Fe, Zn, β-carotene, GPC content^27^. The study allowed us to identify important genomic regions carrying some important genes associated with drought tolerance. Five yield and related traits were used for recording data on drought tolerance. High variability (as revealed by descriptive statistics) for each of the five traits under IR/RF environments suggested that the panel was suitable for a study of the genetics of quantitative traits like yield. Moreover, genetic diversity and PIC (based on marker data) also suggested that WAM panel is highly diverse. Significant and strong correlation of four traits (PH, DTH, DTM, and TGW) with GY under IR and RF environments (Supplementary Table S2) suggested that these traits may be used as surrogate for yield under different water regimes. The D sub-genome carried only one-third or one- fourth MTAs (1157) identified on A and B sub-genomes (3969 and 4501). This is attributed to relatively low level of diversity observed in the D sub-genome that is generally attributed to late hybridization of *Aegilops tauschii* during evolution of common wheat^35,38,39^.

During present study, we utilized multi-locus association model (consider background loci during association testing) for GWAS to overcome the limitations arising due to single locus GWAS^40,41^. Being a multi-locus model, confounding arising due to population structure may also be corrected by including kinship (K-model) and principal components (Q-model) in association test model. During present study, appropriateness of multi-locus association model was confirmed by solid lines in quantile-quantile (Q-Q) plots (Supplementary Fig. S2). Q-Q plots also suggested that power of test statistics were high. Moreover, to reduce false positives due to multiple testing Bonferroni correction criteria was used and 46 high confidence MTAs (ranging from 5MTAs for PH to 14 MTAs for DH) were identified for five traits under the two water regimes (Table 1). Interestingly, for almost all the traits, significant joint effect of the desirable alleles of all the SNPs involved in different MTAs was observed suggesting that identified MTAs are important and corresponding traits may be improved significantly through pyramiding of significant SNP alleles (Fig. 4). The identified MTAs also add up to the existing knowledge and may be useful for downstream research and also for wheat breeding. MTAs identified uniquely under IR (20) or RF (19) environments may be important to understand the genetics of water stress signalling, however, MTAs that were common in both the IR and RF environments may be considered as independent of water level and may be found useful for breeding under different water regimes.

The 46 MTAs identified in this study were also compared to MTAs/QTL identified in earlier important studies using chromosome position, where the mapping population/germplasm/association panel were phenotyped under different water regimes (Table 3). Five MTAs were co-localized with QTL/MTAs identified in earlier studies using linkage mapping and GWAS, and the remaining 41 MTAs are novel. Therefore, it seems that the WAM panel used during the present study is quite diverse from the genetic material used in earlier studies. Among the co-localized MTAs/QTL, an MTA associated with GYPP and located on chromosome 7A was co-localized with MTAs/QTL for GY and TGW identified in four earlier studies^28,42–44^. Out of above co-localized QTL/MTAs, a QTL *Qyld.csdh.7AL* was associated with GY under RF environments^42^; the other three co-localized MTAs were earlier identified using GWAS. For instance, a genomic region between 148.43–161.31 cM (wsnp_CAP7_c1321_664478-IACX7848) was associated with GY under DS^28^. The other two co-localized genomic region between 150.54–178.42 cM (wsnp_Ex_c11047_17915103-wsnp_Ku_c8437_14341371) and at 156.23 cM (Excalibur_c14451_1313) were associated with TGW^43,44^. Another MTA associated with TGW and located on chromosome 6A at 88.9 cM was co-localized with a QTL for TGW (*QTkw.aww.6A*) associated with wmc0256A mapped at 90 cM^45^. Similarly, the other important MTA associated with TGW identified in RF environment during the present study and located on chromosome 2A at 150.26 cM was co-localized with an MTA for GY under DS located at 149 cM^28^. The above three genomic regions (on 2A, 6A and 7A) associated with GY under drought stress identified during the present study could be subjected to fine mapping and CG identification, so that diagnostic molecular markers can be developed for deployment in breeding programs, particularly those targeting drought prone regions.

**Table 3:** Comparison of MTAs identified in present study with QTL/MTAs reported in earlier studies.

MTAs in present study			Previous Study			References
Marker Associated/Clone ID	Chr.; Pos.(cM)	Trait	Marker Associated/linked	Chr.; Pos. (cM)	Trait	
SNP_1829/977937	2A; 150.126	TGW	Excalibur_c52319_257 (AM)	2A; 149	GY under DS	^28^
SNP_3398/1063624	3A; 75.969	DTH, DTM	wmc264 (AM)	3A; 75	DTH	^22^
SNP_4022/1127414	3B; 94.818	PH	Xbarc157 (AM)	3B; 104	PH under DS	^22^
SNP_7088/1073429	6A; 88.944	TGW	QTkw.aww.6A; wmc0256A (LM)	6A; 90	TGW	^46^
SNP_8465/1093542	7A; 157.225	GYPP	Qyld.csdh.7AL; wmc273.3 (LM)	7A; 159	GY under DS	^43^
			wsnp_Ex_c11047_17915103 - wsnp_Ku_c8437_14341371 (AM)	7A; 150.54–178.42)	TGW	^44^
			Excalibur_c14451_1313 (AM)	7A; 156.23	TGW	^45^
			wsnp_CAP7_c1321_664478~IACX7848 (AM)	7A; 148.43–161.31	GY under DS	^28^

DTH, days to heading; DTM, days to maturity; GY, grain yield; GYPP, grain yield per plot; PH, plant height; TGW, thousand grain weight; DS, drought stress; AM, association mapping; LM, linkage mapping, Chr., chromosome, Pos., position.

It is well known that wheat genome is allopolyploid with three sub-genomes (A, B and D); and thus, most of wheat genes (including CGs identified in present study) have three homoeologous copies one on each sub genomes (i.e. A, B, D sub-genome). Polyploidization may provide sub-functionalization or neo-functionalization against stress to plant^46^. Gene involved in drought tolerance also found to have homologous copies, and majority of them showed expression partitioning under stress condition to provide better adaptability and wide distribution of wheat^46^.

Further, in comparison to the single SNPs, haplotypes involving multiple SNPs may be more useful in plant breeding^47^. Haplotype analysis using multiple significant SNPs was recently reported in cotton^48,49^ and foxtail millet^50^ and desirable haplotypes impacting multiple traits were identified. Such haplotypes may prove useful in improvement of multiple associated traits simultaneously. During the present study, the haplotypes H3 (GR3) was found under rainfed environment only and this haplotype was associated with higher TGW and early maturity. Grain weight is a highly heritable trait and has high phenotypic stability^51^ and early maturity under rainfed environment allows the crop to avoid the ever-depleting soil moisture during the crop growth. Therefore, haplotype H3 may be exploited in MAS for breeding for water stress tolerant wheat genotypes. Under IR environments, the haplotypes H3 (GR4) and H8 (GR5) showed association with early maturity, reduced plant height, higher TGW and GYPP. These two haplotypes may be exploited for breeding high yielding early maturing wheat varieties for IR environments.

An effort was also made to identify CGs underlying MTAs. For this purpose, expression analysis of identified CGs was examined. The results suggested that the following four genes were strong candidates for the MTAs identified [TraesCS5B02G193100 (T-6-P), TraesCS5B02G193200 (AP2/ERF TF), TraesCS5A02G401800 (Dof TF), TraesCS2A02G547600 (GAox)]: Following are the reasons for the high level of confidence: (i) each of these genes exhibited a significantly higher expression under DS; (ii) MTAs linked with these CGs were identified exclusively in RF environments (Table 2, Fig. 5). Among these four genes, the gene TraesCS5B02G193100 encodes trehalose 6-phosphatase (T6P), which regulates carbon assimilation and sugar status in plants. In addition, T6P has also been demonstrated to play an essential role in plant development under drought stress^52,53^. A wheat TPP (T6P) was also found to be associated with TGW in bread wheat^54^. The second gene TraesCS5B02G193200 encodes APETALA2/Ethylene-responsive factor (AP2/ERF), which is a transcription factor. Genes belonging to this family of TFs are mainly plant-specific TFs and are known to be involved in regulation of tolerance to several abiotic stresses including DS^55,56^. Another important CG TraesCS5A02G401800 encodes DNA-binding One Zinc Finger (Dof) TF; this family of TFs contains a zinc finger domain, and plays an important role in imparting tolerance against DS in higher plants^57^. DoF TF was initially reported in maize (*ZmDOF1*), where it plays a major role in light-regulated gene expression^57^. In wheat, *Dof1* has been shown to be involved in carbon metabolism by increasing the regulation of the C4 pathway^58^. Another wheat Dof gene *WPBF* has been reported to be involved in plant growth and development^59^. Recently in potato (*Solanum tuberosum* L.), a *Dof* gene (PGSC0003DMG400019528) was reported to be upregulated in response to 2 h DS^60^. The fourth gene, TraesCS2A02G547600 encodes Gibberellin-dioxygenases (GAox) that are involved in the biosynthesis of bioactive gibberellins (GAs). Abundance of GA regulates responses to environmental stress including DS^61^. GAox genes are also indirectly involved in DS regulation *via* GA biosynthesis^61^. Also, the role of these genes in drought tolerance in wheat is supported by their up-regulation in wheat seedling under DS (Fig. 5). Gene-based functional markers for these genes may be developed and used for molecular breeding to increase wheat yield under drought stress.

During present study, we also analysed seven important SNPs (out of 99 SNPs with MAF) that were associated with GY; these were eliminated during GWAS due to MAF. The minor allele of one (SNP_6794) of the seven SNPs was associated with significantly higher GYPP under IR environment. Similar to the present study, earlier also, some rare alleles were also found to be desirable for important agronomic traits in wheat^31^. In rice also, rare allele of important grain size gene *GS2* was found to increase the grain size and yield^62^. Thus, we strongly feel that rare variants should not be ignored as a whole during GWAS since some of the rare alleles may be responsible for important traits.

In summary, MTAs identified during present study may be further validated using joint linkage and association mapping (JLAM) or post-GWAS^63^. Further, desirable alleles of associated SNPs, desirable haplotypes and CG-based markers together may prove useful in the breeding for improved wheat varieties; the CGs may also be validated using functional genomics approaches and CG-based association mapping. Significant joint effect of associated SNPs suggested that corresponding traits can be improved substantially through pyramiding of significant SNP alleles. Contrasting genotypes identified during the present study may serve as a promising material to develop mapping populations for further genetic dissection of the trait; and favourable genotypes may serve as donor in a breeding program.

## Material and Methods

### WAM panel and field experiment

The wheat association mapping (WAM) panel for GWAS consisting of 320 diverse spring wheat genotypes was assembled at the International Maize and Wheat Improvement Center (CIMMYT) (details of 320 genotypes are available in Supplementary Table S8). This WAM panel was evaluated for two consecutive years (2011–12 and 2012–13) at two locations in India (Meerut, Uttar Pradesh and Powerkheda, Madhya Pradesh, separated by >900 km) in 18 × 18 simple lattice design (two replications) independently in IR and rainfed environments, making a total of four environments. In a particular environment, each genotype in a replication was raised in plots of 3 rows of 1.5 m each with a row to row distance of 0.25 m. In IR environments, five irrigations were given, four before flowering and one during the grain filling duration. In the rainfed environment, single irrigation was given at 21 days after sowing to allow crop establishment and to avoid complete crop failure. The details of the experimental sites, planting dates of experiments, meteorological data and rainfall during the crop seasons are summarized in Table 4.

**Table 4:** Details of the environments, dates of planting of experiments, coordinates, altitude, mega-environment (ME), total rainfall, average maximum and average minimum (avg max/avg min) temperatures of the four environments used to evaluate WAM panel.

Crop-season	Locations	Water condition	Planting date	Coordinates	Altitude (M)	ME*	Avg max/avg min (°C)	Rainfall (mm)^#^
2011–12	Meerut, India	IR	Nov 16	28°0.97′N77°0.74′E	227	ME1	25.8/11.6	73.7
	Meerut, India	RF	Nov 16	28°0.97′N77°0.74′E	227	ME1	25.8/11.6	73.7
2012–13	Powerkheda, India	IR	Nov 21	22°0.07′N73°0.98′E	304	ME5	30.6/11.3	63.8
	Powerkheda, India	RF	Nov 21	22°0.07′N73°0.98′E	304	ME5	30.6/11.3	63.8

IR, irrigated; RF, rainfed; *Source is CIMMYT wheat atlas, http://old.wheatatlas.org/; ME, mega-environments; Avg, Average; ^#^Total rain-fall during the crop-season in mm.

### Phenotyping

Data on each of 320 genotypes were recorded for the following five traits: (i) days to heading (DTH), recorded as number of days from the date of planting until the emergence of spikes in ~75% of plants in a plot; (ii) days to maturity (DTM), recorded as the number of days from the date of planting until physiological maturity achieved in ~75% of the spikes in a plot, (iii) plant height (PH), recorded at maturity as the mean height (from ground to tip of the main spike) of randomly selected five plants, (iv) thousand grain weight (TGW), recorded as average weight (g) of five random samples of 1000 grains each; (v) grain yield per plot (GYPP), recorded as grain weight of plot.

### Genotyping by sequencing and SNP markers

Genomic DNA was extracted from fresh leaves collected from five plants per line using a modified CTAB method^64^. Genotyping was undertaken using GBS developed by DArT Pty. Ltd., Yarralumla, Australia. The detailed methodology is described elsewhere^26^.

### Genetic diversity, population structure and LD

Using mapped SNPs (MAF > 0.05%; missing data <30%), diversity analysis was conducted, and two diversity parameters, namely gene diversity^65^ and PIC^66^ were calculated at chromosome, sub-genome and whole genome levels. Model-based cluster analysis was performed using 42 unlinked SNPs, one from each of the 42 chromosome arms, to infer population structure in the dataset. The software STRUCTURE version 2.2^67^ was used for this purpose. The number of presumed sub-populations (K) was set from 2 to 20, and the process was repeated three times. For each run, burn-in and MCMC iterations were set to 50,000 and 100,000, respectively, and a model “without admixture and correlated allele frequencies” was used. The number of sub-populations was determined following delta K (ΔK) method^68^. Whole genome as well as chromosome wise LD was conducted as a part of another study using TASSEL 5, and decay distance was calculated using R software^27^.

### MTAs and haplotype analysis

During the present study we used a multi locus GWAS model named Fixed and random model Circulating Probability Unification (FarmCPU)^40^. This method is believed to be highly efficient in computation and also eliminates confounding issues arising due to population structure, kinship, multiple testing correction, etc. This method utilizes both Fixed Effect Model (FEM) and a Random Effect Model (REM), iteratively. REM tests estimate pseudo-quantitative trait nucleotides (QTNs), FEM tests marker using pseudo-QTNs as covariates. For GWAS, SNPs having <30% missing data and >5% minor allele frequency were utilized. Principle component analysis (PCA) was conducted using TASSEL 5.0, and first three components were incorporated as covariate in association test model. Bonferroni-corrected P-value threshold was set as 0.01 (−log p value = 6.0) and SNP with −log (p) > 6.0 declared as significant MTA. Q-Q plots generated through FarmCPU were used to examine model fitting (account for population structure). Haplotypes were determined on the basis of interval distance between associated SNPs. Significant SNPs present within LD range (10 cM) were considered as one haplotype. Univariate GLM was used to determine significance difference for trait among haplotypes.

### Joint effect of SNPs, breeding values and contrasting phenotype

Joint effects were estimated, when more than two SNPs were associated with the same trait. To determine joint effect, linear regression was performed using number of desirable SNP alleles for traits (independent variable) and corresponding trait values of the genotypes that contained more than one desirable SNP alleles (dependent variable). The breeding value of each accession was calculated from the allelic effects of all significant SNPs. The allelic effect was calculated by taking the difference in trait value between genotypes with contrasting alleles. The breeding value for each genotype was estimated, by using absolute value of the allelic effect of each significant SNP taken as a negative value if a genotype, had SNP allele that decreases the trait value and *vice-versa*. All positive and negative allelic values were summed to estimate the breeding value of each genotype. For each trait, contrasting phenotypes were selected with minimum and maximum breeding value.

### Identification of putative CGs and their expression analysis

CGs for individual MTAs were identified by aligning the associated GBS sequences to wheat genome assembly IWGSC1.0^69^ available on the Ensemble database (http://www.ensembl.org/info/docs/tools/vep/index.html). High-confidence annotated genes were retrieved from a 1000 kb window for each identified MTA. The GO annotation information of these CGs were extracted from the IWGSC website (http://www.wheatgenome.org/). For annotated CGs, gene expression analysis was conducted utilizing wheat expression database^70^ hosted at http://wheatexpression.com. Transcripts per kilobase millions (TPM) values for every CG were downloaded. Log transformed (Log2X) value was used to generate a heatmap using online tool ClustVis^71^.

### Plant materials, treatments, and quantitative real-time PCR (qRT-PCR) analysis

Seedlings of wheat cv. ‘C306’ representing the well-known drought tolerant genotype were analysed for tolerance against DS. For applying water stress, 7 days old seedlings were transferred to modified Hoagland’s solution (+20% PEG 8000). The leaf samples of control and treated seedlings were harvested at 0, 1 and 6 h after water stress treatment and immediately frozen in liquid nitrogen and stored at −80 °C. Total RNA was isolated using the TRIzol Reagent (Ambion) as per the manufacturer’s specifications. First-strand cDNA was synthesized from DNaseI-treated total RNA using RevertAid First Strand cDNA Synthesis Kit (Thermo Scientific) according to the manufacturer’s instructions. qRT-PCR was performed in step-plus Real-Time PCR System (Applied Biosystems) using the SYBR Green (Applied Biosystem). Wheat β-actin gene was used as an endogenous control. The primers of CGs used for qRT-PCR are listed in Supplementary Table 9. Relative gene expression levels were calculated according to the 2−ΔΔCt method^72^. All the samples were analyzed with two biological and three technical replicates. Student’s t-test was applied to see the significant differences between the control and treatments.

## Supplementary information

Supplementary Information
