# Supplementary material for: Multi-Locus Genome Wide Association Mapping for Yield and Its Contributing Traits in Hexaploid Wheat under Different Water Regimes
Source: Sci Rep. 2019 Dec 20;9:19486. doi: 10.1038/s41598-019-55520-0 (PMC6925107; doi:10.1038/s41598-019-55520-0)
Supplement: Supplementary file 1 — Supplementary Information [file 41598_2019_55520_MOESM1_ESM.pdf]

Multi-Locus Genome Wide Association Mapping for Yield and Its Contributing Traits in Hexaploid Wheat under Different Water Regimes

Vijay Gahlaut[1,2,5], Vandana Jaiswal[1,3,5], Sukhwinder Singh[4], H.S. Balyan[1] and P.K. Gupta[1*]

[1] Department of Genetics and Plant Breeding, Ch. Charan Singh University, Meerut, India
[2] Department of Plant Molecular Biology, University of Delhi, South Campus, New Delhi, India
[3] School of Life Sciences, Jawaharlal Nehru University, New Delhi, India
[4] CIMMYT, Mexico DF, Mexico

* Corresponding author: P.K. Gupta Email: pkgupta36@gmail.com
[5]these authors contributed equally to this work

Supplementary Information

Supplementary Figure S1. Genetic relatedness of 320 wheat accessions determined using 42 SNP markers through STRUCTURE analysis. Numbers on the y-axis indicate the membership coefficient. The colour of the bar indicates the three sub-populations identified through the STRUCTURE program (G1=red, G2=green, G3=blue). Accession with a similar colour belong to the same group. Accessions with 2-3 colours indicate admixture.

Supplementary Figure S2. Manhattan plots and quantile-quantile (Q-Q) plots (a to t) of the GWAS results for days to heading (DTH), days to maturity (DTM), plant height (PH), thousand grain weight (TGW) and grain yield/plot (GYPP) in four environments (E1, Meerut irrigated; E2, Meerut rainfed; E3, Powerkheda irrigated; E4, Powerkheda rainfed). Significant MTA threshold [– log 10 (p) $<10^{-06}$] are represented by green lines.

Supplementary Figure S3. Heat map showing expression levels of 18 putative candidate genes at different wheat developmental stages and tissues. Log transformed (Log2X) transcripts per kilobase millions (TPM) values for every candidate gene was used to generate a heat map.

Supplementary Figure S4. Heat map showing expression levels of 18 putative candidate genes at different wheat tissues under drought stress. Log transformed (Log2X) transcripts per kilobase millions (TPM) values for every candidate gene was used to generate a heat map.

Supplementary Table S1. Descriptive statistics for five yield and its related traits of the SWRS in four environments. DTH, days to heading; DTM, days to maturity; PH, plant height; TGW, thousand grain weight; GY, grain yield per plot * E1, Meerut irrigated; E2, Meerut rainfed; E3, Powerkheda irrigated; E4, Powerkheda rainfed.

Supplementary Table S2. Pearson's correlation coefficients (r-values) among five yield and its related traits. Values above the diagonal indicate r-values in irrigated environment; values below the diagonal indicate r-values in rainfed environments; ** Significant at P=0.001; DTH, days to heading; DTM, days to maturity; PH, plant height; TGW, 1000 grain weight; GYPP, grain yield /plot.

Supplementary Table S3. Distribution of 9,627 SNPs on 21 wheat chromosomes, density of SNPs/10cM and the length of individual chromosomes in cM. Chr., chromosome

Supplementary Table S4. Gene diversity and polymorphic information content (PIC) of 21 wheat chromosomes.

Supplementary Table S5. List of contrasting genotypes selected based on breeding value and number of favourable alleles for each of the five traits considered during present study. Gen., Genotype; Fav., Favourable; BV, Breeding value

Supplementary Table S6. Details of 121 genes associated with significant MTAs identified using Ensembl Plant database (a window of 1 Mb was used for identification of CGs). Details of putative proteins identified from Ensembl wheat also given. # Pfam database IDs. Chr., chromosome.

Supplementary Table S7. List of SNPs having rare variant (minor allele frequency <0.05) showing significant difference (using t-test) for grain yield (GY) between genotypes with contrasting SNP alleles.

Supplementary Table S8. Details of the spring wheat reference set (SWRS) accessions used in the present study.

Supplementary Table S9. Primer sequences of genes utilized in qRT-PCR expression analysis.

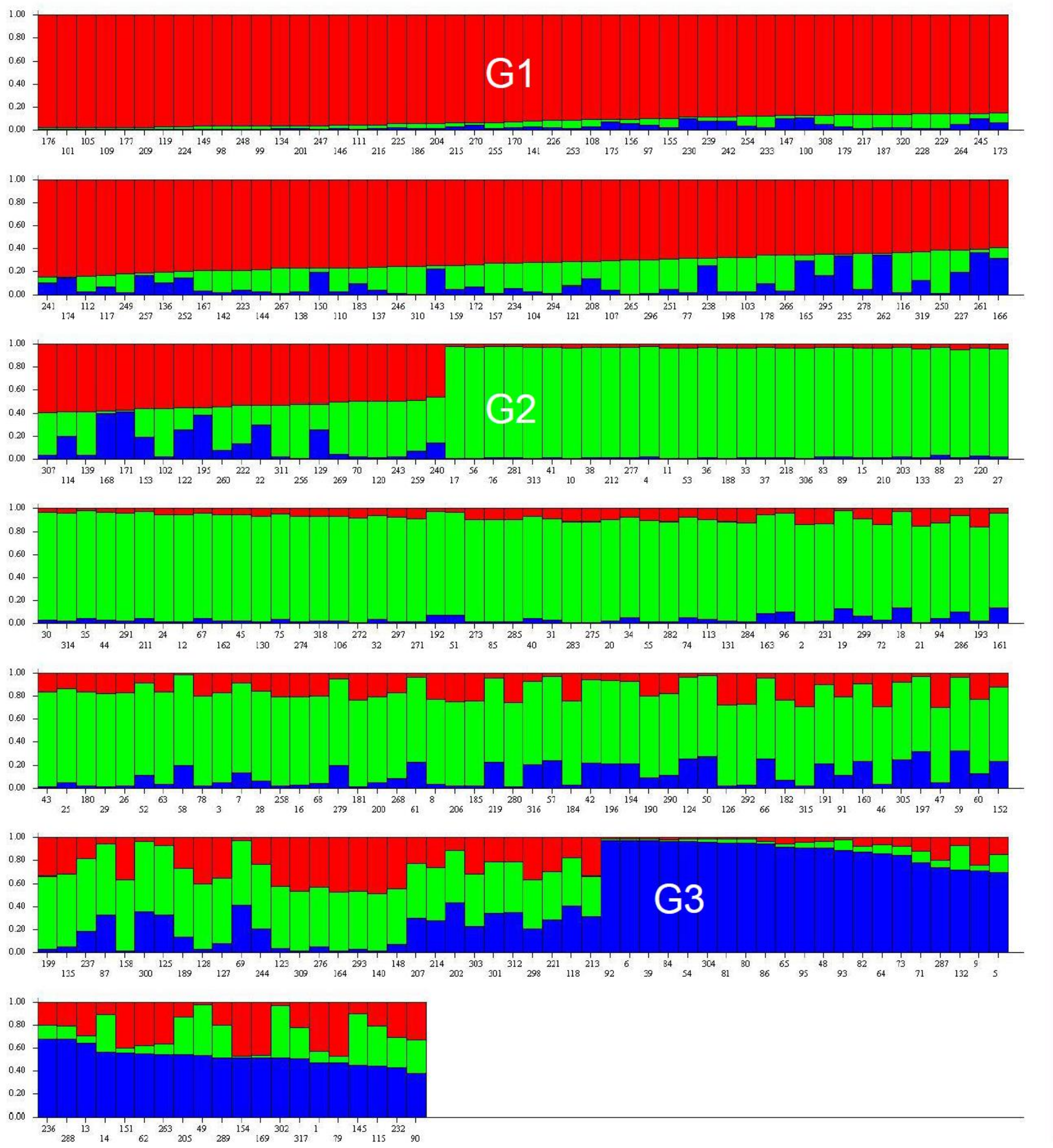

Supplementary Figure S1. Genetic relatedness of 320 wheat accessions determined using 42 SNP markers through STRUCTURE analysis. Numbers on the y-axis indicate the membership coefficient. The colour of the bar indicates the three sub-populations identified through the STRUCTURE program (G1=red, G2=green, G3=blue). Accession with a similar colour belong to the same group. Accessions with 2-3 colours indicate admixture.

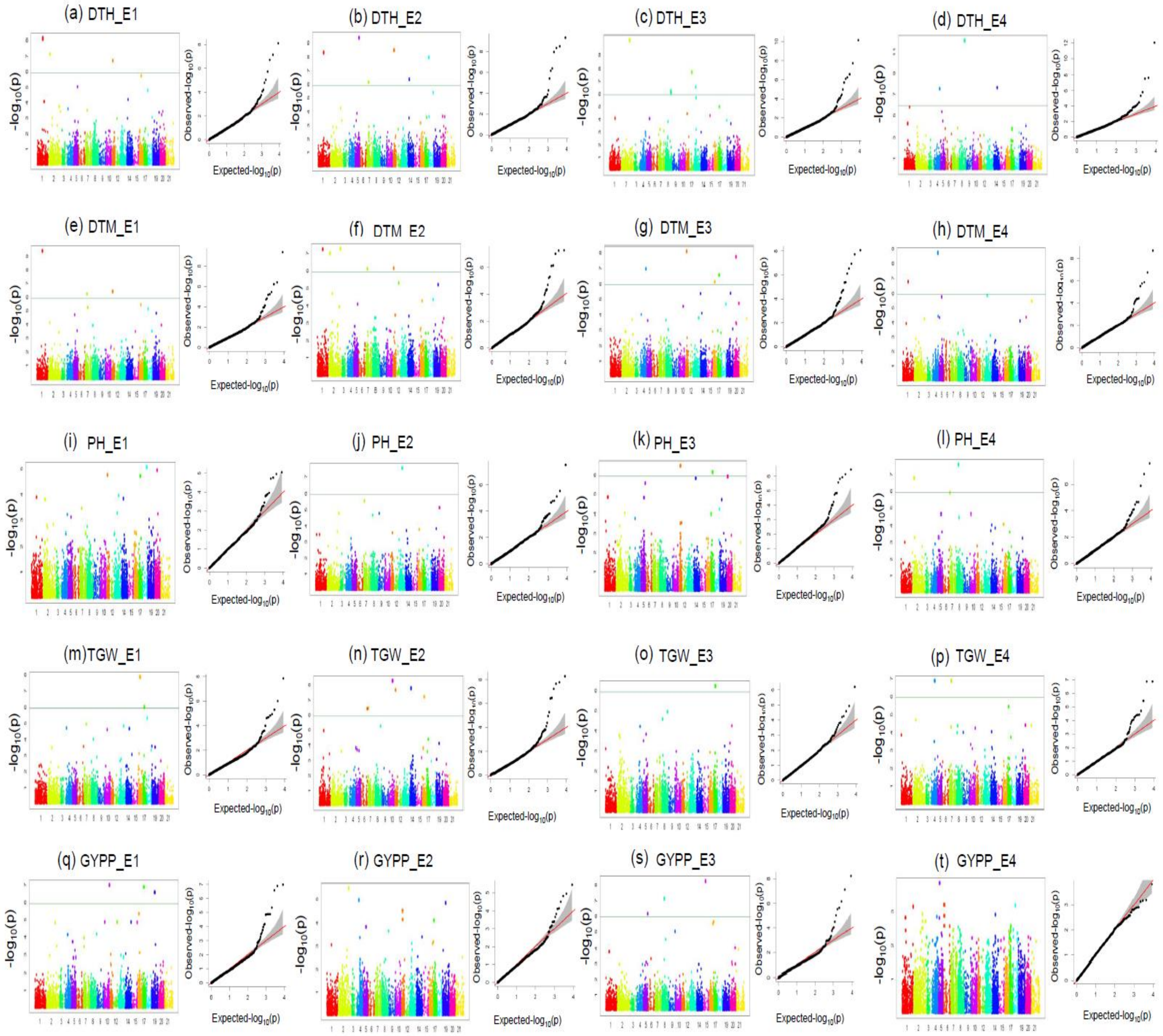

Supplementary Figure S2. Manhattan plots and quantile-quantile (Q-Q) plots (a to t) of the GWAS results for days to heading (DTH), days to maturity (DTM), plant height (PH), thousand grain weight (TGW) and grain yield/plot (GYPP) in four environments (E1, Meerut irrigated; E2, Meerut rainfed; E3, Powerkheda irrigated; E4, Powerkheda rainfed). Significant MTA threshold [– log 10 (p) $<10^{-06}$] are represented by green lines

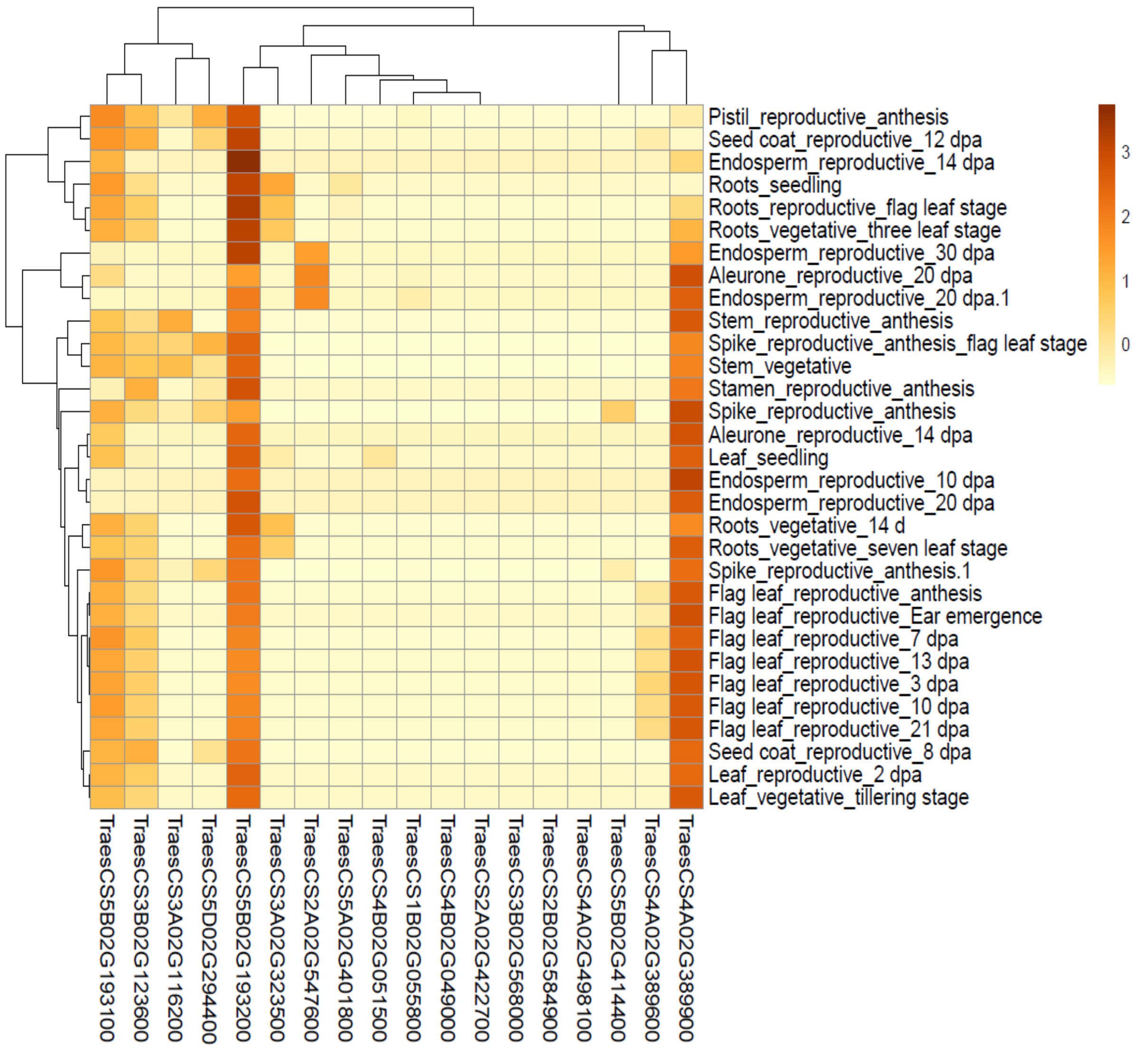

Supplementary Figure S3. Heat map showing expression levels of 18 candidate genes at different wheat developmental stages and tissues. Log transformed (Log2X) transcripts per kilobase millions (TPM) values for every candidate gene was used to generate a heat map.

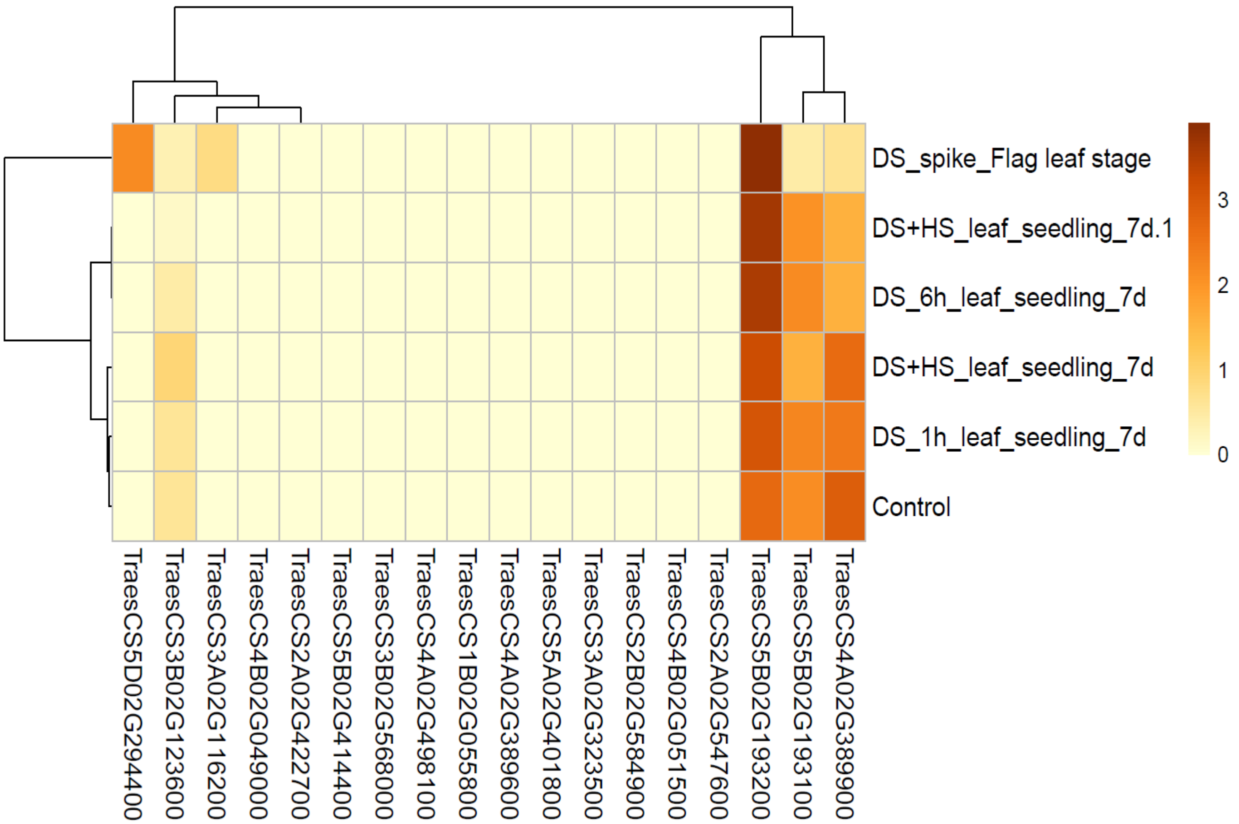

Supplementary Figure S4. Heat map showing expression levels of 18 candidate genes at different wheat tissues under drought stress. Log transformed (Log2X) transcripts per kilobase millions (TPM) values for every candidate gene was used to generate a heat map.

Trait	Environment*	Minimum	Maximum	Mean	SD	Skewness	Kurtosis	CV (%)
DTH	E1	82.5	130.0	99.7	12.4	0.8	-0.7	12.4
	E2	79.0	126.5	97.5	11.1	0.7	-0.6	11.4
	E3	78.5	118.0	92.5	8.1	1.0	0.6	8.7
	E4	64.0	113.0	89.4	8.9	0.7	0.0	10.0
DTM	E1	124.0	161.0	138.7	10.0	0.7	-0.9	7.2
	E2	119.0	156.5	133.7	9.7	0.8	-0.8	7.2
	E3	120.5	156.0	133.8	7.6	0.8	0.1	5.6
	E4	65.5	150.0	126.0	8.5	-0.7	7.5	6.7
PH (cm)	E1	69.4	147.2	106.2	16.5	0.5	-0.6	15.5
	E2	55.8	125.2	89.2	11.7	0.3	-0.3	13.1
	E3	56.5	134.0	96.4	16.0	0.4	-0.6	16.6
	E4	47.3	116.8	79.3	13.1	0.2	-0.1	16.5
TGW (g)	E1	17.2	53.8	35.9	7.3	-0.2	-0.4	20.5
	E2	13.2	49.4	32.2	7.3	-0.3	-0.4	22.6
	E3	21.4	69.5	43.0	8.4	-0.2	-0.3	19.5
	E4	12.7	60.4	38.8	7.6	-0.4	0.4	19.7
GYPP (g)	E1	75.0	745.0	404.6	141.4	0.0	-0.7	34.9
	E2	35.0	517.5	219.9	87.3	0.2	-0.2	39.7
	E3	111.0	661.5	353.2	101.0	0.2	-0.1	28.6
	E4	13.0	500.0	199.8	64.5	0.6	2.0	32.3

Supplementary Table S1. Descriptive statistics for five yield and its related traits of the SWRS in four environments. DTH, days to heading; DTM, days to maturity; PH, plant height; TGW, thousand grain weight; GY, grain yield per plot * E1, Meerut irrigated; E2, Meerut rainfed; E3, Powerkheda irrigated; E4, Powerkheda rainfed.

	Meerut					Powerkheda				
	DTH	DTM	PH	TGW	GYPP	DTH	DTM	PH	TGW	GYPP
DTH	1	.977**	.640**	-.643**	-.676**	1	.879**	.320**	-.486**	-.317**
DTM	.949**	1	.663**	-.661**	-.698**	.862**	1	.336**	-.528**	-.321**
PH	.392**	.392**	1	-.344**	-.513**	.356**	.388**	1	-.286**	-.281**
TGW	-.697**	-.696**	-.278**	1	.577**	-.421**	-.355**	-.150**	1	.290**
GYPP	-.686**	-.709**	-.235**	0.558**	1	-.178**	-.198**	-0.044	.238**	1

Supplementary Table S2. Pearson's correlation coefficients (r-values) among five yield and its related traits. Values above the diagonal indicate r-values in irrigated environment; values below the diagonal indicate r-values in rainfed environments; ** Significant at P=0.001; DTH, days to heading; DTM, days to maturity; PH, plant height; TGW, 1000 grain weight; GYPP, grain yield /plot.

Chr.	Sub-genome A			Sub-genome B			Sub-genome D		
	No. of SNP	Length (cM)	Density/10 cM	No. of SNP	Length (cM)	Density/10 cM	No. of SNP	Length (cM)	Density/10 cM
1	603	491.2	12	722	561.1	13	172	267.0	6
2	617	261.5	24	874	209.0	42	197	314.3	6
3	605	278.6	22	789	299.8	26	207	296.1	7
4	471	250.6	19	282	153.8	18	77	189.6	4
5	484	299.1	16	641	296.2	22	116	256.4	5
6	472	196.7	24	590	172.0	34	152	211.9	7
7	717	306.5	23	603	261.4	23	236	370.3	6

Supplementary Table S3. Distribution of 9,627 SNPs on 21 wheat chromosomes, density of SNPs/10cM and the length of individual chromosomes in cM. Chr., chromosome.

Chromosome	Sub-genome A		Sub-genome B		Sub-genome D	
	Gene diversity	PIC	Gene diversity	PIC	Gene diversity	PIC
1	0.07	0.28	0.32	0.26	0.26	0.21
2	0.34	0.27	0.34	0.27	0.23	0.20
3	0.33	0.27	0.32	0.26	0.19	0.17
4	0.34	0.27	0.29	0.24	0.18	0.15
5	0.32	0.26	0.35	0.28	0.21	0.18
6	0.35	0.28	0.33	0.26	0.28	0.23
7	0.32	0.26	0.34	0.27	0.22	0.18
Average	0.30	0.27	0.33	0.26	0.22	0.19

Supplementary Table S4. Gene diversity and polymorphic information content (PIC) of 21 wheat chromosomes.

Trait	Environment	Favourable				Unfavourable			
		Gen.	Trait value	BV	No of fav. allele	Gen.	Trait value	BV	No of fav. allele
DTH	E1	TX308	82.5	-13.9	3	TX49	126.0	13.9	0
	E2	TX317	79.0	-20.4	6	TX48	117.5	6.8	2
	E3	TX252	84.5	-8.8	4	TX290	116.0	12.4	0
	E4	TX99	77.5	-7.7	3	TX15	112.5	7.7	0
DTM	E1	TX308	124.0	-10.9	3	TX49	160.5	10.9	0
	E2	TX127	122.0	-16.8	5	TX39	151.0	16.8	0
	E3	TX316	131.0	-11.1	5	TX67	152.5	8.3	1
	E4	TX174	65.5	-5.2	2	TX38	147.0	5.2	0
PH	E3	TX104	56.5	-7.8	2	TX287	130.5	7.8	0
	E4	TX96	66.3	-8.0	2	TX3	116.3	8.0	0
TGW	E1	TX160	50.3	5.2	2	TX64	17.2	-5.2	0
	E2	TX231	44.7	10.4	6	TX38	17.4	-3.2	2
	E4	TX234	56.3	3.6	2	TX33	12.7	-3.6	0
GY	E1	TX150	745.0	92.3	3	TX20	93.5	-92.3	0
	E3	TX109	661.5	81.3	3	TX37	142.5	-81.3	0

Supplementary Table S5. List of contrasting genotypes selected based on breeding value and number of favourable alleles for each of the five traits considered during present study. Gen., Genotype; Fav., Favourable; BV, Breeding value

S. No.	SNP ID	Chr.	Pos.	Genomic Loc.	Len.	Score	E-val	%ID	S. N0.	Genes in 1 Mb region	Loc.	Description	GO Annotation		
													Biological process	Molecular function	Cellular component
1.	100001553	1B	520.423	No Significant hit found	-	-	-	-	-	-	-	-	-	-	-
2.	1003062	4B	60.121	4B:39758253-39758321	69	65	8.20 E-28	98.6	1.	TraesCS4B02G051200	4B: 39,757,252-39,762,477	Microtubule associated protein (MAP65/ASE1 family) (PF03999)	microtubule cytoskeleton organization (GO:0000226)	microtubule binding (GO:0008017)	N/A
									2.	TraesCS4B02G051100	4B: 39,735,632-39,735,994	Uncharacterized protein	N/A	N/A	N/A
									3.	TraesCS4B02G051300	4B: 39,901,222-39,910,565	dihydroxyacetone kinase Dak1 (PF02733)	phosphorylation (GO: 0016310	ATP binding (GO: 0005524)	N/A
									4.	TraesCS4B02G051400	4B: 40,062,565-40,066,751	SNF2 family N-terminal domain (PF00176)	N/A	ATP binding (GO: 0005524)	N/A
									5.	TraesCS4B02G051500	4B: 40,074,250-40,078,403	basic Helix-loop-helix DNA-binding domain (bHLH) (PF00010)	N/A	protein dimerization activity (GO: 0046983)	N/A
3.	1021420	2A	224.605	2A:755679307-755679375		65	8.2E-28	98.6	1.	TraesCS2A02G547200	2A: 755,479,557-755,481,086	ATP synthase (PF00306)	ATP synthesis coupled proton transport (GO: 0015986)	proton-transporting ATP synthase activity, rotational mechanism (GO: 0046933)	Plasma membrane (GO: 0005886)
									2.	TraesCS2A02G547300	2A: 755,678,995-755,680,257	Uncharacterized protein	N/A	N/A	N/A

									3.	TraesCS2A02G547400	2A: 755,692,724-755,693,351	Uncharacterized protein	N/A	N/A	N/A
									4.	TraesCS2A02G547500	2A: 755,702,713-755,706,239	S-locus glycoprotein domain (PF00954)	protein phosphorylation (GO: 0006468	protein kinase activity (GO: 0004672)	membrane (GO:0016020)
									5.	TraesCS2A02G547600	2A: 755,736,756-755,738,760	Gibberellin-Dioxygenases Gene Family (GOAx) D1: Non-haem dioxygenase N-terminal domain/PF14226; D2: Oxoglutarate/iron-dependent dioxygenase/PF03171	oxidation-reduction process (GO:0055114)	oxidoreductase activity (GO:0016491); 2. metal ion binding (GO:0046872	N/A
									6.	TraesCS2A02G547700	2A: 755,740,424-755,747,600	Peptidyl-tRNA hydrolase (PF01195)	N/A	aminoacyl-tRNA hydrolase activity (GO: 0004045)	N/A
4.	1026425	6B	75.404	6B:624402222-624402290	69	69	3.4E-30	100	1.	TraesCS6B02G355800	6B: 624,177,393-624,178,922	Gibberellin-Dioxygenases Gene Family (GOAx) D1: Non-haem dioxygenase N-terminal domain/PF14226; D2: Oxoglutarate/iron-dependent dioxygenase/PF03171	oxidation-reduction process (GO:0055114)	oxidoreductase activity (GO:0016491)	N/A
									2.	TraesCS6B02G356000	6B: 624,402,309-624,409,704	Non-haem dioxygenase N-terminal domain/PF14226; Oxoglutarate/iron-dependent dioxygenase/PF03171	oxidation-reduction process (GO:0055114)	oxidoreductase activity (GO:0016491); metal ion binding (GO:0046872)	N/A

									3.	TraesCS6B02G355900	6B: 624,311,966-624,313,762	Non-haem dioxygenase N-terminal domain/PF14226; Oxoglutarate/iron-dependent dioxygenase/PF03171	oxidation-reduction process (GO:0055114)	oxidoreductase activity (GO:0016491); metal ion binding (GO:0046872)	N/A
									4.	TraesCS6B02G356200	6B: 624,495,761-624,497,229	Non-haem dioxygenase N-terminal domain/PF14226; Oxoglutarate/iron-dependent dioxygenase/PF03171	oxidation-reduction process (GO:0055114)	oxidoreductase activity (GO:0016491); metal ion binding (GO:0046872)	N/A
5.	1026541	2B	179.515	2B:772063506-772063574	69	69	3.4E-30	100	1.	TraesCS2B02G584800	2B: 771,820,229-771,823,056	Uncharacterized protein	N/A	N/A	N/A
									2.	TraesCS2B02G585000	2B: 771,967,498-771,968,287	Uncharacterized protein	N/A	N/A	N/A
									3.	TraesCS2B02G584900	2B: 771,923,036-771,924,265	F-box domain (IPR001810)	N/A	protein binding GO:0005515	N/A
									4.	TraesCS2B02G585100	2B: 772,023,479-772,027,944	Ribosomal protein L11 methyltransferase (PrmA) (PF06325)	Methylation (GO:0032259)	methyltransferase activity (GO: 0008168)	**N/A**
									5.	TraesCS2B02G585200	2B: 772,049,586-772,053,732	DHHC palmitoyltransferase domain (PF01529)	N/A	transferase activity (GO:0016740)	membrane (GO:0016020)
									6.	TraesCS2B02G585300	2B: 772,115,726-772,121,977	OPT oligopeptide transporter protein (PF03169)	transmembrane transport (GO:0055085)	N/A	membrane (GO:0016020)
6.	1050557	5B	85.982	5B:348450682-348450747	66	62	5.1E-26	98.5	1.	TraesCS5B02G193000	5B: 348,196,663-348,198,752	Uncharacterized protein	N/A	N/A	N/A
									2.	TraesCS5B02G193100	5B: 348,448,002-348,450,302	Trehalose-phosphatase (PF02358)	trehalose biosynthetic process (GO:0005992); dephosphorylation	trehalose-phosphatase activity (GO:0004805); catalytic activity (GO:000382	N/A

													(GO:0016311)	4); hydrolase activity (GO:0016787)	
									3.	TraesCS5B02G193200	5B: 348,570,451-348,572,272	AP2/ERF gene (AP2 domain; PF00847)	transcription (GO: 0006351), regulation of transcription (GO: 0006355)	DNA-binding transcription factor activity (GO: 0003700)	Nucleus (GO:0005634)
7.	1063624	3A	75.969	3A:84182120-84182188	69	69	3.4E-30	100	1.	TraesCS3A02G116200	3A: 83,977,019-83,983,489	WRKY protein domain (PF03106)	regulation of transcription (GO: 0006355)	DNA-binding transcription factor activity (GO: 0003700)	Nucleus (GO:0005634)
									2.	TraesCS3A02G116300	3A: 84,184,377-84,192,886	Uncharacterized protein	N/A	N/A	N/A
8.	1065193	3A	88.133	3A:568445450-568445518	69	69	3.4E-30	100	1.	TraesCS3A02G323500	3A: 568,094,977-568,098,215	Bulb-type lectin domain (PF01453), S-locus glycoprotein domain (PF00954), PAN/Apple domain (PF08276), Serine-threonine/tyrosine-protein kinase, catalytic domain (PF07714)	protein phosphorylation (GO: 0006468)	protein serine/threonine kinase activity (GO: 0004674)	membrane (GO:0016020)
									2.	TraesCS3A02G323600	3A: 568,146,930-568,150,432	Uncharacterized protein	N/A	N/A	N/A
									3.	TraesCS3A02G323900	3A: 568,424,166-568,425,588	Uncharacterized protein	N/A	N/A	N/A
									4.	TraesCS3A02G324000	568,444,269-568,446,437	Uncharacterized protein	N/A	protein dimerization activity (GO:0046983)	N/A
									5.	TraesCS3A02G324100	3A: 568,663,686-568,666,242	Uncharacterized protein	N/A	N/A	N/A

									6.	TraesCS3A02G324200	3A: 568,971,914-568,973,639	Uncharacterized protein	N/A	N/A	N/A
9.	1069547	3B	253.745	No Significant hit found	-	-	-	-	-	-	-	-	-	-	-
10.	1073429	6A	88.944	6A:116943406-116943474	69	69	3.4E-30	100	1.	TraesCS6A02G142000	6A: 116,812,872-116,813,579	Uncharacterized protein	N/A	protein dimerization activity (GO:0046983)	N/A
									2.	TraesCS6A02G142100	6A: 116,907,128-116,911,553	Synaptotagmin-like mitochondrial-lipid-binding domain (IPR031468)	Lipid transport (GO: 0006869	Lipid binding (GO: 0008289)	Membrane (GO:0016020)
									3.	TraesCS6A02G142200	6A: 116,911,948-116,913,052	Patatin-like phospholipase (PF01734)	lipid metabolic process (GO:0006629)	N/A	N/A
									4.	TraesCS6A02G142300	6A: 117,003,771-117,005,477	Uncharacterized protein	N/A	N/A	N/A
11.	1080969	6B	83.710	No Significant hit found	-	-	-	-	-	-	-	-	-	-	-
12.	1083327	2A	237.185	No Significant hit found	-	-	-	-	-	-	-	-	-	-	-
13.	1090844	5A	177.183	5A:595082910-595082978	69	69	3.4E-30	100	1.	TraesCS5A02G401800	5A: 594,770,622-594,772,285	Dof domain, zinc finger (PF02701)	regulation of transcription (GO: 0006355)	DNA binding (GO:0003677)	Nucleus (GO:0005634)
									2.	TraesCS5A02G401900	5A: 594,953,137-594,957,074	Uncharacterized protein	N/A	N/A	N/A
									3.	TraesCS5A02G402000	5A: 594,957,998-594,960,865	Uncharacterized protein	N/A	N/A	N/A
									4.	TraesCS5A02G402100	5A: 594,961,396-594,964,746	Peptidase_S26 (PF 10502)	proteolysis (GO: 0006508)	serine-type peptidase activity (GO: 0008236)	Membrane (GO:0016020)

									5.	TraesCS5A02G402200	5A: 594,965,774-594,970,482	Nucleoporin p58/p45 (IPR024882)	nucleocytoplasmic transport (GO:0006913)	N/A	Nuclear pore (GO:0005643)
									6.	TraesCS5A02G402300	5A; 595,037,147-595,038,924	Uncharacterized protein	N/A	N/A	N/A
									7.	TraesCS5A02G402400	5A: 595,037,573-595,037,749	Uncharacterized protein	N/A	N/A	N/A
									8.	TraesCS5A02G402500	5A: 595,143,767-595,147,549	Uncharacterized protein	N/A	N/A	N/A
14.	1091457	5D	100.955	5D:391278717-391278776	60	60	7.9E-25	100	1.	TraesCS5D02G294100	5D: 391,039,172-391,046,409	HORMA domain (named after the Hop1p, Rev7p and MAD2 proteins) (PF02301)	synapsis (GO:0007129)	N/A	Nuclear chromosome (GO:0000228)
									2.	TraesCS5D02G294200	5D: 391,048,851-391,053,786	Uncharacterized protein	N/A	N/A	N/A
									3.	TraesCS5D02G294300	5D: 391,328,178-391,329,395	Uncharacterized protein	N/A	N/A	N/A
									4.	TraesCS5D02G294400	5D: 391,372,552-391,378,851	Squamosa promoter binding protein domain (SBP;PF03110)	N/A	DNA binding (GO:0003677)	Nucleus (GO:0005634)
15.	1093068	6B	82.603	6B:657097981-657098049	69	69	3.4E-30	100	1.	TraesCS6B02G382000	6B: 656,996,783-657,001,563	Uncharacterized protein	N/A	N/A	N/A
									2.	TraesCS6B02G382100	6B: 657,089,245-657,091,238	Uncharacterized protein	N/A	N/A	N/A
									3.	TraesCS6B02G382200	6B: 657,092,121-657,092,279	Uncharacterized protein	N/A	N/A	N/A
16.	1093542	7A	157.225	7A:529453928-529453996	69	65	8.2E-28	98.6	1.	TraesCS7A02G358400	7A: 529,325,637-529,332,480	HSP60 (TCP-1/cpn60 chaperonin family; PF00118)	Protein folding (GO:0006457)	Nucleotide binding (GO: 0000166); unfolded protein binding (GO:0051082)	Cytoplasm (GO:0005737)
17.	1094628	4A	177.335	4A:667344925-667344993	69	65	8.2E-28	98.6	1..	TraesCS4A02G389600	4A: 666,980,143-666,983,528	NB-ARC domain (PF00931)	N/A	ADP binding (GO: 0043531)	N/A

									2.	TraesCS4A02G389700	4A: 666,983,563-666,984,354	Uncharacterized protein	N/A	N/A	N/A
									3.	TraesCS4A02G389800	4A: 667,062,760-667,063,644	Uncharacterized protein	N/A	N/A	N/A
									4.	TraesCS4A02G389900	4A: 667,174,383-667,174,854	D1: Cysteine-rich transmembrane CYSTM domain/PF12734	N/A	N/A	N/A
									5.	TraesCS4A02G390000	4A: 667,225,463-667,226,800	Uncharacterized protein	N/A	N/A	N/A
									6.	TraesCS4A02G390100	4A: 667,330,919-667,331,413	D1: Cysteine-rich transmembrane CYSTM domain/PF12734	N/A	N/A	N/A
									7.	TraesCS4A02G390200	4A: 667,344,888-667,346,497	D1: Cysteine-rich transmembrane CYSTM domain/PF12734	N/A	N/A	N/A
									8.	TraesCS4A02G390300	4A: 667,349,594-667,351,134	Uncharacterized protein	N/A	N/A	N/A
									9.	TraesCS4A02G390400	4A: 667,352,002-667,355,329	Ribosomal protein L11 methyltransferase (PrmA) (PF06325)	Methylation (GO:0032259); protein methylation (GO:0006479)	methyltransferase activity (GO: 0008168)	N/A
									10.	TraesCS4A02G390500	4A: 667,379,945-667,381,624	mitochondrial transcription termination factor (mTERF) (PF02536)	regulation of transcription (GO: 0006355)	double-stranded DNA binding (GO: 003690)	N/A
									11.	TraesCS4A02G390600	4A: 667,425,334-667,426,494	Uncharacterized protein	N/A	N/A	N/A
18.	1096003	1B	64.812	No Significant hit found	-	-	-	-	-	-	-	-	-	-	-
19.	1103347	1A	247.886	No Significant hit found	-	-	-	-	-	-	-	-	-	-	-
20.	1110448	4D	123.880	4D:477016515-477016583	69	69	3.4E-30	100	1.	TraesCS4D02G308900	4D: 477,006,536-477,008,165	Cyclin-dependent kinase regulatory	Regulation of cyclin-dependent	Regulation of cyclin-dependent	N/A

												subunit family (PF01111)	protein serine/threonine kinase activity (GO:0000079); Cell cycle (GO:0007049); Phosphorylation (GO:0016310); cell division (GO:0051301)	protein serine/threonine kinase activity (GO:0000079)	
									2.	TraesCS4D02G309000	4D: 477,012,133-477,016,966	Rieske [2Fe-2S] iron-sulphur domain/PF00355; Pheophorbide a oxygenase/PF08417	cell death (GO:0008219); flower development (GO:0009908); fruit development (GO:0010154); chlorophyll catabolic process (GO:0015996); oxidation-reduction process (GO:0055114)	oxidoreductase activity (GO:0016491); chlorophyllide a oxygenase [overall] activity (GO:0010277); pheophorbide a oxygenase activity (GO:0032441)	chloroplast (GO:0009507)
									3.	TraesCS4D02G309100	4D: 477,030,201-477,030,680	Uncharacterized protein	N/A	N/A	N/A
									4.	TraesCS4D02G309200	4D: 477,034,398-477,043,019	Pentatricopeptide repeat (PPR_3) (PF13812)	N/A	Protein binding (GO:0005515)	N/A
									5.	TraesCS4D02G309300	4D: 477,087,511-477,089,567	Enoyl-(Acyl carrier protein) reductase (PF13561)	oxidation-reduction process (GO:0055114)	1. oxidoreductase activity (GO:0016491);	N/A

									6.	TraesCS4D02G309400	4D: 477,098,674-477,101,510	Uncharacterized protein	N/A	N/A	N/A
21.	1113894	1B	91.804	1B:38599717-38599781	65	61	2E-25	98.5	1.	TraesCS1B02G055800	1B: 38,832,030-38,835,862	SET, SAD_SRA (PF02182);D2 Pre-SET (PF05033); D3, SET (PF00856)	Histone methylation (GO:0016571); Histone lysine methylation (GO:0034968)	protein binding (GO:0005515); 2. Zinc ion binding (GO:0008270); Histone-lysine N-methyltransferase activity (GO:0018024)	Nucleus (GO:0005634); Chromosome (GO:0005694)
									2.	TraesCS1B02G055900	1B: 38,906,993-38,911,737	Uncharacterized protein	N/A	N/A	N/A
22.	1125402	2A	231.786	No Significant hit found	-	-	-	-	-	-	-	-	-	-	-
23.	1126379	4A	250.448	4A:743768873-743768931	59	55	7.6E-22	98.3	1.	TraesCS4A02G497700	4A: 743,504,907-743,505,176	Uncharacterized protein	N/A	N/A	N/A
									2.	TraesCS4A02G497800	4A: 743,552,934-743,565,036	Protein kinase domain (PF00069)	Protein phosphorylation (GO: 0006468)	Protein kinase activity (GO:0004672); ATP binding (GO:0005524); ADP binding (GO:0043531)	N/A
									3.	TraesCS4A02G497900	4A: 743,674,850-743,676,472	Protein kinase domain (PF00069)	Protein phosphorylation (GO: 0006468)	protein kinase activity (GO:0004672); ATP binding (GO:0005524)	N/A
									4.	TraesCS4A02G498000	4A: 743,680,277-743,680,807	Uncharacterized protein	N/A	N/A	N/A
									5.	TraesCS4A02G498100	4A: 743,748,235-743,749,019	Late embryogenesis abundant protein	N/A	N/A	N/A

												(LEA_2) (PF03168)			
24.	1127414	3B	94.818	3B:95751799-95751867	69	65	8.2E-28	98.6	1.	TraesCS3B02G123600	3B: 95,459,819-95,462,522	Zinc finger C2H2-type (IPR013087)	N/A	nucleic acid binding (GO:0003676)	N/A
25.	1129498	6D	9.626	6D:7115034-7115102	69	69	3.4E-30	100	1.	TraesCS6D02G016500	6D: 6,963,495-6,970,978	Cenp-O kinetochore centromere component (PF09496)	Centromere complex assembly (GO: 0034508)	N/A	Kinetochore (GO: 0000776)
									2.	TraesCS6D02G016600	6D: 6,977,348-6,980,077	Uncharacterized protein	N/A	N/A	N/A
									3.	TraesCS6D02G016700	6D: 6,982,048-6,983,620	Uncharacterized protein	N/A	N/A	N/A
									4.	TraesCS6D02G016800	6D: 7,032,180-7,035,207	Leucine rich repeat N-terminal domain (PF08263)	N/A	Protein binding (GO:0005515)	Membrane (GO:0016020)
26.	1130017	6D	186.636	No Significant hit found	-	-	-	-	-	-	-	-	-	-	-
27.	1133812	5A	96.128	No Significant hit found	-	-	-	-	-	-	-	-	-	-	-
28.	1134753	3B	264.027	3B:799744734-799744802	69	69	3.4E-30	100	1.	TraesCS3B02G567900	3B: 799,448,122-799,449,444	Uncharacterized protein	N/A	N/A	N/A
									2.	TraesCS3B02G568000	3B: 799,521,193-799,524,789	Ubiquitin (PF00240)	N/A	Protein binding (GO:0005515)	N/A
									3.	TraesCS3B02G568100	3B: 799,810,448-799,812,760	Uncharacterized protein	N/A	N/A	N/A
29.	1167608	5B	79.052	5B:472663029-472663097	69	69	3.4E-30	100	1.	TraesCS5B02G286400	5B: 472,274,786-472,275,977	Uncharacterized protein	N/A	N/A	N/A
									2.	TraesCS5B02G286500	5B: 472,676,123-472,676,473	Uncharacterized protein	N/A	N/A	N/A
30.	1202201	3B	117.678	3B:506732534-506732602	69	69	3.4E-30	100	1.	TraesCS3B02G314800	3B: 506,619,433-506,620,903	Transcriptional repressor, ovate (PF04844)	Negative regulation of transcription, DNA-templated	N/A	N/A

													(GO: 00445892)		
									2.	TraesCS3B02G314900	3B: 506,623,778-506,631,741	50S ribosome-binding GTPase (PF01926)	N/A	GTP binding (GO: 0005525)	N/A
									3.	TraesCS3B02G315000	3B: 506,734,252-506,734,449	translocase of the outer membrane (TOM); Tom7 (PF08038)	Protein import into mitochondrial matrix (GO: 0030150)	N/A	Mitochondrial outer membrane translocase complex (GO: 0005742)
31.	1217183	2B	203.502	No Significant hit found	-	-	-	-	-	-	-	-	-	-	-
32.	1228079	2D	289.675	2D:648639327-648639395	69	53	1.2E-20	94.2	1.	TraesCS2D02G595000	2D: 648,466,884-648,471,442	Leucine rich repeat N-terminal domain (PF08263); Tyrosine kinase (PF07714)	Protein phosphorylation (GO: 0006468)	Nucleotide binding (GO: 0000166); protein kinase activity (GO:0004672); Protein serine/threonine kinase activity (GO: 0004674); ATP binding (GO:0005524)	Membrane (GO:0016020)
									2.	TraesCS2D02G595100	2D: 648,473,712-648,476,886	NUDIX domain (PF00293)	Nucleoside phosphate metabolic process (GO: 0006753)	Guanosine-3',5'-bis(diphosphate) 3'-diphosphatase activity (GO:0008893); Hydrolase activity (GO:0016787); Bis(5'-adenosyl)-pentaphosphatase activity (GO:0034432)	chloroplast (GO:0009507)
									3.	TraesCS2D02G595200	2D: 648,570,416-648,573,833	Uncharacterized protein	N/A	N/A	N/A

									4.	TraesCS2D02G595300	2D: 648,576,485-648,578,874	Rx_N terminal domain (PF18052); NB-ARC domain(PF00931)	N/A	ADP binding (GO:0043531)	N/A
									5.	TraesCS2D02G595400	2D: 648,595,034-648,595,774	Uncharacterized protein	N/A	N/A	N/A
									6.	TraesCS2D02G595500	2D: 648,634,921-648,639,221	Amino acid transporter, transmembrane/PF01490	Amino acid transmembrane transport (GO:0003333)	Amino acid transmembrane transporter activity (GO:0015171)	Membrane (GO:0016020)
33.	1270494	4B	76.346	4B:562867052-562867120	69	65	8.2E-28	98.6	1.	TraesCS4B02G279500	4B: 562,628,791-562,629,180	Uncharacterized protein	N/A	N/A	N/A
									2.	TraesCS4B02G279600	4B: 562,697,710-562,698,044	Uncharacterized protein	N/A	N/A	N/A
									3.	TraesCS4B02G279700	4B: 562,698,914-562,701,787	Mitochondrial transcription termination factor (mTERF) (PF02536)	Regulation of transcription, DNA-templated (GO:0006355)	Double-stranded DNA binding (GO:0003690)	N/A
									4.	TraesCS4B02G279800	4B: 562,704,755-562,707,829	pfkB family carbohydrate kinase (PF00294)	N/A	N/A	N/A
									5.	TraesCS4B02G279900	4B: 562,709,125-562,712,214	Zein-binding (PF04576)	N/A	myosin binding (GO:0017022)	Membrane (GO:0016020)
34.	3023142	5B	171.943	5B:588883282-588883319	38	38	1.1E-11	100	1.	TraesCS5B02G414300	5B: 588,778,560-588,780,838	NAD dependent epimerase/dehydratase family (PF01370)	N/A	catalytic activity (GO:0003824); coenzyme binding (GO:0050662)	N/A
									2.	TraesCS5B02G414400	5B: 588,831,208-588,832,584	Cytochrome P450 (PF00067)	Oxidation-reduction process (GO:0055114)	Iron ion binding (GO:0005506); Oxidoreductase activity,	N/A

														acting on paired donors, with incorporation or reduction of molecular oxygen (GO:0016705)	
									3.	TraesCS5B02G414500	5B: 588,832,694-588,834,933	NAD dependent epimerase/dehydratase family (PF01370)	N/A	catalytic activity (GO:0003824); coenzyme binding (GO:0050662)	N/A
35.	3028936	4B	80.804	No Significant hit found	-	-	-	-	-	-	-	-	-	-	-
36.	3030495	3A	21.260	No Significant hit found	-	-	-	-	-	-	-	-	-	-	-
37.	977937	2A	150.126	2A:677497865-677497929	65	61	2E-25	98.5	1.	TraesCS2A02G422500	2A: 677,485,523-677,488,079	Uncharacterized protein	N/A	N/A	N/A
									2.	TraesCS2A02G422600	2A: 677,524,804-677,530,472	K+ potassium transporter (PF02705)	Potassium ion transmembrane transport (GO:0071805)	Potassium ion transmembrane transporter activity (GO:0015079)	Membrane (GO:0016020)
									3.	TraesCS2A02G422700	2A: 677,530,470-677,532,528	GRF zinc finger (PF06839)	N/A	Zinc ion binding (GO:0008270)	N/A
38.	985312	4B	57.883	4B:37529691-37529759	69	69	3.4E-30	100	1.	TraesCS4B02G049000	4B: 37,296,414-37,297,430	Protein kinase domain (PF00069)	Protein phosphorylation (GO: 0006468)	protein kinase activity (GO:0004672); ATP binding (GO:0005524)	N/A
									2.	TraesCS4B02G049100	4B: 37,315,983-37,320,613	RNA recognition motif (PF00076)	N/A	Nucleic acid binding (GO:0003676); RNA	N/A

														binding (GO:0003723)	
									3.	TraesCS4B02G049200	4B: 37,334,240-37,335,061	Uncharacterized protein	N/A	N/A	N/A
									4.	TraesCS4B02G049300	4B: 37,689,759-37,694,794	Uncharacterized protein	N/A	N/A	N/A
39.	985813	6B	69.052	6B:649814739-649814807	69	65	8.2E-28	98.6	1.	TraesCS6B02G375300	6B: 649,826,817-649,827,104	Uncharacterized protein	N/A	N/A	N/A
									2.	TraesCS6B02G375400	6B: 649,831,108-649,834,547	AP2/ERF gene (AP2 domain;PF00847)	transcription (GO: 0006351), regulation of transcription (GO: 0006355)	DNA-binding transcription factor activity (GO: 0003700)	Nucleus (GO:0005634)
									3.	TraesCS6B02G375500	6B: 649,909,920-649,920,739	Uncharacterized protein	N/A	N/A	N/A
40.	986158	1B	384.128	1B:641202994-641203062	69	69	3.4E-30	100	1.	TraesCS1B02G416000	1B: 641,022,992-641,035,971	C1 domain (also known as phorbol esters/diacylglycerol binding domain) (PF03107)	Intracellular signal transduction (GO:0035556)	N/A	N/A
									2.	TraesCS1B02G416100	1B: 641,037,521-641,038,069	Uncharacterized protein	N/A	N/A	N/A
									3.	TraesCS1B02G416200	1B: 641,196,834-641,203,984	Rx_N terminal domain (PF18052) ; NB-ARC domain(PF00931)	N/A	Protein binding (GO:0005515); ADP binding (GO:0043531)	N/A
									4.	TraesCS1B02G416300	1B: 641,212,975-641,213,405	Uncharacterized protein	N/A	N/A	N/A
41.	992291	7B	114.753	7B:606562054-606562122	69	65	8.2E-28	98.6	1.	TraesCS7B02G349500	7B: 606,353,537-606,356,738	Uncharacterized protein	N/A	N/A	N/A
									2.	TraesCS7B02G349600	7B: 606,398,372-606,398,923	Uncharacterized protein	N/A	N/A	N/A

									3.	TraesCS7B02G349700	7B: 606,556,159-606,563,497	Non-haem dioxygenase N-terminal domain/PF14226; Oxoglutarate/iron-dependent dioxygenase/PF03171	oxidation-reduction process (GO:0055114)	oxidoreductase activity (GO:0016491); metal ion binding (GO:0046872)	N/A
									4.	TraesCS7B02G349800	7B: 606,644,011-606,646,843	Cytochrome P450 (PF00067)	oxidation-reduction process (GO:0055114)	oxidoreductase activity (GO:0016491); metal ion binding (GO:0046872), heme binding (GO: 0020037)	membrane (GO:0016020)

Supplementary Table S6. Details of 121 genes associated with significant MTAs identified using Ensembl Plant database (a window of 1 Mb was used for identification of CGs). Details of putative proteins identified from Ensembl wheat also given. # Pfam database IDs. Chr., chromosome. Pos., position. Loc., location, Len., length.

Environment	SNP	Major allele	Frequency	Mean GY	Minor allele	Frequency	Mean GY	-log (p)
E1	SNP_7093	T	0.96	411.83	G	0.04	222.79	5.4
	SNP_6731	G	0.97	410.63	C	0.03	252.50	3.4
	SNP_5523	T	0.97	408.80	G	0.03	243.75	3.6
	SNP_6749	C	0.97	402.25	G	0.03	563.75	3.5
	SNP_3997	T	0.96	410.33	A	0.04	251.12	3.9
E2	SNP_7093	T	0.96	224.08	G	0.04	114.17	4.8
	SNP_6261	G	0.96	223.69	A	0.04	114.77	4.4
	SNP_6731	G	0.97	223.77	C	0.03	125.00	3.4
	SNP_4491	T	0.96	223.80	G	0.04	125.00	3.7
	SNP_3997	T	0.96	223.15	A	0.04	127.71	3.8

Supplementary Table S7. List of SNPs having rare variant (minor allele frequency <0.05) showing significant difference (using t-test) for grain yield (GY) between genotypes with contrasting SNP alleles.

Taxon ID	Pedigree	Country of Origin
TX1	ZAMBESI	ZIMBABWE
TX2	CAESIUM 31	USSR
TX3	63-R-3001	UNITED STATES
TX4	PETTERSON ML68-4	IRAN
TX5	LC/4/GB*3/3/KENYA C6042//WAGGA 13/MS-A	
TX6	FRTR/4/RT//SPIJK/SHD/3/AKA/5/RED EGYPTIAN(PI-170925)/KENYA BF4-3B.10.V.1/6/H/4/3*HRC/FIFE//IM/3/HRC/FIFE//KRYMKI/13/DRM/MI//KRYMKI/12/KRYMKI/7/FIFE/2*RIBA/6/FIFE/5/FIFE//FIFE/FIFE/4/FIFE/3/FIFE/FIFE//INDIAN G/8/DIEHL/MI/3/PP-AUS//FIFE/ETAWAH/9/ORO/10/DIEHL/MI/3/PP-AUS//FIFE/ETAWAH/11/ORO/8/KRYMKI/7/FIFE/2*RIBA/6/FIFE/5/FIFE//FIFE/FIFE/4/FIFE	MEXICO
TX7	D-12	N/A
TX8	GRAY JD253	AFGHANISTAN
TX9	GRAY JD416	AFGHANISTAN
TX10	GRAY JD629	AFGHANISTAN
TX11	GRAY JD738	AFGHANISTAN
TX12	GRAY JD757	AFGHANISTAN
TX13	GRAY JD893	AFGHANISTAN
TX14	GRAY JD930	AFGHANISTAN
TX15	GRAY JD1004	AFGHANISTAN
TX16	GRAY JD1024	AFGHANISTAN
TX17	GRAY JD1032	AFGHANISTAN
TX18	GRAY JD1102	AFGHANISTAN
TX19	GRAY JD1196	AFGHANISTAN
TX20	GRAY JD1278	AFGHANISTAN
TX21	GRAY JD1447	AFGHANISTAN
TX22	C-38-1	PHILIPPINES
TX23	C-40-1	PHILIPPINES
TX24	MOSKOVSKAYA 21 VIR 48760	RUSSIA
TX25	W-33-A	PAKISTAN
TX26	W-36	PAKISTAN
TX27	W-37	PAKISTAN
TX28	W-39	N/A
TX29	W-63	PAKISTAN
TX30	W-65	PAKISTAN
TX31	W-67	PAKISTAN

TX32	W-73	PAKISTAN
TX33	W-78-A	PAKISTAN
TX34	W-79	PAKISTAN
TX35	W-83	PAKISTAN
TX36	W-84	N/A
TX37	W-85	PAKISTAN
TX38	W86	N/A
TX39	W-88	N/A
TX40	W-91	PAKISTAN
TX41	GONG JIAO 279	CHINA
TX42	BW110	SAUDI ARABIA
TX43	QITAI-CHUN-4-ST-119	CHINA
TX44	TURPAN SHAN YUEH H C M	CHINA
TX45	TOKSUN SPRING 2 ST-122	CHINA
TX46	WANNIAN 2	CHINA
TX47	HONG DUAN MANG	CHINA
TX48	CItr 2346	N/A
TX49	CItr 2397	RUSSIA
TX50	YANTAGBAY	CHINA
TX51	LOROS	N/A
TX52	CItr 4309	IRAN
TX53	CItr 4315	IRAN
TX54	CItr 4901	INDIA
TX55	CI 5088	CHINA
TX56	JENKIN	N/A
TX57	BIHAR-51	N/A
TX58	BIHAR-59	INDIA
TX59	BIHAR-66	N/A
TX60	ALI-BEN-MAKLOUL	ALGERIA
TX61	PERFUME	IRAN
TX62	WILBUR	N/A
TX63	KLEIN FAVORITO	N/A
TX64	BARBELLA/SANTA MARTHA	PORTUGAL
TX65	LOB/RBO	PORTUGAL
TX66	CANDEAL:AE	N/A
TX67	CItr 7089	RUSSIA
TX68	CItr 7289	INDIA
TX69	MARGARITOVO	RUSSIA
TX70	HRC/FIFE//GEHUN/FRASER	N/A
TX71	DORSETT-PH-3892	CHINA
TX72	CItr 8327	CHINA
TX73	DORSETT-PH-2140	N/A
TX74	GRANDE-DEL-MONTE	N/A
TX75	ROJO BARBON	MEXICO
TX76	CItr 8517	RUSSIA
TX77	LOVE-HH-129	CHINA
TX78	DORSETT-PH-6955	CHINA
TX79	DORSETT-PH-6993	CHINA
TX80	DORSETT-PH-7017	CHINA
TX81	DORSETT-PH-7053	CHINA
TX82	DORSETT-PH-6927	CHINA
TX83	DORSETT-PH-7150	CHINA
TX84	200, PI70709	IRAQ
TX85	LOVE-HH-G23B	CHINA
TX86	LOVE-HH-CHINA 66	CHINA
TX87	II12107-3R-6M-2R	MEXICO
TX88	CItr 14998	NEPAL
TX89	CItr 15308	AFGHANISTAN
TX90	RUPERT J CAR 1035 (27035)	CHILE
TX91	KING-HING-1	CHINA
TX92	AKBUGDAY	N/A
TX93	AKBASAK	TURKEY
TX94	PAVON/19/CAR422/18/KT/6/H/4/3*HRC/FIFE//IM/3/HRC/FIFE//KRYMKI/5/FA/7/H/4/3*HRC/FIFE//IM/3/HRC/FIFE//KRYMKI/5/FA/6/KT/8/KT/5/FRTR/4/RT//SPIJK/SHD/3/AKA/6/H/4/3*HRC/FIFE//IM/3/HRC/FIFE//KRYMKI/5/FA/9/SWD/T.TIMOPHEEVII//RED EGYPTIAN(PI-170925)/KENYA BF4-3B.10.V.1/4/GB/KENIA RF 324/3/BTA/CHI//AMO25E/PELON 33C/16/LR64/15/FRTR/4/RT//SPIJK/SHD/3/AKA/5/RED EGYPTIAN(PI-170925)/KENYA BF4-	MEXICO

	3B.10.V.1/6/H/4/3*HRC/FIFE//IM/3/HRC/FIFE//KRYMKI/13/DRM/MI//KRYMKI/12/KRYMKI/7/FIFE/2*RIBA/6/FIFE/5/FIFE//FIFE/FIFE/4/FIFE/3/FIFE/FIFE//INDIAN G/8/DIEHL/MI/3/PP-AUS//FIFE/ETAWAH/9/ORO/10/DIEHL/MI/3/PP-AUS//FIFE/ETAWAH/11/ORO/8/KRYMKI/7/FIFE/2*RIBA/6/FIFE/5/FIFE//	
TX95	BABAX/AMAD//BABAX	MEXICO
TX96	KT/6/H/4/3*HRC/FIFE//IM/3/HRC/FIFE//KRYMKI/5/FA/7/H/4/3*HRC/FIFE//IM/3/HRC/FIFE//KRYMKI/5/FA/6/KT/8/KT/5/FRTR/4/RT//SPIJK/SHD/3/AKA/6/H/4/3*HRC/FIFE//IM/3/HRC/FIFE//KRYMKI/5/FA/9/SWD/T.TIMOPHEEVII//RED EGYPTIAN(PI-170925)/KENYA BF4-3B.10.V.1/4/GB/KENIA RF 324/3/BTA/CHI//AMO25E/PELON 33C/16/LR64/15/FRTR/4/RT//SPIJK/SHD/3/AKA/5/RED EGYPTIAN(PI-170925)/KENYA BF4-3B.10.V.1/6/H/4/3*HRC/FIFE//IM/3/HRC/FIFE//KRYMKI/13/DRM/MI//KRYMKI/12/KRYMKI/7/FIFE/2*RIBA/6/FIFE/5/FIFE//FIFE/FIFE/4/FIFE/3/FIFE/FIFE//INDIAN G/8/DIEHL/MI/3/PP-AUS//FIFE/ETAWAH/9/ORO/10/DIEHL/MI/3/PP-AUS//FIFE/ETAWAH/11/ORO/8/KRYMKI/7/FIFE/2*RIBA/6/FIFE/5/FIFE//FIFE/FIFE/4/FIFE/3/FIFE/FIFE//INDIAN G/14/SWD/T.TIMOPHEEVII//RED EGYPTIAN(PI-170925)/KENYA BF4-3B.10.V.1/4/GB/KENIA RF 324/3/BTA/CHI//AMO25E/PELON 33C/17/LR64/15/H/4/3*HRC/FIFE//IM/3/HRC/FIFE//KRYMKI/5/FA/6/KT/7/FRTR/4/RT//SPIJK/SHD/3/AKA/13/DRM/MI//KRYMKI/12/KRYMKI/7/FIFE/2*RIBA/6/FIFE/5/FIFE//FIFE/FIFE/4/FIFE/3/FIFE/FIFE//INDIAN G/8/DIEHL/MI/3/PP-AUS//FIFE/ETAWAH/9/ORO/10/DIEHL/MI/3/PP-AUS//FIFE/ETAWAH/11/ORO/8/KRYMKI/7/FIFE/2*RIBA/6/FIFE/5/FIFE//FIFE/FIFE/4/FIFE/3/FIFE/FIFE//INDIAN G/14/2*H/4/3*HRC/FIFE//IM/3/HRC/FIFE//KRYMKI/5/FA/6/EGYPT NA101/7/SWD/T.TIMOPHEEVII/18/BJY/19/VEE	MEXICO
TX97	KAUZ//GA/AOS/3/KAUZ	MEXICO
TX98	BJY/18/KT/6/H/4/3*HRC/FIFE//IM/3/HRC/FIFE//KRYMKI/5/FA/7/H/4/3*HRC/FIFE//IM/3/HRC/FIFE//KRYMKI/5/FA/6/KT/8/KT/5/FRTR/4/RT//SPIJK/SHD/3/AKA/6/H/4/3*HRC/FIFE//IM/3/HRC/FIFE//KRYMKI/5/FA/9/SWD/T.TIMOPHEEVII//RED EGYPTIAN(PI-170925)/KENYA BF4-3B.10.V.1/4/GB/KENIA RF 324/3/BTA/CHI//AMO25E/PELON 33C/16/LR64/15/FRTR/4/RT//SPIJK/SHD/3/AKA/5/RED EGYPTIAN(PI-170925)/KENYA BF4-3B.10.V.1/6/H/4/3*HRC/FIFE//IM/3/HRC/FIFE//KRYMKI/13/DRM/MI//KRYMKI/12/KRYMKI/7/FIFE/2*RIBA/6/FIFE/5/FIFE//FIFE/FIFE/4/FIFE/3/FIFE/FIFE//INDIAN G/8/DIEHL/MI/3/PP-AUS//FIFE/ETAWAH/9/ORO/10/DIEHL/MI/3/PP-AUS//FIFE/ETAWAH/11/ORO/8/KRYMKI/7/FIFE/2*RIBA/6/FIFE/5/FIFE//FIFE/FIFE/4/FIFE/3/FIFE/FIFE//INDIAN G/14/SWD/T.TIMOPHEEVII//RED EGYPTIAN(PI-170925)/KENYA BF4-3B.10.V.1/4/GB/KENIA RF 324/3/BTA/CHI//AMO25E/PELON 33C/17/LR64/15/H/4/3*HRC/FIFE//IM/3/HRC/FIFE//KRYMKI/5/FA/6/KT/7/FRTR/4/RT//SPIJK/SHD/3/AKA/13/DRM/MI//KRYMKI/12/KRYMKI/7/FIFE/2*RIBA/6/FIFE/5/FIFE//FIFE/FIFE/4/FIFE/3/FIFE/FIFE//INDIAN G/8/DIEHL/MI/3/PP-AUS//FIFE/ETAWAH/9/ORO/10/DIEHL/MI/3/PP-AUS//FIFE/ETAWAH/11/ORO/8/KRYMKI/7/FIFE/2*RIBA/6/FIFE/5/FIFE//FIFE/FIFE/4/FIFE/3/FIFE/FIFE//INDIAN G/14/2*H/4/3*HRC/FIFE//IM/3/HRC/FIFE//KRYMKI/5/FA/6/EGYPT NA101/7/SWD/T.TIMOPHEEVII/19/VEE*2/PRL/20/KAUZ	MEXICO
TX99	KAUZ*2/FCT	MEXICO
TX100	SW89.3064/STAR	MEXICO
TX101	OTUS//MUNIA/KAUZ	MEXICO
TX102	WEAVER/LOTUS//2*WEAVER	MEXICO
TX103	KAUZ//BOW/NKT	MEXICO
TX104	RDWG/MILAN	MEXICO
TX105	SW89.5181/KAUZ	MEXICO
TX106	CMH82A.1294/2*KAUZ//MUNIA/CHTO/3/MILAN	MEXICO
TX107	CHIBIA/PASTOR//CHIBIA	MEXICO
TX108	FRTR/4/RT//SPIJK/SHD/3/AKA/5/HRC/FIFE//IM/3/HRC/FIFE//KRYMKI/4/BTA/AMO44D//BTA/CHI/15/H/4/3*HRC/FIFE//IM/3/HRC/FIFE//KRYMKI/5/FA/6/KT/7/FRTR/4/RT//SPIJK/SHD/3/AKA/13/DRM/MI//KRYMKI/12/KRYMKI/7/FIFE/2*RIBA/6/FIFE/5/FIFE//FIFE/FIFE/4/FIFE/3/FIFE/FIFE//INDIAN G/8/DIEHL/MI/3/PP-AUS//FIFE/ETAWAH/9/ORO/10/DIEHL/MI/3/PP-AUS//FIFE/ETAWAH/11/ORO/8/KRYMKI/7/FIFE/2*RIBA/6/FIFE	MEXICO
TX109	VEE/PJN//2*TUI/3/WH576	MEXICO
TX110	MUNIA/3/RUFF/FGO//BIT/4/PASTOR	MEXICO
TX111	D65152/D6148//R143/3/ENTE/STR/4/AEGILOPS SQUARROSA (TAUS)/5/WEAVER/6/IRENA	MEXICO
TX112	ATTILA/19/CAR422/18/KT/6/H/4/3*HRC/FIFE//IM/3/HRC/FIFE//KRYMKI/5/FA/7/H/4/3*HRC/FIFE//IM/3/HRC/FIFE//KRYMKI/5/FA/6/KT/8/KT/5/FRTR/4/RT//SPIJK/SHD/3/AKA/6/H/4/3*HRC/FIFE//IM/3/HRC/FIFE//KRYMKI/5/FA/9/SWD/T.TIMOPHEEVII//RED EGYPTIAN(PI-170925)/KENYA BF4-3B.10.V.1/4/GB/KENIA RF 324/3/BTA/CHI//AMO25E/PELON 33C/16/LR64/15/FRTR/4/RT//SPIJK/SHD/3/AKA/5/RED EGYPTIAN(PI-170925)/KENYA BF4-3B.10.V.1/6/H/4/3*HRC/FIFE//IM/3/HRC/FIFE//KRYMKI/13/DRM/MI//KRYMKI/12/KRYMKI/7/FIFE/2*RIBA/6/FIFE/5/FIFE//FIFE/FIFE/4/FIFE/3/FIFE/FIFE//INDIAN G/8/DIEHL/MI/3/PP-AUS//FIFE/ETAWAH/9/ORO/10/DIEHL/MI/3/PP-AUS//FIFE/ETAWAH/11/ORO/8/KRYMKI/7/FIFE/2*RIBA/6/FIFE/5/FIFE//FIFE/	MEXICO
TX113	MILAN/KAUZ/21/FRTR/4/RT//SPIJK/SHD/3/AKA/5/HRC/FIFE//IM/3/HRC/FIFE//KRYMKI/4/BTA/AMO44D//BTA/CHI/15/H/4/3*HRC/FIFE//IM/3/HRC/FIFE//KRYMKI/5/FA/6/KT/7/FRTR/4/RT//SPIJK/SHD/3/AKA/13/DRM/MI//KRYMKI/12/KRYMKI/7/FIFE/2*RIBA/6/FIFE/5/FIFE//FIFE/FIFE/4/FIF	MEXICO

	E/3/FIFE/FIFE//INDIAN G/8/DIEHL/MI/3/PP-AUS//FIFE/ETAWAH/9/ORO/10/DIEHL/MI/3/PP-AUS//FIFE/ETAWAH/11/ORO/8/KRYMKI/7/FIFE/2*RIBA/6/FIFE/5/FIFE//FIFE/FIFE/4/FIFE/3/FIFE/FIFE//INDIAN G/14/2*H/4/3*HRC/FIFE//IM/3/HRC/FIFE//KRYMKI/5/FA/6/EGYPT NA101/7/SWD/T.TIMOPHEEVII/16/II55.10/9/HRC/FIFE//IM/3/HRC/FIFE//KRYMKI/6/RL-2265/5/3*HRC/FIFE/3/HRC/FIFE//GEHUN/FRASER/4/HRC/FIFE//KRYMKI/7/II52.329/8/II53.388/III58.4/7/H//BOBIN*2/GAZA/6/RED EGYPTIAN(PI-170925)/KENYA BF4-3B.10.V.1/5/H/4/3*HRC/FIFE//IM/3/HRC/FIFE//KRYMKI/17/FRTR/4/RT//SPIJK/SHD/3/AKA/5/HRC/FIFE//IM/3/HRC/FIFE//KRYMKI/4/BTA/AMO44D//BTA/CHI/15/H/4/3*HRC/FIFE//IM/3/HRC/FIFE//KRYMKI/5/FA/6/KT/7/FRTR/4/RT//SPIJK/SHD/3/AKA/13/DRM/MI//KRYMKI/12/KRYMKI/7/FIFE/2*RIBA/6/FIFE/5/FIFE//FIFE/FIFE/4/FIFE/3/FIFE/FIFE//INDIAN G/8/DIEHL/MI/3/PP-AUS//FIFE/ETAWAH/9/ORO/10/DIEHL/MI/3/PP-AUS//FIFE/ETAWAH/11/ORO/8/KRYMKI/7/FIFE/2*RIBA/6/FIFE/5/FIFE//FIFE/FIFE/4/FIFE/3/FIFE/FIFE//INDIAN G/14/2*H/4/3*HRC/FIFE//IM/3/HRC/FIFE//KRYMKI/5/FA/6/EGYPT NA101/7/SWD/T.TIMOPHEEVII/16/CNO/18/PLO/19/VEE/20/KAUZ	
TX114	VEE/19/KT/6/H/4/3*HRC/FIFE//IM/3/HRC/FIFE//KRYMKI/5/FA/7/H/4/3*HRC/FIFE//IM/3/HRC/FIFE//KRYMKI/5/FA/6/KT/8/KT/5/FRTR/4/RT//SPIJK/SHD/3/AKA/6/H/4/3*HRC/FIFE//IM/3/HRC/FIFE//KRYMKI/5/FA/9/SWD/T.TIMOPHEEVII//RED EGYPTIAN(PI-170925)/KENYA BF4-3B.10.V.1/4/GB/KENIA RF 324/3/BTA/CHI//AMO25E/PELON 33C/16/LR64/15/FRTR/4/RT//SPIJK/SHD/3/AKA/5/RED EGYPTIAN(PI-170925)/KENYA BF4-3B.10.V.1/6/H/4/3*HRC/FIFE//IM/3/HRC/FIFE//KRYMKI/13/DRM/MI//KRYMKI/12/KRYMKI/7/FIFE/2*RIBA/6/FIFE/5/FIFE//FIFE/FIFE/4/FIFE/3/FIFE/FIFE//INDIAN G/8/DIEHL/MI/3/PP-AUS//FIFE/ETAWAH/9/ORO/10/DIEHL/MI/3/PP-AUS//FIFE/ETAWAH/11/ORO/8/KRYMKI/7/FIFE/2*RIBA/6/FIFE/5/FIFE//FIFE/FIFE/4/FIFE/3/FIFE/FIFE//INDIAN G/14/SWD/T.TIMOPHEEVII//RED EGYPTIAN(PI-170925)/KENYA BF4-3B.10.V.1/4/GB/KENIA RF 324/3/BTA/CHI//AMO25E/PELON 33C/17/LR64/15/H/4/3*HRC/FIFE//IM/3/HRC/FIFE//KRYMKI/5/FA/6/KT/7/FRTR/4/RT//SPIJK/SHD/3/AKA/13/DRM/MI//KRYMKI/12/KRYMKI/7/FIFE/2*RIBA/6/FIFE/5/FIFE//FIFE/FIFE/4/FIFE/3/FIFE/FIFE//INDIAN G/8/DIEHL/MI/3/PP-AUS//FIFE/ETAWAH/9/ORO/10/DIEHL/MI/3/PP-AUS//FIFE/ETAWAH/11/ORO/8/KRYMKI/7/FIFE/2*RIBA/6/FIFE/5/FIFE//FIFE/FIFE/4/FIFE/3/FIFE/FIFE//INDIAN G/14/2*H/4/3*HRC/FIFE//IM/3/HRC/FIFE//KRYMKI/5/FA/6/EGYPT NA101/7/SWD/T.TIMOPHEEVII/18/BJY/20/F3.71/26591-1T-7M-OY-115Y-OM/21/2*WEAVER/22/HAHN/2*WEAVER/23/WEAVER	MEXICO
TX115	69-1776/663//7*KAUZ	MEXICO
TX116	VEE/BOW/19/BJY/18/KT/6/H/4/3*HRC/FIFE//IM/3/HRC/FIFE//KRYMKI/5/FA/7/H/4/3*HRC/FIFE//IM/3/HRC/FIFE//KRYMKI/5/FA/6/KT/8/KT/5/FRTR/4/RT//SPIJK/SHD/3/AKA/6/H/4/3*HRC/FIFE//IM/3/HRC/FIFE//KRYMKI/5/FA/9/SWD/T.TIMOPHEEVII//RED EGYPTIAN(PI-170925)/KENYA BF4-3B.10.V.1/4/GB/KENIA RF 324/3/BTA/CHI//AMO25E/PELON 33C/16/LR64/15/FRTR/4/RT//SPIJK/SHD/3/AKA/5/RED EGYPTIAN(PI-170925)/KENYA BF4-3B.10.V.1/6/H/4/3*HRC/FIFE//IM/3/HRC/FIFE//KRYMKI/13/DRM/MI//KRYMKI/12/KRYMKI/7/FIFE/2*RIBA/6/FIFE/5/FIFE//FIFE/FIFE/4/FIFE/3/FIFE/FIFE//INDIAN G/8/DIEHL/MI/3/PP-AUS//FIFE/ETAWAH/9/ORO/10/DIEHL/MI/3/PP-AUS//FIFE/ETAWAH/11/ORO/8/KRYMKI/7/FIFE/2*RIBA/6/FIFE/5/FIFE//FIFE/FIFE/4/FIFE/3/FIFE/FIFE//INDIAN G/14/SWD/T.TIMOPHEEVII//RED EGYPTIAN(PI-170925)/KENYA BF4-3B.10.V.1/4/GB/KENIA RF 324/3/BTA/CHI//AMO25E/PELON 33C/17/LR64/15/H/4/3*HRC/FIFE//IM/3/HRC/FIFE//KRYMKI/5/FA/6/KT/7/FRTR/4/RT//SPIJK/SHD/3/AKA/13/DRM/MI//KRYMKI/12/KRYMKI/7/FIFE/2*RIBA/6/FIFE/5/FIFE//FIFE/FIFE/4/FIFE/3/FIFE/FIFE//INDIAN G/8/DIEHL/MI/3/PP-AUS//FIFE/ETAWAH/9/ORO/10/DIEHL/MI/3/PP-AUS//FIFE/ETAWAH/11/ORO/8/KRYMKI/7/FIFE/2*RIBA/6/FIFE/5/FIFE//FIFE/FIFE/4/FIFE/3/FIFE/FIFE//INDIAN G/14/2*H/4/3*HRC/FIFE//IM/3/HRC/FIFE//KRYMKI/5/FA/6/EGYPT NA101/7/SWD/T.TIMOPHEEVII/20/PASTOR	MEXICO
TX117	CAR422/18/KT/6/H/4/3*HRC/FIFE//IM/3/HRC/FIFE//KRYMKI/5/FA/7/H/4/3*HRC/FIFE//IM/3/HRC/FIFE//KRYMKI/5/FA/6/KT/8/KT/5/FRTR/4/RT//SPIJK/SHD/3/AKA/6/H/4/3*HRC/FIFE//IM/3/HRC/FIFE//KRYMKI/5/FA/9/SWD/T.TIMOPHEEVII//RED EGYPTIAN(PI-170925)/KENYA BF4-3B.10.V.1/4/GB/KENIA RF 324/3/BTA/CHI//AMO25E/PELON 33C/16/LR64/15/FRTR/4/RT//SPIJK/SHD/3/AKA/5/RED EGYPTIAN(PI-170925)/KENYA BF4-3B.10.V.1/6/H/4/3*HRC/FIFE//IM/3/HRC/FIFE//KRYMKI/13/DRM/MI//KRYMKI/12/KRYMKI/7/FIFE/2*RIBA/6/FIFE/5/FIFE//FIFE/FIFE/4/FIFE/3/FIFE/FIFE//INDIAN G/8/DIEHL/MI/3/PP-AUS//FIFE	MEXICO
TX118	BOW/BUC	MEXICO
TX119	MGP/SAP//VEE	MEXICO
TX120	HUAC/17/H/4/3*HRC/FIFE//IM/3/HRC/FIFE//KRYMKI/5/FA/6/KT/7/FRTR/4/RT//SPIJK/SHD/3/AKA/13/DRM/MI//KRYMKI/12/KRYMKI/7/FIFE/2*RIBA/6/FIFE/5/FIFE//FIFE/FIFE/4/FIFE/3/FIFE/FIFE//INDIAN G/8/DIEHL/MI/3/PP-AUS//FIFE/ETAWAH/9/ORO/10/DIEHL/MI/3/PP-AUS//FIFE/ETAWAH/11/ORO/8/KRYMKI/7/FIFE/2*RIBA/6/FIFE/5/FIFE//FIFE/FIFE/4/FIFE/3/FIFE/FIFE//INDIAN G/14/2*H/4/3*HRC/FIFE//IM/3/HRC/FIFE//KRYMKI/5/FA/6/EGYPT NA101/7/SWD/T.TIMOPHEEVII/15/CNO/16/LR64/15/H/4/3*HRC/FIFE//IM/3/HRC/FIFE//KRYMKI/5/FA/6/KT/7/FRTR/4/RT//SPIJK/SHD/3/AKA/13/DRM/MI//KRYMKI/12/KRYMKI/7/FIFE/2*RIBA/6/FIFE/5/FIFE//FIFE/FIFE/4/FIFE/3/FIFE/FIFE//INDIAN G/8/DIEHL/MI/3/PP-AUS//FIFE/ETAWAH/9/ORO/10/DIEHL/MI/3/PP-AUS//FIFE/ETAWAH/11/ORO/8/KRYMKI/7/FIFE/2*RIBA/6/FIFE/5/FIFE//FIFE/FIFE/4/FIFE/3/FIFE/FIFE//INDIAN G/14/2*H/4/3*HRC/FIFE//IM/3/HRC/FIFE//KRYMKI/5/FA/6/EGYPT	MEXICO

	NA101/7/SWD/T.TIMOPHEEVII/18/ATR*2/15/FRTR/4/RT//SPIJK/SHD/3/AKA/5/RED<br>EGYPTIAN(PI-170925)/KENYA BF4-<br>3B.10.V.1/6/H/4/3*HRC/FIFE//IM/3/HRC/FIFE//KRYMKI/13/DRM/MI//KRYMKI/12/KRYMKI/7/FIF<br>E/2*RIBA/6/FIFE/5/FIFE//FIFE/FIFE/4/FIFE/3/FIFE/FIFE//INDIAN G/8/DIEHL/MI/3/PP-<br>AUS//FIFE/ETAWAH/9/ORO/10/DIEHL/MI/3/PP-<br>AUS//FIFE/ETAWAH/11/ORO/8/KRYMKI/7/FIFE/2*RIBA/6/FIFE/5/FIFE//FIFE/FIFE/4/FIFE/3/FIFE/<br>FIFE//INDIAN G/14/SWD/T.TIMOPHEEVII//RED EGYPTIAN(PI-170925)/KENYA BF4-<br>3B.10.V.1/4/GB/KENIA RF 324/3/BTA/CHI//AMO25E/PELON<br>33C/16/BJY/21/T.AEST/19/TP/17/CNO/16/LR64/15/H/4/3*HRC/FIFE//IM/3/HRC/FIFE//KRYMKI/5/F<br>A/6/KT/7/FRTR/4/RT//SPIJK/SHD/3/AKA/13/DRM/MI//KRYMKI/12/KRYMKI/7/FIFE/2*RIBA/6/FIF<br>E/5/FIFE//FIFE/FIFE/4/FIFE/3/FIFE/FIFE//INDIAN G/8/DIEHL/MI/3/PP-<br>AUS//FIFE/ETAWAH/9/ORO/10/DIEHL/MI/3/PP-<br>AUS//FIFE/ETAWAH/11/ORO/8/KRYMKI/7/FIFE/2*RIBA/6/FIFE/5/FIFE//FIFE/FIFE/4/FIFE/3/FIFE/<br>FIFE//INDIAN G/14/2*H/4/3*HRC/FIFE//IM/3/HRC/FIFE//KRYMKI/5/FA/6/EGYPT<br>NA101/7/SWD/T.TIMOPHEEVII/18/CNO/15/FRTR/4/RT//SPIJK/SHD/3/AKA/5/RED EGYPTIAN(PI-<br>170925)/KENYA BF4-<br>3B.10.V.1/6/H/4/3*HRC/FIFE//IM/3/HRC/FIFE//KRYMKI/13/DRM/MI//KRYMKI/12/KRYMKI/7/FIF<br>E/2*RIBA/6/FIFE/5/FIFE//FIFE/FIFE/4/FIFE/3/FIFE/FIFE//INDIAN G/8/DIEHL/MI/3/PP-<br>AUS//FIFE/ETAWAH/9/ORO/10/DIEHL/MI/3/PP-<br>AUS//FIFE/ETAWAH/11/ORO/8/KRYMKI/7/FIFE/2*RIBA/6/FIFE/5/FIFE//FIFE/FIFE/4/FIFE/3/FIFE/<br>FIFE//INDIAN G/14/SWD/T.TIMOPHEEVII//RED EGYPTIAN(PI-170925)/KENYA BF4-<br>3B.10.V.1/4/GB/KENIA RF 324/3/BTA/CHI//AMO25E/PELON<br>33C/20/KT/6/H/4/3*HRC/FIFE//IM/3/HRC/FIFE//KRYMKI/5/FA/7/H/4/3*HRC/FIFE//IM/3/HRC/FIFE<br>//KRYMKI/5/FA/6/KT/8/KT/5/FRTR/4/RT//SPIJK/SHD/3/AKA/6/H/4/3*HRC/FIFE//IM/3/HRC/FIFE//<br>KRYMKI/5/FA/9/SWD/T.TIMOPHEEVII//RED EGYPTIAN(PI-170925)/KENYA BF4-<br>3B.10.V.1/4/GB/KENIA RF 324/3/BTA/CHI//AMO25E/PELON<br>33C/16/LR64/15/FRTR/4/RT//SPIJK/SHD/3/AKA/5/RED EGYPTIAN(PI-170925)/KENYA BF4-<br>3B.10.V.1/6/H/4/3*HRC/FIFE//IM/3/HRC/FIFE//KRYMKI/13/DRM/MI//KRYMKI/12/KRYMKI/7/FIF<br>E/2*RIBA/6/FIFE/5/FIFE//FIFE/FIFE/4/FIFE/3/FIFE/FIFE//INDIAN G/8/DIEHL/MI/3/PP-<br>AUS//FIFE/ETAWAH/9/ORO/10/DIEHL/MI/3/PP-<br>AUS//FIFE/ETAWAH/11/ORO/8/KRYMKI/7/FIFE/2*RIBA/6/FIFE/5/FIFE//FIFE/FIFE/4/FIFE/3/FIFE/<br>FIFE//INDIAN G/14/SWD/T.TIMOPHEEVII//RED EGYPTIAN(PI-170925)/KENYA BF4-<br>3B.10.V.1/4/GB/KENIA RF 324/3/BTA/CHI//AMO25E/PELON<br>33C/17/LR64/15/H/4/3*HRC/FIFE//IM/3/HRC/FIFE//KRYMKI/5/FA/6/KT/7/FRTR/4/RT//SPIJK/SHD/<br>3/AKA/13/DRM/MI//KRYMKI/12/KRYMKI/7/FIFE/2*RIBA/6/FIFE/5/FIFE//FIFE/FIFE/4/FIFE/3/FIF<br>E/FIFE//INDIAN G/8/DIEHL/MI/3/PP-AUS//FIFE/ETAWAH/9/ORO/10/DIEHL/MI/3/PP-<br>AUS//FIFE/ETAWAH/11/ORO/8/KRYMKI/7/FIFE/2*RIBA/6/FIFE/5/FIFE//FIFE/FIFE/4/FIFE/3/FIFE/<br>FIFE//INDIAN G/14/2*H/4/3*HRC/FIFE//IM/3/HRC/FIFE//KRYMKI/5/FA/6/EGYPT<br>NA101/7/SWD/T.TIMOPHEEVII	
TX121	TNMU/22/KT/6/H/4/3*HRC/FIFE//IM/3/HRC/FIFE//KRYMKI/5/FA/7/H/4/3*HRC/FIFE//IM/3/HRC/FI<br>FE//KRYMKI/5/FA/6/KT/8/KT/5/FRTR/4/RT//SPIJK/SHD/3/AKA/6/H/4/3*HRC/FIFE//IM/3/HRC/FIF<br>E//KRYMKI/5/FA/9/SWD/T.TIMOPHEEVII//RED EGYPTIAN(PI-170925)/KENYA BF4-<br>3B.10.V.1/4/GB/KENIA RF 324/3/BTA/CHI//AMO25E/PELON<br>33C/16/LR64/15/FRTR/4/RT//SPIJK/SHD/3/AKA/5/RED EGYPTIAN(PI-170925)/KENYA BF4-<br>3B.10.V.1/6/H/4/3*HRC/FIFE//IM/3/HRC/FIFE//KRYMKI/13/DRM/MI//KRYMKI/12/KRYMKI/7/FIF<br>E/2*RIBA/6/FIFE/5/FIFE//FIFE/FIFE/4/FIFE/3/FIFE/FIFE//INDIAN G/8/DIEHL/MI/3/PP-<br>AUS//FIFE/ETAWAH/9/ORO/10/DIEHL/MI/3/PP-<br>AUS//FIFE/ETAWAH/11/ORO/8/KRYMKI/7/FIFE/2*RIBA/6/FIFE/5/FIFE//FIFE/FIFE/4/FIFE/3/FIFE/<br>FIFE//INDIAN G/14/SWD/T.TIMOPHEEVII//RED EGYPTIAN(PI-170925)/KENYA BF4-<br>3B.10.V.1/4/GB/KENIA RF 324/3/BTA/CHI//AMO25E/PELON<br>33C/17/LR64/15/H/4/3*HRC/FIFE//IM/3/HRC/FIFE//KRYMKI/5/FA/6/KT/7/FRTR/4/RT//SPIJK/SHD/<br>3/AKA/13/DRM/MI//KRYMKI/12/KRYMKI/7/FIFE/2*RIBA/6/FIFE/5/FIFE//FIFE/FIFE/4/FIFE/3/FIF<br>E/FIFE//INDIAN G/8/DIEHL/MI/3/PP-AUS//FIFE/ETAWAH/9/ORO/10/DIEHL/MI/3/PP-<br>AUS//FIFE/ETAWAH/11/ORO/8/KRYMKI/7/FIFE/2*RIBA/6/FIFE/5/FIFE//FIFE/FIFE/4/FIFE/3/FIFE/<br>FIFE//INDIAN G/14/2*H/4/3*HRC/FIFE//IM/3/HRC/FIFE//KRYMKI/5/FA/6/EGYPT<br>NA101/7/SWD/T.TIMOPHEEVII/18/BJY/21/T.AEST/19/TP/17/CNO/16/LR64/15/H/4/3*HRC/FIFE//I<br>M/3/HRC/FIFE//KRYMKI/5/FA/6/KT/7/FRTR/4/RT//SPIJK/SHD/3/AKA/13/DRM/MI//KRYMKI/12/K<br>RYMKI/7/FIFE/2*RIBA/6/FIFE/5/FIFE//FIFE/FIFE/4/FIFE/3/FIFE/FIFE//INDIAN<br>G/8/DIEHL/MI/3/PP-AUS//FIFE/ETAWAH/9/ORO/10/DIEHL/MI/3/PP-<br>AUS//FIFE/ETAWAH/11/ORO/8/KRYMKI/7/FIFE/2*RIBA/6/FIFE/5/FIFE//FIFE/FIFE/4/FIFE/3/FIFE/<br>FIFE//INDIAN G/14/2*H/4/3*HRC/FIFE//IM/3/HRC/FIFE//KRYMKI/5/FA/6/EGYPT<br>NA101/7/SWD/T.TIMOPHEEVII/18/CNO/15/FRTR/4/RT//SPIJK/SHD/3/AKA/5/RED EGYPTIAN(PI-<br>170925)/KENYA BF4-<br>3B.10.V.1/6/H/4/3*HRC/FIFE//IM/3/HRC/FIFE//KRYMKI/13/DRM/MI//KRYMKI/12/KRYMKI/7/FIF<br>E/2*RIBA/6/FIFE/5/FIFE//FIFE/FIFE/4/FIFE/3/FIFE/FIFE//INDIAN G/8/DIEHL/MI/3/PP-<br>AUS//FIFE/ETAWAH/9/ORO/10/DIEHL/MI/3/PP-<br>AUS//FIFE/ETAWAH/11/ORO/8/KRYMKI/7/FIFE/2*RIBA/6/FIFE/5/FIFE//FIFE/FIFE/4/FIFE/3/FIFE/<br>FIFE//INDIAN G/14/SWD/T.TIMOPHEEVII//RED EGYPTIAN(PI-170925)/KENYA BF4-<br>3B.10.V.1/4/GB/KENIA RF 324/3/BTA/CHI//AMO25E/PELON<br>33C/20/KT/6/H/4/3*HRC/FIFE//IM/3/HRC/FIFE//KRYMKI/5/FA/7/H/4/3*HRC/FIFE//IM/3/HRC/FIFE	MEXICO

	//KRYMKI/5/FA/6/KT/8/KT/5/FRTR/4/RT//SPIJK/SHD/3/AKA/6/H/4/3*HRC/FIFE//IM/3/HRC/FIFE//KRYMKI/5/FA/9/SWD/T.TIMOPHEEVII//RED EGYPTIAN(PI-170925)/KENYA BF4-3B.10.V.1/4/GB/KENIA RF 324/3/BTA/CHI//AMO25E/PELON 33C/16/LR64/15/FRTR/4/RT//SPIJK/SHD/3/AKA/5/RED EGYPTIAN(PI-170925)/KENYA BF4-3B.10.V.1/6/H/4/3*HRC/FIFE//IM/3/HRC/FIFE//KRYMKI/13/DRM/MI//KRYMKI/12/KRYMKI/7/FIFE/2*RIBA/6/FIFE/5/FIFE//FIFE/FIFE/4/FIFE/3/FIFE/FIFE//INDIAN G/8/DIEHL/MI/3/PP-AUS//FIFE/ETAWAH/9/ORO/10/DIEHL/MI/3/PP-AUS//FIFE/ETAWAH/11/ORO/8/KRYMKI/7/FIFE/2*RIBA/6/FIFE/5/FIFE//FIFE/FIFE/4/FIFE/3/FIFE/FIFE//INDIAN G/14/SWD/T.TIMOPHEEVII//RED EGYPTIAN(PI-170925)/KENYA BF4-3B.10.V.1/4/GB/KENIA RF 324/3/BTA/CHI//AMO25E/PELON 33C/17/LR64/15/H/4/3*HRC/FIFE//IM/3/HRC/FIFE//KRYMKI/5/FA/6/KT/7/FRTR/4/RT//SPIJK/SHD/3/AKA/13/DRM/MI//KRYMKI/12/KRYMKI/7/FIFE/2*RIBA/6/FIFE/5/FIFE//FIFE/FIFE/4/FIFE/3/FIFE/FIFE//INDIAN G/8/DIEHL/MI/3/PP-AUS//FIFE/ETAWAH/9/ORO/10/DIEHL/MI/3/PP-AUS//FIFE/ETAWAH/11/ORO/8/KRYMKI/7/FIFE/2*RIBA/6/FIFE/5/FIFE//FIFE/FIFE/4/FIFE/3/FIFE/FIFE//INDIAN G/14/2*H/4/3*HRC/FIFE//IM/3/HRC/FIFE//KRYMKI/5/FA/6/EGYPT NA101/7/SWD/T.TIMOPHEEVII	
TX122	DU TOIT//RYMER/MAFFRA/3/EGYPTIAN 4/4/KENYA C6042/5/BOBIN/6/INSIGNIA 49/7/ARONA/8/3*SCHOMBURGK	
TX123	RAC177/UNICULM492/15/H/4/3*HRC/FIFE//IM/3/HRC/FIFE//KRYMKI/5/FA/6/KT/7/FRTR/4/RT//SPIJK/SHD/3/AKA/13/DRM/MI//KRYMKI/12/KRYMKI/7/FIFE/2*RIBA/6/FIFE/5/FIFE//FIFE/FIFE/4/FIFE/3/FIFE/FIFE//INDIAN G/8/DIEHL/MI/3/PP-AUS//FIFE/ETAWAH/9/ORO/10/DIEHL/MI/3/PP-AUS//FIFE/ETAWAH/11/ORO/8/KRYMKI/7/FIFE/2*RIBA/6/FIFE/5/FIFE//FIFE/FIFE/4/FIFE/3/FIFE/FIFE//INDIAN G/14/GB/3/RAPR//CLEVARD/SANDS/4/KENYA C6042/5/DIRK 48	
TX124	FRTR/4/RT//SPIJK/SHD/3/AKA/5/RED EGYPTIAN(PI-170925)/KENYA BF4-3B.10.V.1/6/H/4/3*HRC/FIFE//IM/3/HRC/FIFE//KRYMKI/13/DRM/MI//KRYMKI/12/KRYMKI/7/FIFE/2*RIBA/6/FIFE/5/FIFE//FIFE/FIFE/4/FIFE/3/FIFE/FIFE//INDIAN G/8/DIEHL/MI/3/PP-AUS//FIFE/ETAWAH/9/ORO/10/DIEHL/MI/3/PP-AUS//FIFE/ETAWAH/11/ORO/8/KRYMKI/7/FIFE/2*RIBA/6/FIFE/5/FIFE//FIFE/FIFE/4/FIFE/3/FIFE/FIFE//INDIAN G/14/SWD/T.TIMOPHEEVII//RED EGYPTIAN(PI-170925)/KENYA BF4-3B.10.V.1/4/GB/KENIA RF 324/3/BTA/CHI//AMO25E/PELON 33C/15/ARCHON//GHURKA/C-M-G-*2/16/MEW	
TX125	DU TOIT//RYMER/MAFFRA/3/EGYPTIAN 4/4/KENYA C6042/5/BOBIN/6/INSIGNIA 49/7/ARA-A/8/3*SCHOMBURGK/9/2*MOLINEUX	
TX126	H/4/3*HRC/FIFE//IM/3/HRC/FIFE//KRYMKI/5/FA/6/KT/7/FRTR/4/RT//SPIJK/SHD/3/AKA/13/DRM/MI//KRYMKI/12/KRYMKI/7/FIFE/2*RIBA/6/FIFE/5/FIFE//FIFE/FIFE/4/FIFE/3/FIFE/FIFE//INDIAN G/8/DIEHL/MI/3/PP-AUS//FIFE/ETAWAH/9/ORO/10/DIEHL/MI/3/PP-AUS//FIFE/ETAWAH/11/ORO/8/KRYMKI/7/FIFE/2*RIBA/6/FIFE/5/FIFE//FIFE/FIFE/4/FIFE/3/FIFE/FIFE//INDIAN G/14/GHURKA/C-M-G-/15/KLK/14/H/4/3*HRC/FIFE//IM/3/HRC/FIFE//KRYMKI/5/FA/6/KT/7/FRTR/4/RT//SPIJK/SHD/3/AKA/13/DRM/MI//KRYMKI/12/KRYMKI/7/FIFE/2*RIBA/6/FIFE/5/FIFE//FIFE/FIFE/4/FIFE/3/FIFE/FIFE//INDIAN G/8/DIEHL/MI/3/PP-AUS//FIFE/ETAWAH/9/ORO/10/DIEHL/MI/3/PP-AUS//FIFE/ETAWAH/11/ORO/8/KRYMKI/7/FIFE/2*RIBA/6/FIFE/5/FIFE//FIFE/FIFE/4/FIFE/3/FIFE/FIFE//INDIAN G/16/H/4/3*HRC/FIFE//IM/3/HRC/FIFE//KRYMKI/5/FA/6/KT/7/FRTR/4/RT//SPIJK/SHD/3/AKA/13/DRM/MI//KRYMKI/12/KRYMKI/7/FIFE/2*RIBA/6/FIFE/5/FIFE//FIFE/FIFE/4/FIFE/3/FIFE/FIFE//INDIAN G/8/DIEHL/MI/3/PP-AUS//FIFE/ETAWAH	AUSTRALIA
TX127	FIFE//VNSS/INDIAN G*2/3/CRETAN/4/KAMBOURICO/5/TG/6/TOSCA/9/FLK/8/DU TOIT/3/DU TOIT//RYMER/MAFFRA/4/KENYA C6042//WAGGA 13/MS-A/3/SWORD/KENYA C6041/5/MEXDW/6/GM*2/7/BOW	
TX128	BCH/3/PWTH/CNDR//2*CNDR	
TX129	WW425	
TX130	MNV/VEE	MEXICO
TX131	BUC/PRL//CHIL	MEXICO
TX132	MGP/SAP//VEE	MEXICO
TX133	JUN/20/CNO/GLL/18/FRTR/4/RT//SPIJK/SHD/3/AKA/5/HRC/FIFE//IM/3/HRC/FIFE//KRYMKI/4/BTA/AMO44D//BTA/CHI/15/H/4/3*HRC/FIFE//IM/3/HRC/FIFE//KRYMKI/5/FA/6/KT/7/FRTR/4/RT//SPIJK/SHD/3/AKA/13/DRM/MI//KRYMKI/12/KRYMKI/7/FIFE/2*RIBA/6/FIFE/5/FIFE//FIFE/FIFE/4/FIFE/3/FIFE/FIFE//INDIAN G/8/DIEHL/MI/3/PP-AUS//FIFE/ETAWAH/9/ORO/10/DIEHL/MI/3/PP-AUS//FIFE/ETAWAH/11/ORO/8/KRYMKI/7/FIFE/2*RIBA/6/FIFE/5/FIFE//FIFE/FIFE/4/FIFE/3/FIFE/FIFE//INDIAN G/14/2*H/4/3*HRC/FIFE//IM/3/HRC/FIFE//KRYMKI/5/FA/6/EGYPT NA101/7/SWD/T.TIMOPHEEVII/16/PRESIDENTE PERON MAG/4/BTA/CHI//AMO25E/PELON 33C/3/BTA/17/BB/19/FRTR/4/RT//SPIJK/SHD/3/AKA/5/HRC/FIFE//IM/3/HRC/FIFE//KRYMKI/4/BTA/AMO44D//BTA/CHI/15/H/4/3*HRC/FIFE//IM/3/HRC/FIFE//KRYMKI/5/FA/6/KT/7/FRTR/4/RT//SPIJK/SHD/3/AKA/13/DRM/MI//KRYMKI/12/KRYMKI/7/FIFE/2*RIBA/6/FIFE/5/FIFE//FIFE/FIFE/4/FIFE/3/FIFE/FIFE//INDIAN G/8/DIEHL/MI/3/PP-AUS//FIFE/ETAWAH/9/ORO/10/DIEHL/MI/3/PP-AUS//FIFE/ETAWAH/11/ORO/8/KRYMKI/7/FIFE/2*RIBA/6/FIFE/5/FIFE//FIFE/FIFE/4/FIFE/3/FIFE/FIFE//INDIAN G/14/2*H/4/3*HRC/FIFE//IM/3/HRC/FIFE//KRYMKI/5/FA/6/EGYPT NA101/7/SWD/T.TIMOPHEEVII/16/FRTR/4/RT//SPIJK/SHD/3/AKA/5/HRC/FIFE//IM/3/HRC/FIFE//	MEXICO

	KRYMKI/4/BTA/AMO44D//BTA/CHI*2/7/KT/5/FRTR/4/RT//SPIJK/SHD/3/AKA/6/H/4/3*HRC/FIFE/<br>/IM/3/HRC/FIFE//KRYMKI/5/FA	
TX134	TAM200/TUI	MEXICO
TX135	VEE*5/6/ARGUS/4/6*HRC/FIFE//IM/3/HRC/FIFE//KRYMKI/5/6*BB	MEXICO
TX136	LHNKE/AE.SQUARROSA (205)//KAUZ	
TX137	HXL7573/2*BAU	MEXICO
TX138	DHARWAR DRY	
TX139	LR64/15/H/4/3*HRC/FIFE//IM/3/HRC/FIFE//KRYMKI/5/FA/6/KT/7/FRTR/4/RT//SPIJK/SHD/3/AKA/<br>13/DRM/MI//KRYMKI/12/KRYMKI/7/FIFE/2*RIBA/6/FIFE/5/FIFE//FIFE/FIFE/4/FIFE/3/FIFE/FIFE//<br>INDIAN G/8/DIEHL/MI/3/PP-AUS//FIFE/ETAWAH/9/ORO/10/DIEHL/MI/3/PP-<br>AUS//FIFE/ETAWAH/11/ORO/8/KRYMKI/7/FIFE/2*RIBA/6/FIFE/5/FIFE//FIFE/FIFE/4/FIFE/3/FIFE/<br>FIFE//INDIAN G/14/2*H/4/3*HRC/FIFE//IM/3/HRC/FIFE//KRYMKI/5/FA/6/EGYPT<br>NA101/7/SWD/T.TIMOPHEEVII/16/FRTR/4/RT//SPIJK/SHD/3/AKA/5/C.O./C.R./7/FRTR/4/RT//SPIJ<br>K/SHD/3/AKA/6/H/4/3*HRC/FIFE//IM/3/HRC/FIFE//KRYMKI/5/FA/8/FRTR/4/RT//SPIJK/SHD/3/AK<br>A/5/C.O./C.R./6/LD/FIFE//RIGA<br>M/7/KT/6/H/4/3*HRC/FIFE//IM/3/HRC/FIFE//KRYMKI/5/FA/17/CNO/15/FRTR/4/RT//SPIJK/SHD/3/<br>AKA/5/RED EGYPTIAN(PI-170925)/KENYA BF4-<br>3B.10.V.1/6/H/4/3*HRC/FIFE//IM/3/HRC/FIFE//KRYMKI/13/DRM/MI//KRYMKI/12/KRYMKI/7/FIF<br>E/2*RIBA/6/FIFE/5/FIFE//FIFE/FIFE/4/FIFE/3/FIFE/FIFE//INDIAN G/8/DIEHL/MI/3/PP-<br>AUS//FIFE/ETAWAH/9/ORO/10/DIEHL/MI/3/PP-<br>AUS//FIFE/ETAWAH/11/ORO/8/KRYMKI/7/FIFE/2*RIBA/6/FIFE/5/FIFE//FIFE/FIFE/4/FIFE/3/FIFE/<br>FIFE//INDIAN G/14/SWD/T.TIMOPHEEVII//RED EGYPTIAN(PI-170925)/KENYA BF4-<br>3B.10.V.1/4/GB/KENIA RF 324/3/BTA/CHI//AMO25E/PELON<br>33C/18/FRTR/4/RT//SPIJK/SHD/3/AKA/5/RED EGYPTIAN(PI-170925)/KENYA BF4-<br>3B.10.V.1/6/H/4/3*HRC/FIFE//IM/3/HRC/FIFE//KRYMKI/13/DRM/MI//KRYMKI/12/KRYMKI/7/FIF<br>E/2*RIBA/6/FIFE/5/FIFE//FIFE/FIFE/4/FIFE/3/FIFE/FIFE//INDIAN G/8/DIEHL/MI/3/PP-<br>AUS//FIFE/ETAWAH/9/ORO/10/DIEHL/MI/3/PP-<br>AUS//FIFE/ETAWAH/11/ORO/8/KRYMKI/7/FIFE/2*RIBA/6/FIFE/5/FIFE//FIFE/FIFE/4/FIFE/3/FIFE/<br>FIFE//INDIAN G/14/SWD/T.TIMOPHEEVII//RED EGYPTIAN(PI-170925)/KENYA BF4-<br>3B.10.V.1/4/GB/KENIA RF 324/3/BTA/CHI//AMO25E/PELON 33C/15/BB	MEXICO
TX140	KT/6/H/4/3*HRC/FIFE//IM/3/HRC/FIFE//KRYMKI/5/FA/7/H/4/3*HRC/FIFE//IM/3/HRC/FIFE//KRY<br>MKI/5/FA/6/KT/8/KT/5/FRTR/4/RT//SPIJK/SHD/3/AKA/6/H/4/3*HRC/FIFE//IM/3/HRC/FIFE//KRYM<br>KI/5/FA/9/SWD/T.TIMOPHEEVII//RED EGYPTIAN(PI-170925)/KENYA BF4-<br>3B.10.V.1/4/GB/KENIA RF 324/3/BTA/CHI//AMO25E/PELON<br>33C/16/LR64/15/FRTR/4/RT//SPIJK/SHD/3/AKA/5/RED EGYPTIAN(PI-170925)/KENYA BF4-<br>3B.10.V.1/6/H/4/3*HRC/FIFE//IM/3/HRC/FIFE//KRYMKI/13/DRM/MI//KRYMKI/12/KRYMKI/7/FIF<br>E/2*RIBA/6/FIFE/5/FIFE//FIFE/FIFE/4/FIFE/3/FIFE/FIFE//INDIAN G/8/DIEHL/MI/3/PP-<br>AUS//FIFE/ETAWAH/9/ORO/10/DIEHL/MI/3/PP-<br>AUS//FIFE/ETAWAH/11/ORO/8/KRYMKI/7/FIFE/2*RIBA/6/FIFE/5/FIFE//FIFE/FIFE/4/FIFE/3/FIFE/<br>FIFE//INDIAN G/14/SWD/T.TIMOPHEEVII//RED EGYPTIAN(PI-170925)/KENYA BF4-<br>3B.10.V.1/4/GB/KENIA RF 324/3/BTA/CHI//AMO25E/PELON<br>33C/17/LR64/15/H/4/3*HRC/FIFE//IM/3/HRC/FIFE//KRYMKI/5/FA/6/KT/7/FRTR/4/RT//SPIJK/SHD/<br>3/AKA/13/DRM/MI//KRYMKI/12/KRYMKI/7/FIFE/2*RIBA/6/FIFE/5/FIFE//FIFE/FIFE/4/FIFE/3/FIF<br>E/FIFE//INDIAN G/8/DIEHL/MI/3/PP-AUS//FIFE/ETAWAH/9/ORO/10/DIEHL/MI/3/PP-<br>AUS//FIFE/ETAWAH/11/ORO/8/KRYMKI/7/FIFE/2*RIBA/6/FIFE/5/FIFE//FIFE/FIFE/4/FIFE/3/FIFE/<br>FIFE//INDIAN G/14/2*H/4/3*HRC/FIFE//IM/3/HRC/FIFE//KRYMKI/5/FA/6/EGYPT<br>NA101/7/SWD/T.TIMOPHEEVII/18/BJY/19/VEE	MEXICO
TX141	W3918A/18/KT/6/H/4/3*HRC/FIFE//IM/3/HRC/FIFE//KRYMKI/5/FA/7/H/4/3*HRC/FIFE//IM/3/HRC/<br>FIFE//KRYMKI/5/FA/6/KT/8/KT/5/FRTR/4/RT//SPIJK/SHD/3/AKA/6/H/4/3*HRC/FIFE//IM/3/HRC/FI<br>FE//KRYMKI/5/FA/9/SWD/T.TIMOPHEEVII//RED EGYPTIAN(PI-170925)/KENYA BF4-<br>3B.10.V.1/4/GB/KENIA RF 324/3/BTA/CHI//AMO25E/PELON<br>33C/16/LR64/15/FRTR/4/RT//SPIJK/SHD/3/AKA/5/RED EGYPTIAN(PI-170925)/KENYA BF4-<br>3B.10.V.1/6/H/4/3*HRC/FIFE//IM/3/HRC/FIFE//KRYMKI/13/DRM/MI//KRYMKI/12/KRYMKI/7/FIF<br>E/2*RIBA/6/FIFE/5/FIFE//FIFE/FIFE/4/FIFE/3/FIFE/FIFE//INDIAN G/8/DIEHL/MI/3/PP-<br>AUS//FIFE/ETAWAH/9/ORO/10/DIEHL/MI/3/PP-<br>AUS//FIFE/ETAWAH/11/ORO/8/KRYMKI/7/FIFE/2*RIBA/6/FIFE/5/FIFE//FIFE/FIFE/4/FIFE/3/FIFE/<br>FIFE//INDIAN G/14/SWD/T.TIMOPHEEVII//RED EGYPTIAN(PI-170925)/KENYA BF4-<br>3B.10.V.1/4/GB/KENIA RF 324/3/BTA/CHI//AMO25E/PELON<br>33C/17/LR64/15/H/4/3*HRC/FIFE//IM/3/HRC/FIFE//KRYMKI/5/FA/6/KT/7/FRTR/4/RT//SPIJK/SHD/<br>3/AKA/13/DRM/MI//KRYMKI/12/KRYMKI/7/FIFE/2*RIBA/6/FIFE/5/FIFE//FIFE/FIFE/4/FIFE/3/FIF<br>E/FIFE//INDIAN G/8/DIEHL/MI/3/PP-AUS//FIFE/ETAWAH/9/ORO/10/DIEHL/MI/3/PP-<br>AUS//FIFE/ETAWAH/11/ORO/8/KRYMKI/7/FIFE/2*RIBA/6/FIFE/5/FIFE//FIFE/FIFE/4/FIFE/3/FIFE/<br>FIFE//INDIAN G/14/2*H/4/3*HRC/FIFE//IM/3/HRC/FIFE//KRYMKI/5/FA/6/EGYPT<br>NA101/7/SWD/T.TIMOPHEEVII	SYRIA
TX142	PASTOR/BABAX	MEXICO
TX143	D65152/D6148//R143/3/ENTE/STR/4/AEGILOPS SQUARROSA (TAUS)/5/WEAVER/6/PASTOR	MEXICO
TX144	IRENA/BABAX//PASTOR	MEXICO
TX145	LHNKE/AE.SQUARROSA<br>(224)/19/BJY/18/KT/6/H/4/3*HRC/FIFE//IM/3/HRC/FIFE//KRYMKI/5/FA/7/H/4/3*HRC/FIFE//IM/3/H	MEXICO

	RC/FIFE//KRYMKI/5/FA/6/KT/8/KT/5/FRTR/4/RT//SPIJK/SHD/3/AKA/6/H/4/3*HRC/FIFE//IM/3/HR<br>C/FIFE//KRYMKI/5/FA/9/SWD/T.TIMOPHEEVII//RED EGYPTIAN(PI-170925)/KENYA BF4-<br>3B.10.V.1/4/GB/KENIA RF 324/3/BTA/CHI//AMO25E/PELON<br>33C/16/LR64/15/FRTR/4/RT//SPIJK/SHD/3/AKA/5/RED EGYPTIAN(PI-170925)/KENYA BF4-<br>3B.10.V.1/6/H/4/3*HRC/FIFE//IM/3/HRC/FIFE//KRYMKI/13/DRM/MI//KRYMKI/12/KRYMKI/7/FIF<br>E/2*RIBA/6/FIFE/5/FIFE//FIFE/FIFE/4/FIFE/3/FIFE/FIFE//INDIAN G/8/DIEHL/MI/3/PP-<br>AUS//FIFE/ETAWAH/9/ORO/10/DIEHL/MI/3/PP-<br>AUS//FIFE/ETAWAH/11/ORO/8/KRYMKI/7/FIFE/2*RIBA/6/FIFE/5/FIFE//FIFE/FIFE/4/FIFE/3/FIFE/<br>FIFE//INDIAN G/14/SWD/T.TIMOPHEEVII//RED EGYPTIAN(PI-170925)/KENYA BF4-<br>3B.10.V.1/4/GB/KENIA RF 324/3/BTA/CHI//AMO25E/PELON<br>33C/17/LR64/15/H/4/3*HRC/FIFE//IM/3/HRC/FIFE//KRYMKI/5/FA/6/KT/7/FRTR/4/RT//SPIJK/SHD/<br>3/AKA/13/DRM/MI//KRYMKI/12/KRYMKI/7/FIFE/2*RIBA/6/FIFE/5/FIFE//FIFE/FIFE/4/FIFE/3/FIF<br>E/FIFE//INDIAN G/8/DIEHL/MI/3/PP-AUS//FIFE/ETAWAH/9/ORO/10/DIEHL/MI/3/PP-<br>AUS//FIFE/ETAWAH/11/ORO/8/KRYMKI/7/FIFE/2*RIBA/6/FIFE/5/FIFE//FIFE/FIFE/4/FIFE/3/FIFE/<br>FIFE//INDIAN G/14/2*H/4/3*HRC/FIFE//IM/3/HRC/FIFE//KRYMKI/5/FA/6/EGYPT<br>NA101/7/SWD/T.TIMOPHEEVII/20/PASTOR	
TX146	GA/AE.SQUARROSA (224)//2*YACO/3/BABAX	MEXICO
TX147	MILAN/KAUZ//DHARWAR DRY/3/BABAX	MEXICO
TX148	KABY/BABAX/20/LHNKE/AE.SQUARROSA<br>(224)/19/BJY/18/KT/6/H/4/3*HRC/FIFE//IM/3/HRC/FIFE//KRYMKI/5/FA/7/H/4/3*HRC/FIFE//IM/3/H<br>RC/FIFE//KRYMKI/5/FA/6/KT/8/KT/5/FRTR/4/RT//SPIJK/SHD/3/AKA/6/H/4/3*HRC/FIFE//IM/3/HR<br>C/FIFE//KRYMKI/5/FA/9/SWD/T.TIMOPHEEVII//RED EGYPTIAN(PI-170925)/KENYA BF4-<br>3B.10.V.1/4/GB/KENIA RF 324/3/BTA/CHI//AMO25E/PELON<br>33C/16/LR64/15/FRTR/4/RT//SPIJK/SHD/3/AKA/5/RED EGYPTIAN(PI-170925)/KENYA BF4-<br>3B.10.V.1/6/H/4/3*HRC/FIFE//IM/3/HRC/FIFE//KRYMKI/13/DRM/MI//KRYMKI/12/KRYMKI/7/FIF<br>E/2*RIBA/6/FIFE/5/FIFE//FIFE/FIFE/4/FIFE/3/FIFE/FIFE//INDIAN G/8/DIEHL/MI/3/PP-<br>AUS//FIFE/ETAWAH/9/ORO/10/DIEHL/MI/3/PP-<br>AUS//FIFE/ETAWAH/11/ORO/8/KRYMKI/7/FIFE/2*RIBA/6/FIFE/5/FIFE//FIFE/FIFE/4/FIFE/3/FIFE/<br>FIFE//INDIAN G/14/SWD/T.TIMOPHEEVII//RED EGYPTIAN(PI-170925)/KENYA BF4-<br>3B.10.V.1/4/GB/KENIA RF 324/3/BTA/CHI//AMO25E/PELON<br>33C/17/LR64/15/H/4/3*HRC/FIFE//IM/3/HRC/FIFE//KRYMKI/5/FA/6/KT/7/FRTR/4/RT//SPIJK/SHD/<br>3/AKA/13/DRM/MI//KRYMKI/12/KRYMKI/7/FIFE/2*RIBA/6/FIFE/5/FIFE//FIFE/FIFE/4/FIFE/3/FIF<br>E/FIFE//INDIAN G/8/DIEHL/MI/3/PP-AUS//FIFE/ETAWAH/9/ORO/10/DIEHL/MI/3/PP-<br>AUS//FIFE/ETAWAH/11/ORO/8/KRYMKI/7/FIFE/2*RIBA/6/FIFE/5/FIFE//FIFE/FIFE/4/FIFE/3/FIFE/<br>FIFE//INDIAN G/14/2*H/4/3*HRC/FIFE//IM/3/HRC/FIFE//KRYMKI/5/FA/6/EGYPT<br>NA101/7/SWD/T.TIMOPHEEVII	MEXICO
TX149	BJY/18/KT/6/H/4/3*HRC/FIFE//IM/3/HRC/FIFE//KRYMKI/5/FA/7/H/4/3*HRC/FIFE//IM/3/HRC/FIFE<br>//KRYMKI/5/FA/6/KT/8/KT/5/FRTR/4/RT//SPIJK/SHD/3/AKA/6/H/4/3*HRC/FIFE//IM/3/HRC/FIFE//<br>KRYMKI/5/FA/9/SWD/T.TIMOPHEEVII//RED EGYPTIAN(PI-170925)/KENYA BF4-<br>3B.10.V.1/4/GB/KENIA RF 324/3/BTA/CHI//AMO25E/PELON<br>33C/16/LR64/15/FRTR/4/RT//SPIJK/SHD/3/AKA/5/RED EGYPTIAN(PI-170925)/KENYA BF4-<br>3B.10.V.1/6/H/4/3*HRC/FIFE//IM/3/HRC/FIFE//KRYMKI/13/DRM/MI//KRYMKI/12/KRYMKI/7/FIF<br>E/2*RIBA/6/FIFE/5/FIFE//FIFE/FIFE/4/FIFE/3/FIFE/FIFE//INDIAN G/8/DIEHL/MI/3/PP-<br>AUS//FIFE/ETAWAH/9/ORO/10/DIEHL/MI/3/PP-<br>AUS//FIFE/ETAWAH/11/ORO/8/KRYMKI/7/FIFE/2*RIBA/6/FIFE/5/FIFE//FIFE/FIFE/4/FIFE/3/FIFE/<br>FIFE//INDIAN G/14/SWD/T.TIMOPHEEVII//RED EGYPTIAN(PI-170925)/KENYA BF4-<br>3B.10.V.1/4/GB/KENIA RF 324/3/BTA/CHI//AMO25E/PELON<br>33C/17/LR64/15/H/4/3*HRC/FIFE//IM/3/HRC/FIFE//KRYMKI/5/FA/6/KT/7/FRTR/4/RT//SPIJK/SHD/<br>3/AKA/13/DRM/MI//KRYMKI/12/KRYMKI/7/FIFE/2*RIBA/6/FIFE/5/FIFE//FIFE/FIFE/4/FIFE/3/FIF<br>E/FIFE//INDIAN G/8/DIEHL/MI/3/PP-AUS//FIFE/ETAWAH/9/ORO/10/DIEHL/MI/3/PP-<br>AUS//FIFE/ETAWAH/11/ORO/8/KRYMKI/7/FIFE/2*RIBA/6/FIFE/5/FIFE//FIFE/FIFE/4/FIFE/3/FIFE/<br>FIFE//INDIAN G/14/2*H/4/3*HRC/FIFE//IM/3/HRC/FIFE//KRYMKI/5/FA/6/EGYPT<br>NA101/7/SWD/T.TIMOPHEEVII/19/PRL/BOW/20/MILAN/KAUZ/21/BABAX	MEXICO
TX150	ATTILA/BABAX//PASTOR	MEXICO
TX151	S-24/LUTESCENS-55-11	RUSSIA
TX152	TSELINNAYA-YUBILEINAYA	KAZAKHSTAN
TX153	OMSKAYA-32	
TX154	KE FENG 2	
TX155	NEW LONG MAI 19	CHINA
TX156	LONG MAI 23	CHINA
TX157	RL-4031/5/HRC/FIFE//IM/3/HRC/FIFE//KRYMKI*6/4/GAZA/2*BOBIN//BUTTON/KENYA<br>73D2I1C/7/HRC/FIFE//IM/3/HRC/FIFE//KRYMKI*2/6/FRTR/4/RT//SPIJK/SHD/3/AKA/5/HRC/FIFE//<br>IM/3/HRC/FIFE//KRYMKI/8/CLMS/9/BW90	
TX158	HY344/3/HY320/NB402//RL4137/4/HY358/BW553	CANADA
TX159	69-1776/663//VEE	MEXICO
TX160	SABUF/4/GA/AE.SQUARROSA (224)//YACO/3/LHNKE/AE.SQUARROSA (205)//F27202	MEXICO
TX161	PASTOR/21/KAUZ*2/19/BJY/18/KT/6/H/4/3*HRC/FIFE//IM/3/HRC/FIFE//KRYMKI/5/FA/7/H/4/3*H<br>RC/FIFE//IM/3/HRC/FIFE//KRYMKI/5/FA/6/KT/8/KT/5/FRTR/4/RT//SPIJK/SHD/3/AKA/6/H/4/3*HR<br>C/FIFE//IM/3/HRC/FIFE//KRYMKI/5/FA/9/SWD/T.TIMOPHEEVII//RED EGYPTIAN(PI-	MEXICO

	170925)/KENYA BF4-3B.10.V.1/4/GB/KENIA RF 324/3/BTA/CHI//AMO25E/PELON 33C/16/LR64/15/FRTR/4/RT//SPIJK/SHD/3/AKA/5/RED EGYPTIAN(PI-170925)/KENYA BF4-3B.10.V.1/6/H/4/3*HRC/FIFE//IM/3/HRC/FIFE//KRYMKI/13/DRM/MI//KRYMKI/12/KRYMKI/7/FIFE/2*RIBA/6/FIFE/5/FIFE//FIFE/FIFE/4/FIFE/3/FIFE/FIFE//INDIAN G/8/DIEHL/MI/3/PP-AUS//FIFE/ETAWAH/9/ORO/10/DIEHL/MI/3/PP-AUS//FIFE/ETAWAH/11/ORO/8/KRYMKI/7/FIFE/2*RIBA/6/FIFE/5/FIFE//FIFE/FIFE/4/FIFE/3/FIFE/FIFE//INDIAN G/14/SWD/T.TIMOPHEEVII//RED EGYPTIAN(PI-170925)/KENYA BF4-3B.10.V.1/4/GB/KENIA RF 324/3/BTA/CHI//AMO25E/PELON 33C/17/LR64/15/H/4/3*HRC/FIFE//IM/3/HRC/FIFE//KRYMKI/5/FA/6/KT/7/FRTR/4/RT//SPIJK/SHD/3/AKA/13/DRM/MI//KRYMKI/12/KRYMKI/7/FIFE/2*RIBA/6/FIFE/5/FIFE//FIFE/FIFE/4/FIFE/3/FIFE/FIFE//INDIAN G/8/DIEHL/MI/3/PP-AUS//FIFE/ETAWAH/9/ORO/10/DIEHL/MI/3/PP-AUS//FIFE/ETAWAH/11/ORO/8/KRYMKI/7/FIFE/2*RIBA/6/FIFE/5/FIFE//FIFE/FIFE/4/FIFE/3/FIFE/FIFE//INDIAN G/14/2*H/4/3*HRC/FIFE//IM/3/HRC/FIFE//KRYMKI/5/FA/6/EGYPT NA101/7/SWD/T.TIMOPHEEVII/20/KAUZ	
TX162	LHNKE/AE.SQUARROSA (205)//KAUZ/3/SASIA	MEXICO
TX163	LHNKE/AE.SQUARROSA (205)//KAUZ/3/SASIA	MEXICO
TX164	LHNKE/AE.SQUARROSA (205)//WEAVER/3/ATTILA	MEXICO
TX165	PASTOR//TRAP/BOW/3/CHEN/AEGILOPS SQUARROSA (TAUS)//KAUZ	MEXICO
TX166	FILIN/IRENA/6/D65152/D6148//R143/3/ENTE/STR/4/AEGILOPS SQUARROSA (TAUS)/5/WEAVER	MEXICO
TX167	CHEN/AEGILOPS SQUARROSA (TAUS)//KAUZ/3/BABAX	MEXICO
TX168	CHEN/AEGILOPS SQUARROSA (TAUS)//KAUZ/3/CMH81.38/2*KAUZ	MEXICO
TX169	LHNKE/AE.SQUARROSA (205)//KAUZ/3/PASTOR	MEXICO
TX170	BUC/BJY//GA/AE.SQUARROSA (211)/3/BUC/BJY/4/PRINIA	MEXICO
TX171	LHNKE/AE.SQUARROSA (205)//KAUZ/3/ATTILA	MEXICO
TX172	FALKE//FALKE/BISU/3/CHEN/AEGILOPS SQUARROSA (TAUS)//KAUZ	MEXICO
TX173	CHIBIA/4/BUC/PAVON//LHNKE/AE.SQUARROSA (224)/3/2*WEAVER	MEXICO
TX174	RAC177/UNICULM492/15/H/4/3*HRC/FIFE//IM/3/HRC/FIFE//KRYMKI/5/FA/6/KT/7/FRTR/4/RT//SPIJK/SHD/3/AKA/13/DRM/MI//KRYMKI/12/KRYMKI/7/FIFE/2*RIBA/6/FIFE/5/FIFE//FIFE/FIFE/4/FIFE/3/FIFE/FIFE//INDIAN G/8/DIEHL/MI/3/PP-AUS//FIFE/ETAWAH/9/ORO/10/DIEHL/MI/3/PP-AUS//FIFE/ETAWAH/11/ORO/8/KRYMKI/7/FIFE/2*RIBA/6/FIFE/5/FIFE//FIFE/FIFE/4/FIFE/3/FIFE/FIFE//INDIAN G/14/GB/3/RAPR//CLEVARD/SANDS/4/KENYA C6042/5/DIRK 48	
TX175	3AG3/4*CNDR/3/TG/CNDR//CNDR	
TX176	GHURKA/RANEE/4/DU TOIT/3/DU TOIT//RYMER/MAFFRA/5/GB/15/FRTR/4/RT//SPIJK/SHD/3/AKA/5/RED EGYPTIAN(PI-170925)/KENYA BF4-3B.10.V.1/6/H/4/3*HRC/FIFE//IM/3/HRC/FIFE//KRYMKI/13/DRM/MI//KRYMKI/12/KRYMKI/7/FIFE/2*RIBA/6/FIFE/5/FIFE//FIFE/FIFE/4/FIFE/3/FIFE/FIFE//INDIAN G/8/DIEHL/MI/3/PP-AUS//FIFE/ETAWAH/9/ORO/10/DIEHL/MI/3/PP-AUS//FIFE/ETAWAH/11/ORO/8/KRYMKI/7/FIFE/2*RIBA/6/FIFE/5/FIFE//FIFE/FIFE/4/FIFE/3/FIFE/FIFE//INDIAN G/14/SWD/T.TIMOPHEEVII//RED EGYPTIAN(PI-170925)/KENYA BF4-3B.10.V.1/4/GB/KENIA RF 324/3/BTA/CHI//AMO25E/PELON 33C/17/GM/16/LR64/15/H/4/3*HRC/FIFE//IM/3/HRC/FIFE//KRYMKI/5/FA/6/KT/7/FRTR/4/RT//SPIJK/SHD/3/AKA/13/DRM/MI//KRYMKI/12/KRYMKI/7/FIFE/2*RIBA/6/FIFE/5/FIFE//FIFE/FIFE/4/FIFE/3/FIFE/FIFE//INDIAN G/8/DIEHL/MI/3/PP-AUS//FIFE/ETAWAH/9/ORO/10/DIEHL/MI/3/PP-AUS//FIFE/ETAWAH/11/ORO/8/KRYMKI/7/FIFE/2*RIBA/6/FIFE/5/FIFE//FIFE/FIFE/4/FIFE/3/FIFE/FIFE//INDIAN G/14/2*H/4/3*HRC/FIFE//IM/3/HRC/FIFE//KRYMKI/5/FA/6/EGYPT NA101/7/SWD/T.TIMOPHEEVII	
TX177	CS/AGEL(2N=14)//4*CNDR/3/TG/CNDR//CNDR	
TX178	HAHN*2/PRL	MEXICO
TX179	VS73.600/18/CNO/15/FRTR/4/RT//SPIJK/SHD/3/AKA/5/RED EGYPTIAN(PI-170925)/KENYA BF4-3B.10.V.1/6/H/4/3*HRC/FIFE//IM/3/HRC/FIFE//KRYMKI/13/DRM/MI//KRYMKI/12/KRYMKI/7/FIFE/2*RIBA/6/FIFE/5/FIFE//FIFE/FIFE/4/FIFE/3/FIFE/FIFE//INDIAN G/8/DIEHL/MI/3/PP-AUS//FIFE/ETAWAH/9/ORO/10/DIEHL/MI/3/PP-AUS//FIFE/ETAWAH/11/ORO/8/KRYMKI/7/FIFE/2*RIBA/6/FIFE/5/FIFE//FIFE/FIFE/4/FIFE/3/FIFE/FIFE//INDIAN G/14/SWD/T.TIMOPHEEVII//RED EGYPTIAN(PI-170925)/KENYA BF4-3B.10.V.1/4/GB/KENIA RF 324/3/BTA/CHI//AMO25E/PELON 33C/16/FRTR/4/RT//SPIJK/SHD/3/AKA/5/RED EGYPTIAN(PI-170925)/KENYA BF4-3B.10.V.1/6/H/4/3*HRC/FIFE//IM/3/HRC/FIFE//KRYMKI/13/DRM/MI//KRYMKI/12/KRYMKI/7/FIFE/2*RIBA/6/FIFE/5/FIFE//FIFE/FIFE/4/FIFE/3/FIFE/FIFE//INDIAN G/8/DIEHL/MI/3/PP-AUS//FIFE/ETAWAH/9/ORO/10/DIEHL/MI/3/PP-AUS//FIFE/ETAWAH/11/ORO/8/KRYMKI/7/FIFE/2*RIBA/6/FIFE/5/FIFE//FIFE/FIFE/4/FIFE/3/FIFE/FIFE//INDIAN G/14/SWD/T.TIMOPHEEVII//RED EGYPTIAN(PI-170925)/KENYA BF4-3B.10.V.1/4/GB/KENIA RF 324/3/BTA/CHI//AMO25E/PELON 33C/15/BB/17/PCI/19/BOW/11/M2A/10/KT/6/H/4/3*HRC/FIFE//IM/3/HRC/FIFE//KRYMKI/5/FA/7/RED EGYPTIAN(PI-170925)/KENYA BF4-3B.10.V.1/5/H/4/3*HRC/FIFE//IM/3/HRC/FIFE//KRYMKI/8/PP-AUS//FIFE/ETAWAH/3/KENYA 2/ENGLEDOWS/4/GB/KENIA RF 324/3/BTA/CHI//AMO25E/PELON 33C/9/HBR-DU/6/CHIDDAM	MEXICO

	D´AUTOMNE A EPI R/PRINCE ALBERT/3/NOE//BLE SEIGLE/HICKLING´S PROLIFIC/4/CHIDDAM BLANC/BLE SEIGLE//NOE/BLE SEIGLE/5/FIFE	
TX180	CHIL/2*STAR	MEXICO
TX181	BUC/PAVON	MEXICO
TX182	KT/6/H/4/3*HRC/FIFE//IM/3/HRC/FIFE//KRYMKI/5/FA/7/H/4/3*HRC/FIFE//IM/3/HRC/FIFE//KRYMKI/5/FA/6/KT/8/KT/5/FRTR/4/RT//SPIJK/SHD/3/AKA/6/H/4/3*HRC/FIFE//IM/3/HRC/FIFE//KRYMKI/5/FA/9/SWD/T.TIMOPHEEVII//RED EGYPTIAN(PI-170925)/KENYA BF4-3B.10.V.1/4/GB/KENIA RF 324/3/BTA/CHI//AMO25E/PELON 33C/16/LR64/15/FRTR/4/RT//SPIJK/SHD/3/AKA/5/RED EGYPTIAN(PI-170925)/KENYA BF4-3B.10.V.1/6/H/4/3*HRC/FIFE//IM/3/HRC/FIFE//KRYMKI/13/DRM/MI//KRYMKI/12/KRYMKI/7/FIFE/2*RIBA/6/FIFE/5/FIFE//FIFE/FIFE/4/FIFE/3/FIFE/FIFE//INDIAN G/8/DIEHL/MI/3/PP-AUS//FIFE/ETAWAH/9/ORO/10/DIEHL/MI/3/PP-AUS//FIFE/ETAWAH/11/ORO/8/KRYMKI/7/FIFE/2*RIBA/6/FIFE/5/FIFE//FIFE/FIFE/4/FIFE/3/FIFE/FIFE//INDIAN G/14/SWD/T.TIMOPHEEVII//RED EGYPTIAN(PI-170925)/KENYA BF4-3B.10.V.1/4/GB/KENIA RF 324/3/BTA/CHI//AMO25E/PELON 33C/17/LR64/15/H/4/3*HRC/FIFE//IM/3/HRC/FIFE//KRYMKI/5/FA/6/KT/7/FRTR/4/RT//SPIJK/SHD/3/AKA/13/DRM/MI//KRYMKI/12/KRYMKI/7/FIFE/2*RIBA/6/FIFE/5/FIFE//FIFE/FIFE/4/FIFE/3/FIFE/FIFE//INDIAN G/8/DIEHL/MI/3/PP-AUS//FIFE/ETAWAH/9/ORO/10/DIEHL/MI/3/PP-AUS//FIFE/ETAWAH/11/ORO/8/KRYMKI/7/FIFE/2*RIBA/6/FIFE/5/FIFE//FIFE/FIFE/4/FIFE/3/FIFE/FIFE//INDIAN G/14/2*H/4/3*HRC/FIFE//IM/3/HRC/FIFE//KRYMKI/5/FA/6/EGYPT NA101/7/SWD/T.TIMOPHEEVII/18/BJY/19/VEE	MEXICO
TX183	TURACO/CHIL	MEXICO
TX184	ISWRN-297/16/H/4/3*HRC/FIFE//IM/3/HRC/FIFE//KRYMKI/5/FA/6/KT/7/FRTR/4/RT//SPIJK/SHD/3/AKA/13/DRM/MI//KRYMKI/12/KRYMKI/7/FIFE/2*RIBA/6/FIFE/5/FIFE//FIFE/FIFE/4/FIFE/3/FIFE/FIFE//INDIAN G/8/DIEHL/MI/3/PP-AUS//FIFE/ETAWAH/9/ORO/10/DIEHL/MI/3/PP-AUS//FIFE/ETAWAH/11/ORO/8/KRYMKI/7/FIFE/2*RIBA/6/FIFE/5/FIFE//FIFE/FIFE/4/FIFE/3/FIFE/FIFE//INDIAN G/14/2*H/4/3*HRC/FIFE//IM/3/HRC/FIFE//KRYMKI/5/FA/6/EGYPT NA101/7/SWD/T.TIMOPHEEVII/15/AN64A/17/PP-AUS//FIFE/ETAWAH*2/3/Y/KT	MEXICO
TX185	FRTR/4/RT//SPIJK/SHD/3/AKA/5/RED EGYPTIAN(PI-170925)/KENYA BF4-3B.10.V.1/6/H/4/3*HRC/FIFE//IM/3/HRC/FIFE//KRYMKI/13/DRM/MI//KRYMKI/12/KRYMKI/7/FIFE/2*RIBA/6/FIFE/5/FIFE//FIFE/FIFE/4/FIFE/3/FIFE/FIFE//INDIAN G/8/DIEHL/MI/3/PP-AUS//FIFE/ETAWAH/9/ORO/10/DIEHL/MI/3/PP-AUS//FIFE/ETAWAH/11/ORO/8/KRYMKI/7/FIFE/2*RIBA/6/FIFE/5/FIFE//FIFE/FIFE/4/FIFE/3/FIFE/FIFE//INDIAN G/14/SWD/T.TIMOPHEEVII//RED EGYPTIAN(PI-170925)/KENYA BF4-3B.10.V.1/4/GB/KENIA RF 324/3/BTA/CHI//AMO25E/PELON 33C/15/GENTIL ROSSO/4/RT//SPIJK/SHD/3/AKA/5/SAN GIOVANNI/4/RT//SPIJK/SHD/3/AKA/16/BB	TURKEY
TX186	BUC/BJY	MEXICO
TX187	LR64/15/H/4/3*HRC/FIFE//IM/3/HRC/FIFE//KRYMKI/5/FA/6/KT/7/FRTR/4/RT//SPIJK/SHD/3/AKA/13/DRM/MI//KRYMKI/12/KRYMKI/7/FIFE/2*RIBA/6/FIFE/5/FIFE//FIFE/FIFE/4/FIFE/3/FIFE/FIFE//INDIAN G/8/DIEHL/MI/3/PP-AUS//FIFE/ETAWAH/9/ORO/10/DIEHL/MI/3/PP-AUS//FIFE/ETAWAH/11/ORO/8/KRYMKI/7/FIFE/2*RIBA/6/FIFE/5/FIFE//FIFE/FIFE/4/FIFE/3/FIFE/FIFE//INDIAN G/14/2*H/4/3*HRC/FIFE//IM/3/HRC/FIFE//KRYMKI/5/FA/6/EGYPT NA101/7/SWD/T.TIMOPHEEVII	MEXICO
TX188	KT/6/H/4/3*HRC/FIFE//IM/3/HRC/FIFE//KRYMKI/5/FA/7/H/4/3*HRC/FIFE//IM/3/HRC/FIFE//KRYMKI/5/FA/6/KT/8/KT/5/FRTR/4/RT//SPIJK/SHD/3/AKA/6/H/4/3*HRC/FIFE//IM/3/HRC/FIFE//KRYMKI/5/FA/9/SWD/T.TIMOPHEEVII//RED EGYPTIAN(PI-170925)/KENYA BF4-3B.10.V.1/4/GB/KENIA RF 324/3/BTA/CHI//AMO25E/PELON 33C/16/LR64/15/FRTR/4/RT//SPIJK/SHD/3/AKA/5/RED EGYPTIAN(PI-170925)/KENYA BF4-3B.10.V.1/6/H/4/3*HRC/FIFE//IM/3/HRC/FIFE//KRYMKI/13/DRM/MI//KRYMKI/12/KRYMKI/7/FIFE/2*RIBA/6/FIFE/5/FIFE//FIFE/FIFE/4/FIFE/3/FIFE/FIFE//INDIAN G/8/DIEHL/MI/3/PP-AUS//FIFE/ETAWAH/9/ORO/10/DIEHL/MI/3/PP-AUS//FIFE/ETAWAH/11/ORO/8/KRYMKI/7/FIFE/2*RIBA/6/FIFE/5/FIFE//FIFE/FIFE/4/FIFE/3/FIFE/FIFE//INDIAN G/14/SWD/T.TIMOPHEEVII//RED EGYPTIAN(PI-170925)/KENYA BF4-3B.10.V.1/4/GB/KENIA RF 324/3/BTA/CHI//AMO25E/PELON 33C/17/LR64/15/H/4/3*HRC/FIFE//IM/3/HRC/FIFE//KRYMKI/5/FA/6/KT/7/FRTR/4/RT//SPIJK/SHD/3/AKA/13/DRM/MI//KRYMKI/12/KRYMKI/7/FIFE/2*RIBA/6/FIFE/5/FIFE//FIFE/FIFE/4/FIFE/3/FIFE/FIFE//INDIAN G/8/DIEHL/MI/3/PP-AUS//FIFE/ETAWAH/9/ORO/10/DIEHL/MI/3/PP-AUS//FIFE/ETAWAH/11/ORO/8/KRYMKI/7/FIFE/2*RIBA/6/FIFE/5/FIFE//FIFE/FIFE/4/FIFE/3/FIFE/FIFE//INDIAN G/14/2*H/4/3*HRC/FIFE//IM/3/HRC/FIFE//KRYMKI/5/FA/6/EGYPT NA101/7/SWD/T.TIMOPHEEVII	MEXICO
TX189	FRTR/4/RT//SPIJK/SHD/3/AKA/5/RED EGYPTIAN(PI-170925)/KENYA BF4-3B.10.V.1/6/H/4/3*HRC/FIFE//IM/3/HRC/FIFE//KRYMKI/13/DRM/MI//KRYMKI/12/KRYMKI/7/FIFE/2*RIBA/6/FIFE/5/FIFE//FIFE/FIFE/4/FIFE/3/FIFE/FIFE//INDIAN G/8/DIEHL/MI/3/PP-AUS//FIFE/ETAWAH/9/ORO/10/DIEHL/MI/3/PP-AUS//FIFE/ETAWAH/11/ORO/8/KRYMKI/7/FIFE/2*RIBA/6/FIFE/5/FIFE//FIFE/FIFE/4/FIFE/3/FIFE/FIFE//INDIAN G/14/SWD/T.TIMOPHEEVII//RED EGYPTIAN(PI-170925)/KENYA BF4-3B.10.V.1/4/GB/KENIA RF 324/3/BTA/CHI//AMO25E/PELON 33C	MEXICO

TX190	H/4/3*HRC/FIFE//IM/3/HRC/FIFE//KRYMKI/5/FA/13/DRM/MI//KRYMKI/12/KRYMKI/7/FIFE/2*RI<br>BA/6/FIFE/5/FIFE//FIFE/FIFE/4/FIFE/3/FIFE/FIFE//INDIAN G/8/DIEHL/MI/3/PP-<br>AUS//FIFE/ETAWAH/9/ORO/10/DIEHL/MI/3/PP-<br>AUS//FIFE/ETAWAH/11/ORO/8/KRYMKI/7/FIFE/2*RIBA/6/FIFE/5/FIFE//FIFE/FIFE/4/FIFE/3/FIFE/<br>FIFE//INDIAN G/14/RT//SPIJK/SHD/3/AKA*3/4/KENIA RF<br>324/15/2*RT//SPIJK/SHD/3/AKA*3/4/KENIA RF<br>324/8/H/4/3*HRC/FIFE//IM/3/HRC/FIFE//KRYMKI/5/FA/6/2*ME/7/SUPREZA//H/MI	MEXICO
TX191	FRTR/4/RT//SPIJK/SHD/3/AKA/5/RED EGYPTIAN(PI-170925)/KENYA BF4-<br>3B.10.V.1/6/H/4/3*HRC/FIFE//IM/3/HRC/FIFE//KRYMKI/13/DRM/MI//KRYMKI/12/KRYMKI/7/FIF<br>E/2*RIBA/6/FIFE/5/FIFE//FIFE/FIFE/4/FIFE/3/FIFE/FIFE//INDIAN G/8/DIEHL/MI/3/PP-<br>AUS//FIFE/ETAWAH/9/ORO/10/DIEHL/MI/3/PP-<br>AUS//FIFE/ETAWAH/11/ORO/8/KRYMKI/7/FIFE/2*RIBA/6/FIFE/5/FIFE//FIFE/FIFE/4/FIFE/3/FIFE/<br>FIFE//INDIAN G/14/SWD/T.TIMOPHEEVII//RED EGYPTIAN(PI-170925)/KENYA BF4-<br>3B.10.V.1/4/GB/KENIA RF 324/3/BTA/CHI//AMO25E/PELON 33C	MEXICO
TX192	CRIEWENER<br>104/CENT.PETKUSER/8/CLTA/5/FRTR/4/RT//SPIJK/SHD/3/AKA/6/UKA/7/KRYMKI//MI/MI/3/KL0<br>33/9/BEZ1/10/BUHO/16/FRTR/4/RT//SPIJK/SHD/3/AKA/5/RED EGYPTIAN(PI-170925)/KENYA<br>BF4-<br>3B.10.V.1/6/H/4/3*HRC/FIFE//IM/3/HRC/FIFE//KRYMKI/13/DRM/MI//KRYMKI/12/KRYMKI/7/FIF<br>E/2*RIBA/6/FIFE/5/FIFE//FIFE/FIFE/4/FIFE/3/FIFE/FIFE//INDIAN G/8/DIEHL/MI/3/PP-<br>AUS//FIFE/ETAWAH/9/ORO/10/DIEHL/MI/3/PP-<br>AUS//FIFE/ETAWAH/11/ORO/8/KRYMKI/7/FIFE/2*RIBA/6/FIFE/5/FIFE//FIFE/FIFE/4/FIFE/3/FIFE/<br>FIFE//INDIAN G/14/SWD/T.TIMOPHEEVII//RED EGYPTIAN(PI-170925)/KENYA BF4-<br>3B.10.V.1/4/GB/KENIA RF 324/3/BTA/CHI//AMO25E/PELON 33C/15/BB	MEXICO
TX193	OROFEN 60	CHILE
TX194	LR64/15/H/4/3*HRC/FIFE//IM/3/HRC/FIFE//KRYMKI/5/FA/6/KT/7/FRTR/4/RT//SPIJK/SHD/3/AKA/<br>13/DRM/MI//KRYMKI/12/KRYMKI/7/FIFE/2*RIBA/6/FIFE/5/FIFE//FIFE/FIFE/4/FIFE/3/FIFE/FIFE//<br>INDIAN G/8/DIEHL/MI/3/PP-AUS//FIFE/ETAWAH/9/ORO/10/DIEHL/MI/3/PP-<br>AUS//FIFE/ETAWAH/11/ORO/8/KRYMKI/7/FIFE/2*RIBA/6/FIFE/5/FIFE//FIFE/FIFE/4/FIFE/3/FIFE/<br>FIFE//INDIAN G/14/2*H/4/3*HRC/FIFE//IM/3/HRC/FIFE//KRYMKI/5/FA/6/EGYPT<br>NA101/7/SWD/T.TIMOPHEEVII/16/FRTR/4/RT//SPIJK/SHD/3/AKA/5/C.O./C.R./7/FRTR/4/RT//SPIJ<br>K/SHD/3/AKA/6/H/4/3*HRC/FIFE//IM/3/HRC/FIFE//KRYMKI/5/FA/8/FRTR/4/RT//SPIJK/SHD/3/AK<br>A/5/C.O./C.R./6/LD/FIFE//RIGA<br>M/7/KT/6/H/4/3*HRC/FIFE//IM/3/HRC/FIFE//KRYMKI/5/FA/17/CNO/15/FRTR/4/RT//SPIJK/SHD/3/<br>AKA/5/RED EGYPTIAN(PI-170925)/KENYA BF4-<br>3B.10.V.1/6/H/4/3*HRC/FIFE//IM/3/HRC/FIFE//KRYMKI/13/DRM/MI//KRYMKI/12/KRYMKI/7/FIF<br>E/2*RIBA/6/FIFE/5/FIFE//FIFE/FIFE/4/FIFE/3/FIFE/FIFE//INDIAN G/8/DIEHL/MI/3/PP-<br>AUS//FIFE/ETAWAH/9/ORO/10/DIEHL/MI/3/PP-<br>AUS//FIFE/ETAWAH/11/ORO/8/KRYMKI/7/FIFE/2*RIBA/6/FIFE/5/FIFE//FIFE/FIFE/4/FIFE/3/FIFE/<br>FIFE//INDIAN G/14/SWD/T.TIMOPHEEVII//RED EGYPTIAN(PI-170925)/KENYA BF4-<br>3B.10.V.1/4/GB/KENIA RF 324/3/BTA/CHI//AMO25E/PELON<br>33C/18/FRTR/4/RT//SPIJK/SHD/3/AKA/5/RED EGYPTIAN(PI-170925)/KENYA BF4-<br>3B.10.V.1/6/H/4/3*HRC/FIFE//IM/3/HRC/FIFE//KRYMKI/13/DRM/MI//KRYMKI/12/KRYMKI/7/FIF<br>E/2*RIBA/6/FIFE/5/FIFE//FIFE/FIFE/4/FIFE/3/FIFE/FIFE//INDIAN G/8/DIEHL/MI/3/PP-<br>AUS//FIFE/ETAWAH/9/ORO/10/DIEHL/MI/3/PP-<br>AUS//FIFE/ETAWAH/11/ORO/8/KRYMKI/7/FIFE/2*RIBA/6/FIFE/5/FIFE//FIFE/FIFE/4/FIFE/3/FIFE/<br>FIFE//INDIAN G/14/SWD/T.TIMOPHEEVII//RED EGYPTIAN(PI-170925)/KENYA BF4-<br>3B.10.V.1/4/GB/KENIA RF 324/3/BTA/CHI//AMO25E/PELON 33C/15/BB	MEXICO
TX195	CRIEWENER<br>104/CENT.PETKUSER/8/CLTA/5/FRTR/4/RT//SPIJK/SHD/3/AKA/6/UKA/7/KRYMKI//MI/MI/3/KL0<br>33/9/BEZ1/10/BUHO/16/FRTR/4/RT//SPIJK/SHD/3/AKA/5/RED EGYPTIAN(PI-170925)/KENYA<br>BF4-<br>3B.10.V.1/6/H/4/3*HRC/FIFE//IM/3/HRC/FIFE//KRYMKI/13/DRM/MI//KRYMKI/12/KRYMKI/7/FIF<br>E/2*RIBA/6/FIFE/5/FIFE//FIFE/FIFE/4/FIFE/3/FIFE/FIFE//INDIAN G/8/DIEHL/MI/3/PP-<br>AUS//FIFE/ETAWAH/9/ORO/10/DIEHL/MI/3/PP-<br>AUS//FIFE/ETAWAH/11/ORO/8/KRYMKI/7/FIFE/2*RIBA/6/FIFE/5/FIFE//FIFE/FIFE/4/FIFE/3/FIFE/<br>FIFE//INDIAN G/14/SWD/T.TIMOPHEEVII//RED EGYPTIAN(PI-170925)/KENYA BF4-<br>3B.10.V.1/4/GB/KENIA RF 324/3/BTA/CHI//AMO25E/PELON 33C/15/BB	MEXICO
TX196	LR64/15/H/4/3*HRC/FIFE//IM/3/HRC/FIFE//KRYMKI/5/FA/6/KT/7/FRTR/4/RT//SPIJK/SHD/3/AKA/<br>13/DRM/MI//KRYMKI/12/KRYMKI/7/FIFE/2*RIBA/6/FIFE/5/FIFE//FIFE/FIFE/4/FIFE/3/FIFE/FIFE//<br>INDIAN G/8/DIEHL/MI/3/PP-AUS//FIFE/ETAWAH/9/ORO/10/DIEHL/MI/3/PP-<br>AUS//FIFE/ETAWAH/11/ORO/8/KRYMKI/7/FIFE/2*RIBA/6/FIFE/5/FIFE//FIFE/FIFE/4/FIFE/3/FIFE/<br>FIFE//INDIAN G/14/2*H/4/3*HRC/FIFE//IM/3/HRC/FIFE//KRYMKI/5/FA/6/EGYPT<br>NA101/7/SWD/T.TIMOPHEEVII/16/FRTR/4/RT//SPIJK/SHD/3/AKA/5/C.O./C.R./7/FRTR/4/RT//SPIJ<br>K/SHD/3/AKA/6/H/4/3*HRC/FIFE//IM/3/HRC/FIFE//KRYMKI/5/FA/8/FRTR/4/RT//SPIJK/SHD/3/AK<br>A/5/C.O./C.R./6/LD/FIFE//RIGA M/7/KT/6/H/4/3*HRC/FIFE//IM/3/HRC/FIFE//KRYMKI/5/FA	MEXICO
TX197	H/4/3*HRC/FIFE//IM/3/HRC/FIFE//KRYMKI/5/FA/14/DRM/MI//KRYMKI/12/KRYMKI/7/FIFE/2*RI<br>BA/6/FIFE/5/FIFE//FIFE/FIFE/4/FIFE/3/FIFE/FIFE//INDIAN G/8/DIEHL/MI/3/PP-<br>AUS//FIFE/ETAWAH/9/ORO/10/DIEHL/MI/3/PP-	MEXICO

	AUS//FIFE/ETAWAH/11/ORO/8/KRYMKI/7/FIFE/2*RIBA/6/FIFE/5/FIFE//FIFE/FIFE/4/FIFE/3/FIFE/FIFE//INDIAN G/13/RT//SPIJK/SHD/3/AKA*3/4/KENIA RF 324/15/LR64	
TX198	LR64/15/H/4/3*HRC/FIFE//IM/3/HRC/FIFE//KRYMKI/5/FA/6/KT/7/FRTR/4/RT//SPIJK/SHD/3/AKA/13/DRM/MI//KRYMKI/12/KRYMKI/7/FIFE/2*RIBA/6/FIFE/5/FIFE//FIFE/FIFE/4/FIFE/3/FIFE/FIFE//INDIAN G/8/DIEHL/MI/3/PP-AUS//FIFE/ETAWAH/9/ORO/10/DIEHL/MI/3/PP-AUS//FIFE/ETAWAH/11/ORO/8/KRYMKI/7/FIFE/2*RIBA/6/FIFE/5/FIFE//FIFE/FIFE/4/FIFE/3/FIFE/FIFE//INDIAN G/14/2*H/4/3*HRC/FIFE//IM/3/HRC/FIFE//KRYMKI/5/FA/6/EGYPT NA101/7/SWD/T.TIMOPHEEVII/16/RL4220/17/FRTR/4/RT//SPIJK/SHD/3/AKA/5/RED EGYPTIAN(PI-170925)/KENYA BF4-3B.10.V.1/6/H/4/3*HRC/FIFE//IM/3/HRC/FIFE//KRYMKI/13/DRM/MI//KRYMKI/12/KRYMKI/7/FIFE/2*RIBA/6/FIFE/5/FIFE//FIFE/FIFE/4/FIFE/3/FIFE/FIFE//INDIAN G/8/DIEHL/MI/3/PP-AUS//FIFE/ETAWAH/9/ORO/10/DIEHL/MI/3/PP-AUS//FIFE/ETAWAH/11/ORO/8/KRYMKI/7/FIFE/2*RIBA/6/FIFE/5/FIFE//FIFE/FIFE/4/FIFE/3/FIFE/FIFE//INDIAN G/14/SWD/T.TIMOPHEEVII//RED EGYPTIAN(PI-170925)/KENYA BF4-3B.10.V.1/4/GB/KENIA RF 324/3/BTA/CHI//AMO25E/PELON 33C/18/BB	MEXICO
TX199	CNO/14/FRTR/4/RT//SPIJK/SHD/3/AKA/5/RED EGYPTIAN(PI-170925)/KENYA BF4-3B.10.V.1/6/H/4/3*HRC/FIFE//IM/3/HRC/FIFE//KRYMKI/13/DRM/MI//KRYMKI/12/KRYMKI/7/FIFE/2*RIBA/6/FIFE/5/FIFE//FIFE/FIFE/4/FIFE/3/FIFE/FIFE//INDIAN G/8/DIEHL/MI/3/PP-AUS//FIFE/ETAWAH/9/ORO/10/DIEHL/MI/3/PP-AUS//FIFE/ETAWAH/11/ORO/8/KRYMKI/7/FIFE/2*RIBA/6/FIFE/5/FIFE//FIFE/FIFE/4/FIFE/3/FIFE/FIFE//INDIAN G/16/CNO/15/FRTR/4/RT//SPIJK/SHD/3/AKA/5/RED EGYPTIAN(PI-170925)/KENYA BF4-3B.10.V.1/6/H/4/3*HRC/FIFE//IM/3/HRC/FIFE//KRYMKI/13/DRM/MI//KRYMKI/12/KRYMKI/7/FIFE/2*RIBA/6/FIFE/5/FIFE//FIFE/FIFE/4/FIFE/3/FIFE/FIFE//INDIAN G/8/DIEHL/MI/3/PP-AUS//FIFE/ETAWAH/9/ORO/10/DIEHL/MI/3/PP-AUS//FIFE/ETAWAH/11/ORO/8/KRYMKI/7/FIFE/2*RIBA/6/FIFE/5/FIFE//FIFE/FIFE/4/FIFE/3/FIFE/FIFE//INDIAN G/14/SWD/T.TIMOPHEEVII//RED EGYPTIAN(PI-170925)/KENYA BF4-3B.10.V.1/4/GB/KENIA RF 324/3/BTA/CHI//AMO25E/PELON 33C	MEXICO
TX200	KT/6/H/4/3*HRC/FIFE//IM/3/HRC/FIFE//KRYMKI/5/FA/7/H/4/3*HRC/FIFE//IM/3/HRC/FIFE//KRYMKI/5/FA/6/KT/8/KT/5/FRTR/4/RT//SPIJK/SHD/3/AKA/6/H/4/3*HRC/FIFE//IM/3/HRC/FIFE//KRYMKI/5/FA/9/SWD/T.TIMOPHEEVII//RED EGYPTIAN(PI-170925)/KENYA BF4-3B.10.V.1/4/GB/KENIA RF 324/3/BTA/CHI//AMO25E/PELON 33C/16/LR64/15/FRTR/4/RT//SPIJK/SHD/3/AKA/5/RED EGYPTIAN(PI-170925)/KENYA BF4-3B.10.V.1/6/H/4/3*HRC/FIFE//IM/3/HRC/FIFE//KRYMKI/13/DRM/MI//KRYMKI/12/KRYMKI/7/FIFE/2*RIBA/6/FIFE/5/FIFE//FIFE/FIFE/4/FIFE/3/FIFE/FIFE//INDIAN G/8/DIEHL/MI/3/PP-AUS//FIFE/ETAWAH/9/ORO/10/DIEHL/MI/3/PP-AUS//FIFE/ETAWAH/11/ORO/8/KRYMKI/7/FIFE/2*RIBA/6/FIFE/5/FIFE//FIFE/FIFE/4/FIFE/3/FIFE/FIFE//INDIAN G/14/SWD/T.TIMOPHEEVII//RED EGYPTIAN(PI-170925)/KENYA BF4-3B.10.V.1/4/GB/KENIA RF 324/3/BTA/CHI//AMO25E/PELON 33C/17/LR64/15/H/4/3*HRC/FIFE//IM/3/HRC/FIFE//KRYMKI/5/FA/6/KT/7/FRTR/4/RT//SPIJK/SHD/3/AKA/13/DRM/MI//KRYMKI/12/KRYMKI/7/FIFE/2*RIBA/6/FIFE/5/FIFE//FIFE/FIFE/4/FIFE/3/FIFE/FIFE//INDIAN G/8/DIEHL/MI/3/PP-AUS//FIFE/ETAWAH/9/ORO/10/DIEHL/MI/3/PP-AUS//FIFE/ETAWAH/11/ORO/8/KRYMKI/7/FIFE/2*RIBA/6/FIFE/5/FIFE//FIFE/FIFE/4/FIFE/3/FIFE/FIFE//INDIAN G/14/2*H/4/3*HRC/FIFE//IM/3/HRC/FIFE//KRYMKI/5/FA/6/EGYPT NA101/7/SWD/T.TIMOPHEEVII/18/BJY/19/VEE	MEXICO
TX201	H/4/3*HRC/FIFE//IM/3/HRC/FIFE//KRYMKI/5/FA/6/KT/7/FRTR/4/RT//SPIJK/SHD/3/AKA/13/DRM/MI//KRYMKI/12/KRYMKI/7/FIFE/2*RIBA/6/FIFE/5/FIFE//FIFE/FIFE/4/FIFE/3/FIFE/FIFE//INDIAN G/8/DIEHL/MI/3/PP-AUS//FIFE/ETAWAH/9/ORO/10/DIEHL/MI/3/PP-AUS//FIFE/ETAWAH/11/ORO/8/KRYMKI/7/FIFE/2*RIBA/6/FIFE/5/FIFE//FIFE/FIFE/4/FIFE/3/FIFE/FIFE//INDIAN G/14/2*H/4/3*HRC/FIFE//IM/3/HRC/FIFE//KRYMKI/5/FA/6/EGYPT NA101/7/SWD/T.TIMOPHEEVII/15/CNO/16/LR64/15/H/4/3*HRC/FIFE//IM/3/HRC/FIFE//KRYMKI/5/FA/6/KT/7/FRTR/4/RT//SPIJK/SHD/3/AKA/13/DRM/MI//KRYMKI/12/KRYMKI/7/FIFE/2*RIBA/6/FIFE/5/FIFE//FIFE/FIFE/4/FIFE/3/FIFE/FIFE//INDIAN G/8/DIEHL/MI/3/PP-AUS//FIFE/ETAWAH/9/ORO/10/DIEHL/MI/3/PP-AUS//FIFE/ETAWAH/11/ORO/8/KRYMKI/7/FIFE/2*RIBA/6/FIFE/5/FIFE//FIFE/FIFE/4/FIFE/3/FIFE/FIFE//INDIAN G/14/2*H/4/3*HRC/FIFE//IM/3/HRC/FIFE//KRYMKI/5/FA/6/EGYPT NA101/7/SWD/T.TIMOPHEEVII	
TX202	IAS58/18/FRTR/4/RT//SPIJK/SHD/3/AKA/5/RED EGYPTIAN(PI-170925)/KENYA BF4-3B.10.V.1/6/H/4/3*HRC/FIFE//IM/3/HRC/FIFE//KRYMKI/13/DRM/MI//KRYMKI/12/KRYMKI/7/FIFE/2*RIBA/6/FIFE/5/FIFE//FIFE/FIFE/4/FIFE/3/FIFE/FIFE//INDIAN G/8/DIEHL/MI/3/PP-AUS//FIFE/ETAWAH/9/ORO/10/DIEHL/MI/3/PP-AUS//FIFE/ETAWAH/11/ORO/8/KRYMKI/7/FIFE/2*RIBA/6/FIFE/5/FIFE//FIFE/FIFE/4/FIFE/3/FIFE/FIFE//INDIAN G/14/SWD/T.TIMOPHEEVII//RED EGYPTIAN(PI-170925)/KENYA BF4-3B.10.V.1/4/GB/KENIA RF 324/3/BTA/CHI//AMO25E/PELON 33C/15/BB/16/BB/17/ALD/19/BOW	MEXICO
TX203	MUNIA/KAUZ	MEXICO
TX204	FRTR/4/RT//SPIJK/SHD/3/AKA/5/RED EGYPTIAN(PI-170925)/KENYA BF4-3B.10.V.1/6/H/4/3*HRC/FIFE//IM/3/HRC/FIFE//KRYMKI/13/DRM/MI//KRYMKI/12/KRYMKI/7/FIFE/2*RIBA/6/FIFE/5/FIFE//FIFE/FIFE/4/FIFE/3/FIFE/FIFE//INDIAN G/8/DIEHL/MI/3/PP-AUS//FIFE/ETAWAH/9/ORO/10/DIEHL/MI/3/PP-AUS//FIFE/ETAWAH/11/ORO/8/KRYMKI/7/FIFE/2*RIBA/6/FIFE/5/FIFE//FIFE/FIFE/4/FIFE/3/FIFE/	MEXICO

	FIFE//INDIAN G/14/SWD/T.TIMOPHEEVII//RED EGYPTIAN(PI-170925)/KENYA BF4-3B.10.V.1/4/GB/KENIA RF 324/3/BTA/CHI//AMO25E/PELON 33C	
TX205	H/4/3*HRC/FIFE//IM/3/HRC/FIFE//KRYMKI/5/FA	MEXICO
TX206	H/4/3*HRC/FIFE//IM/3/HRC/FIFE//KRYMKI/5/FA/6/KT/7/FRTR/4/RT//SPIJK/SHD/3/AKA/13/DRM/MI//KRYMKI/12/KRYMKI/7/FIFE/2*RIBA/6/FIFE/5/FIFE//FIFE/FIFE/4/FIFE/3/FIFE/FIFE//INDIAN G/8/DIEHL/MI/3/PP-AUS//FIFE/ETAWAH/9/ORO/10/DIEHL/MI/3/PP-AUS//FIFE/ETAWAH/11/ORO/8/KRYMKI/7/FIFE/2*RIBA/6/FIFE/5/FIFE//FIFE/FIFE/4/FIFE/3/FIFE/FIFE//INDIAN G/14/2*H/4/3*HRC/FIFE//IM/3/HRC/FIFE//KRYMKI/5/FA/6/EGYPT NA101/7/SWD/T.TIMOPHEEVII/15/FRTR/4/RT//SPIJK/SHD/3/AKA/5/HRC/FIFE//IM/3/HRC/FIFE//KRYMKI/4/BTA/AMO44D//BTA/CHI/6/P4160/16/GB*2/3/BASIL/JONATHAN//C112364/5/RT//SPIJK/SHD/3/AKA/4/6*GB/15/FRTR/4/RT//SPIJK/SHD/3/AKA/5/RED EGYPTIAN(PI-170925)/KENYA BF4-3B.10.V.1/6/H/4/3*HRC/FIFE//IM/3/HRC/FIFE//KRYMKI/13/DRM/MI//KRYMKI/12/KRYMKI/7/FIFE/2*RIBA/6/FIFE/5/FIFE//FIFE/FIFE/4/FIFE/3/FIFE/FIFE//INDIAN G/8/DIEHL/MI/3/PP-AUS//FIFE/ETAWAH/9/ORO/10/DIEHL/MI/3/PP-AUS//FIFE/ETAWAH/11/ORO/8/KRYMKI/7/FIFE/2*RIBA/6/FIFE/5/FIFE//FIFE/FIFE/4/FIFE/3/FIFE/FIFE//INDIAN G/14/SWD/T.TIMOPHEEVII//RED EGYPTIAN(PI-170925)/KENYA BF4-3B.10.V.1/4/GB/KENIA RF 324/3/BTA/CHI//AMO25E/PELON 33C/17/CHA/18/BB/16/LR64/15/H/4/3*HRC/FIFE//IM/3/HRC/FIFE//KRYMKI/5/FA/6/KT/7/FRTR/4/RT//SPIJK/SHD/3/AKA/13/DRM/MI//KRYMKI/12/KRYMKI/7/FIFE/2*RIBA/6/FIFE/5/FIFE//FIFE/FIFE/4/FIFE/3/FIFE/FIFE//INDIAN G/8/DIEHL/MI/3/PP-AUS//FIFE/ETAWAH/9/ORO/10/DIEHL/MI/3/PP-AUS//FIFE/ETAWAH/11/ORO/8/KRYMKI/7/FIFE/2*RIBA/6/FIFE/5/FIFE//FIFE/FIFE/4/FIFE/3/FIFE/FIFE//INDIAN G/14/2*H/4/3*HRC/FIFE//IM/3/HRC/FIFE//KRYMKI/5/FA/6/EGYPT NA101/7/SWD/T.TIMOPHEEVII	MEXICO
TX207	CNO/15/FRTR/4/RT//SPIJK/SHD/3/AKA/5/RED EGYPTIAN(PI-170925)/KENYA BF4-3B.10.V.1/6/H/4/3*HRC/FIFE//IM/3/HRC/FIFE//KRYMKI/13/DRM/MI//KRYMKI/12/KRYMKI/7/FIFE/2*RIBA/6/FIFE/5/FIFE//FIFE/FIFE/4/FIFE/3/FIFE/FIFE//INDIAN G/8/DIEHL/MI/3/PP-AUS//FIFE/ETAWAH/9/ORO/10/DIEHL/MI/3/PP-AUS//FIFE/ETAWAH/11/ORO/8/KRYMKI/7/FIFE/2*RIBA/6/FIFE/5/FIFE//FIFE/FIFE/4/FIFE/3/FIFE/FIFE//INDIAN G/14/SWD/T.TIMOPHEEVII//RED EGYPTIAN(PI-170925)/KENYA BF4-3B.10.V.1/4/GB/KENIA RF 324/3/BTA/CHI//AMO25E/PELON 33C	MEXICO
TX208	FCH3/TRT//VEE	MEXICO
TX209	PFAU/VEE//VEE	MEXICO
TX210	BJY/18/KT/6/H/4/3*HRC/FIFE//IM/3/HRC/FIFE//KRYMKI/5/FA/7/H/4/3*HRC/FIFE//IM/3/HRC/FIFE//KRYMKI/5/FA/6/KT/8/KT/5/FRTR/4/RT//SPIJK/SHD/3/AKA/6/H/4/3*HRC/FIFE//IM/3/HRC/FIFE//KRYMKI/5/FA/9/SWD/T.TIMOPHEEVII//RED EGYPTIAN(PI-170925)/KENYA BF4-3B.10.V.1/4/GB/KENIA RF 324/3/BTA/CHI//AMO25E/PELON 33C/16/LR64/15/FRTR/4/RT//SPIJK/SHD/3/AKA/5/RED EGYPTIAN(PI-170925)/KENYA BF4-3B.10.V.1/6/H/4/3*HRC/FIFE//IM/3/HRC/FIFE//KRYMKI/13/DRM/MI//KRYMKI/12/KRYMKI/7/FIFE/2*RIBA/6/FIFE/5/FIFE//FIFE/FIFE/4/FIFE/3/FIFE/FIFE//INDIAN G/8/DIEHL/MI/3/PP-AUS//FIFE/ETAWAH/9/ORO/10/DIEHL/MI/3/PP-AUS//FIFE/ETAWAH/11/ORO/8/KRYMKI/7/FIFE/2*RIBA/6/FIFE/5/FIFE//FIFE/FIFE/4/FIFE/3/FIFE/FIFE//INDIAN G/14/SWD/T.TIMOPHEEVII//RED EGYPTIAN(PI-170925)/KENYA BF4-3B.10.V.1/4/GB/KENIA RF 324/3/BTA/CHI//AMO25E/PELON 33C/17/LR64/15/H/4/3*HRC/FIFE//IM/3/HRC/FIFE//KRYMKI/5/FA/6/KT/7/FRTR/4/RT//SPIJK/SHD/3/AKA/13/DRM/MI//KRYMKI/12/KRYMKI/7/FIFE/2*RIBA/6/FIFE/5/FIFE//FIFE/FIFE/4/FIFE/3/FIFE/FIFE//INDIAN G/8/DIEHL/MI/3/PP-AUS//FIFE/ETAWAH/9/ORO/10/DIEHL/MI/3/PP-AUS//FIFE/ETAWAH/11/ORO/8/KRYMKI/7/FIFE/2*RIBA/6/FIFE/5/FIFE//FIFE/FIFE/4/FIFE/3/FIFE/FIFE//INDIAN G/14/2*H/4/3*HRC/FIFE//IM/3/HRC/FIFE//KRYMKI/5/FA/6/EGYPT NA101/7/SWD/T.TIMOPHEEVII/19/PRL/BOW	MEXICO
TX211	CHIL/20/CNO/GLL/18/FRTR/4/RT//SPIJK/SHD/3/AKA/5/HRC/FIFE//IM/3/HRC/FIFE//KRYMKI/4/BTA/AMO44D//BTA/CHI/15/H/4/3*HRC/FIFE//IM/3/HRC/FIFE//KRYMKI/5/FA/6/KT/7/FRTR/4/RT//SPIJK/SHD/3/AKA/13/DRM/MI//KRYMKI/12/KRYMKI/7/FIFE/2*RIBA/6/FIFE/5/FIFE//FIFE/FIFE/4/FIFE/3/FIFE/FIFE//INDIAN G/8/DIEHL/MI/3/PP-AUS//FIFE/ETAWAH/9/ORO/10/DIEHL/MI/3/PP-AUS//FIFE/ETAWAH/11/ORO/8/KRYMKI/7/FIFE/2*RIBA/6/FIFE/5/FIFE//FIFE/FIFE/4/FIFE/3/FIFE/FIFE//INDIAN G/14/2*H/4/3*HRC/FIFE//IM/3/HRC/FIFE//KRYMKI/5/FA/6/EGYPT NA101/7/SWD/T.TIMOPHEEVII/16/PRESIDENTE PERON MAG/4/BTA/CHI//AMO25E/PELON 33C/3/BTA/17/BB/19/FRTR/4/RT//SPIJK/SHD/3/AKA/5/HRC/FIFE//IM/3/HRC/FIFE//KRYMKI/4/BTA/AMO44D//BTA/CHI/15/H/4/3*HRC/FIFE//IM/3/HRC/FIFE//KRYMKI/5/FA/6/KT/7/FRTR/4/RT//SPIJK/SHD/3/AKA/13/DRM/MI//KRYMKI/12/KRYMKI/7/FIFE/2*RIBA/6/FIFE/5/FIFE//FIFE/FIFE/4/FIFE/3/FIFE/FIFE//INDIAN G/8/DIEHL/MI/3/PP-AUS//FIFE/ETAWAH/9/ORO/10/DIEHL/MI/3/PP-AUS//FIFE/ETAWAH/11/ORO/8/KRYMKI/7/FIFE/2*RIBA/6/FIFE/5/FIFE//FIFE/FIFE/4/FIFE/3/FIFE/FIFE//INDIAN G/14/2*H/4/3*HRC/FIFE//IM/3/HRC/FIFE//KRYMKI/5/FA/6/EGYPT NA101/7/SWD/T.TIMOPHEEVII/16/FRTR/4/RT//SPIJK/SHD/3/AKA/5/HRC/FIFE//IM/3/HRC/FIFE//KRYMKI/4/BTA/AMO44D//BTA/CHI*2/7/KT/5/FRTR/4/RT//SPIJK/SHD/3/AKA/6/H/4/3*HRC/FIFE//IM/3/HRC/FIFE//KRYMKI/5/FA	MEXICO
TX212	ARGUS/4/6*HRC/FIFE//IM/3/HRC/FIFE//KRYMKI/17/5*H/4/3*HRC/FIFE//IM/3/HRC/FIFE//KRYMKI/5/FA/6/KT/7/FRTR/4/RT//SPIJK/SHD/3/AKA/13/DRM/MI//KRYMKI/12/KRYMKI/7/FIFE/2*RIB	MEXICO

	A/6/FIFE/5/FIFE//FIFE/FIFE/4/FIFE/3/FIFE/FIFE//INDIAN G/8/DIEHL/MI/3/PP-AUS//FIFE/ETAWAH/9/ORO/10/DIEHL/MI/3/PP-AUS//FIFE/ETAWAH/11/ORO/8/KRYMKI/7/FIFE/2*RIBA/6/FIFE/5/FIFE//FIFE/FIFE/4/FIFE/3/FIFE/FIFE//INDIAN G/14/2*H/4/3*HRC/FIFE//IM/3/HRC/FIFE//KRYMKI/5/FA/6/EGYPT NA101/7/SWD/T.TIMOPHEEVII/15/CNO/16/LR64/15/H/4/3*HRC/FIFE//IM/3/HRC/FIFE//KRYMKI/5/FA/6/KT/7/FRTR/4/RT//SPIJK/SHD/3/AKA/13/DRM/MI//KRYMKI/12/KRYMKI/7/FIFE/2*RIBA/6/FIFE/5/FIFE//FIFE/FIFE/4/FIFE/3/FIFE/FIFE//INDIAN G/8/DIEHL/MI/3/PP-AUS//FIFE/ETAWAH/9/ORO/10/DIEHL/MI/3/PP-AUS//FIFE/ETAWAH/11/ORO/8/KRYMKI/7/FIFE/2*RIBA/6/FIFE/5/FIFE//FIFE/FIFE/4/FIFE/3/FIFE/FIFE//INDIAN G/14/2*H/4/3*HRC/FIFE//IM/3/HRC/FIFE//KRYMKI/5/FA/6/EGYPT NA101/7/SWD/T.TIMOPHEEVII/18/2*VEE	
TX213	D67.2/PARANA 66.270//AE.SQUARROSA (320)/3/CUNNINGHAM	MEXICO
TX214	D67.2/PARANA 66.270//AE.SQUARROSA (320)/3/CUNNINGHAM	MEXICO
TX215	PARUS/20/CHEN/AE.SQ/19/2*BJY/18/KT/6/H/4/3*HRC/FIFE//IM/3/HRC/FIFE//KRYMKI/5/FA/7/H/4/3*HRC/FIFE//IM/3/HRC/FIFE//KRYMKI/5/FA/6/KT/8/KT/5/FRTR/4/RT//SPIJK/SHD/3/AKA/6/H/4/3*HRC/FIFE//IM/3/HRC/FIFE//KRYMKI/5/FA/9/SWD/T.TIMOPHEEVII//RED EGYPTIAN(PI-170925)/KENYA BF4-3B.10.V.1/4/GB/KENIA RF 324/3/BTA/CHI//AMO25E/PELON 33C/16/LR64/15/FRTR/4/RT//SPIJK/SHD/3/AKA/5/RED EGYPTIAN(PI-170925)/KENYA BF4-3B.10.V.1/6/H/4/3*HRC/FIFE//IM/3/HRC/FIFE//KRYMKI/13/DRM/MI//KRYMKI/12/KRYMKI/7/FIFE/2*RIBA/6/FIFE/5/FIFE//FIFE/FIFE/4/FIFE/3/FIFE/FIFE//INDIAN G/8/DIEHL/MI/3/PP-AUS//FIFE/ETAWAH/9/ORO/10/DIEHL/MI/3/PP-AUS//FIFE/ETAWAH/11/ORO/8/KRYMKI/7/FIFE/2*RIBA/6/FIFE/5/FIFE//FIFE/FIFE/4/FIFE/3/FIFE/FIFE//INDIAN G/14/SWD/T.TIMOPHEEVII//RED EGYPTIAN(PI-170925)/KENYA BF4-3B.10.V.1/4/GB/KENIA RF 324/3/BTA/CHI//AMO25E/PELON 33C/17/LR64/15/H/4/3*HRC/FIFE//IM/3/HRC/FIFE//KRYMKI/5/FA/6/KT/7/FRTR/4/RT//SPIJK/SHD/3/AKA/13/DRM/MI//KRYMKI/12/KRYMKI/7/FIFE/2*RIBA/6/FIFE/5/FIFE//FIFE/FIFE/4/FIFE/3/FIFE/FIFE//INDIAN G/8/DIEHL/MI/3/PP-AUS//FIFE/ETAWAH/9/ORO/10/DIEHL/MI/3/PP-AUS//FIFE/ETAWAH/11/ORO/8/KRYMKI/7/FIFE/2*RIBA/6/FIFE/5/FIFE//FIFE/FIFE/4/FIFE/3/FIFE/FIFE//INDIAN G/14/2*H/4/3*HRC/FIFE//IM/3/HRC/FIFE//KRYMKI/5/FA/6/EGYPT NA101/7/SWD/T.TIMOPHEEVII	MEXICO
TX216	ATTILA/3*KAUZ/20/LHNKE/AE.SQUARROSA (224)/19/BJY/18/KT/6/H/4/3*HRC/FIFE//IM/3/HRC/FIFE//KRYMKI/5/FA/7/H/4/3*HRC/FIFE//IM/3/HRC/FIFE//KRYMKI/5/FA/6/KT/8/KT/5/FRTR/4/RT//SPIJK/SHD/3/AKA/6/H/4/3*HRC/FIFE//IM/3/HRC/FIFE//KRYMKI/5/FA/9/SWD/T.TIMOPHEEVII//RED EGYPTIAN(PI-170925)/KENYA BF4-3B.10.V.1/4/GB/KENIA RF 324/3/BTA/CHI//AMO25E/PELON 33C/16/LR64/15/FRTR/4/RT//SPIJK/SHD/3/AKA/5/RED EGYPTIAN(PI-170925)/KENYA BF4-3B.10.V.1/6/H/4/3*HRC/FIFE//IM/3/HRC/FIFE//KRYMKI/13/DRM/MI//KRYMKI/12/KRYMKI/7/FIFE/2*RIBA/6/FIFE/5/FIFE//FIFE/FIFE/4/FIFE/3/FIFE/FIFE//INDIAN G/8/DIEHL/MI/3/PP-AUS//FIFE/ETAWAH/9/ORO/10/DIEHL/MI/3/PP-AUS//FIFE/ETAWAH/11/ORO/8/KRYMKI/7/FIFE/2*RIBA/6/FIFE/5/FIFE//FIFE/FIFE/4/FIFE/3/FIFE/FIFE//INDIAN G/14/SWD/T.TIMOPHEEVII//RED EGYPTIAN(PI-170925)/KENYA BF4-3B.10.V.1/4/GB/KENIA RF 324/3/BTA/CHI//AMO25E/PELON 33C/17/LR64/15/H/4/3*HRC/FIFE//IM/3/HRC/FIFE//KRYMKI/5/FA/6/KT/7/FRTR/4/RT//SPIJK/SHD/3/AKA/13/DRM/MI//KRYMKI/12/KRYMKI/7/FIFE/2*RIBA/6/FIFE/5/FIFE//FIFE/FIFE/4/FIFE/3/FIFE/FIFE//INDIAN G/8/DIEHL/MI/3/PP-AUS//FIFE/ETAWAH/9/ORO/10/DIEHL/MI/3/PP-AUS//FIFE/ETAWAH/11/ORO/8/KRYMKI/7/FIFE/2*RIBA/6/FIFE/5/FIFE//FIFE/FIFE/4/FIFE/3/FIFE/FIFE//INDIAN G/14/2*H/4/3*HRC/FIFE//IM/3/HRC/FIFE//KRYMKI/5/FA/6/EGYPT NA101/7/SWD/T.TIMOPHEEVII	MEXICO
TX217	YANAC/23/PRL/21/T.AEST/19/TP/17/CNO/16/LR64/15/H/4/3*HRC/FIFE//IM/3/HRC/FIFE//KRYMKI/5/FA/6/KT/7/FRTR/4/RT//SPIJK/SHD/3/AKA/13/DRM/MI//KRYMKI/12/KRYMKI/7/FIFE/2*RIBA/6/FIFE/5/FIFE//FIFE/FIFE/4/FIFE/3/FIFE/FIFE//INDIAN G/8/DIEHL/MI/3/PP-AUS//FIFE/ETAWAH/9/ORO/10/DIEHL/MI/3/PP-AUS//FIFE/ETAWAH/11/ORO/8/KRYMKI/7/FIFE/2*RIBA/6/FIFE/5/FIFE//FIFE/FIFE/4/FIFE/3/FIFE/FIFE//INDIAN G/14/2*H/4/3*HRC/FIFE//IM/3/HRC/FIFE//KRYMKI/5/FA/6/EGYPT NA101/7/SWD/T.TIMOPHEEVII/18/CNO/15/FRTR/4/RT//SPIJK/SHD/3/AKA/5/RED EGYPTIAN(PI-170925)/KENYA BF4-3B.10.V.1/6/H/4/3*HRC/FIFE//IM/3/HRC/FIFE//KRYMKI/13/DRM/MI//KRYMKI/12/KRYMKI/7/FIFE/2*RIBA/6/FIFE/5/FIFE//FIFE/FIFE/4/FIFE/3/FIFE/FIFE//INDIAN G/8/DIEHL/MI/3/PP-AUS//FIFE/ETAWAH/9/ORO/10/DIEHL/MI/3/PP-AUS//FIFE/ETAWAH/11/ORO/8/KRYMKI/7/FIFE/2*RIBA/6/FIFE/5/FIFE//FIFE/FIFE/4/FIFE/3/FIFE/FIFE//INDIAN G/14/SWD/T.TIMOPHEEVII//RED EGYPTIAN(PI-170925)/KENYA BF4-3B.10.V.1/4/GB/KENIA RF 324/3/BTA/CHI//AMO25E/PELON 33C/20/KT/6/H/4/3*HRC/FIFE//IM/3/HRC/FIFE//KRYMKI/5/FA/7/H/4/3*HRC/FIFE//IM/3/HRC/FIFE//KRYMKI/5/FA/6/KT/8/KT/5/FRTR/4/RT//SPIJK/SHD/3/AKA/6/H/4/3*HRC/FIFE//IM/3/HRC/FIFE//KRYMKI/5/FA/9/SWD/T.TIMOPHEEVII//RED EGYPTIAN(PI-170925)/KENYA BF4-3B.10.V.1/4/GB/KENIA RF 324/3/BTA/CHI//AMO25E/PELON 33C/16/LR64/15/FRTR/4/RT//SPIJK/SHD/3/AKA/5/RED EGYPTIAN(PI-170925)/KENYA BF4-3B.10.V.1/6/H/4/3*HRC/FIFE//IM/3/HRC/FIFE//KRYMKI/13/DRM/MI//KRYMKI/12/KRYMKI/7/FIFE/2*RIBA/6/FIFE/5/FIFE//FIFE/FIFE/4/FIFE/3/FIFE/FIFE//INDIAN G/8/DIEHL/MI/3/PP-AUS//FIFE/ETAWAH/9/ORO/10/DIEHL/MI/3/PP-	MEXICO

	AUS//FIFE/ETAWAH/11/ORO/8/KRYMKI/7/FIFE/2*RIBA/6/FIFE/5/FIFE//FIFE/FIFE/4/FIFE/3/FIFE/<br>FIFE//INDIAN G/14/SWD/T.TIMOPHEEVII//RED EGYPTIAN(PI-170925)/KENYA BF4-<br>3B.10.V.1/4/GB/KENIA RF 324/3/BTA/CHI//AMO25E/PELON<br>33C/17/LR64/15/H/4/3*HRC/FIFE//IM/3/HRC/FIFE//KRYMKI/5/FA/6/KT/7/FRTR/4/RT//SPIJK/SHD/<br>3/AKA/13/DRM/MI//KRYMKI/12/KRYMKI/7/FIFE/2*RIBA/6/FIFE/5/FIFE//FIFE/FIFE/4/FIFE/3/FIF<br>E/FIFE//INDIAN G/8/DIEHL/MI/3/PP-AUS//FIFE/ETAWAH/9/ORO/10/DIEHL/MI/3/PP-<br>AUS//FIFE/ETAWAH/11/ORO/8/KRYMKI/7/FIFE/2*RIBA/6/FIFE/5/FIFE//FIFE/FIFE/4/FIFE/3/FIFE/<br>FIFE//INDIAN G/14/2*H/4/3*HRC/FIFE//IM/3/HRC/FIFE//KRYMKI/5/FA/6/EGYPT<br>NA101/7/SWD/T.TIMOPHEEVII/22/TTM/VEE/24/LHNKE/AE.SQUARROSA<br>(224)/19/BJY/18/KT/6/H/4/3*HRC/FIFE//IM/3/HRC/FIFE//KRYMKI/5/FA/7/H/4/3*HRC/FIFE//IM/3/H<br>RC/FIFE//KRYMKI/5/FA/6/KT/8/KT/5/FRTR/4/RT//SPIJK/SHD/3/AKA/6/H/4/3*HRC/FIFE//IM/3/HR<br>C/FIFE//KRYMKI/5/FA/9/SWD/T.TIMOPHEEVII//RED EGYPTIAN(PI-170925)/KENYA BF4-<br>3B.10.V.1/4/GB/KENIA RF 324/3/BTA/CHI//AMO25E/PELON<br>33C/16/LR64/15/FRTR/4/RT//SPIJK/SHD/3/AKA/5/RED EGYPTIAN(PI-170925)/KENYA BF4-<br>3B.10.V.1/6/H/4/3*HRC/FIFE//IM/3/HRC/FIFE//KRYMKI/13/DRM/MI//KRYMKI/12/KRYMKI/7/FIF<br>E/2*RIBA/6/FIFE/5/FIFE//FIFE/FIFE/4/FIFE/3/FIFE/FIFE//INDIAN G/8/DIEHL/MI/3/PP-<br>AUS//FIFE/ETAWAH/9/ORO/10/DIEHL/MI/3/PP-<br>AUS//FIFE/ETAWAH/11/ORO/8/KRYMKI/7/FIFE/2*RIBA/6/FIFE/5/FIFE//FIFE/FIFE/4/FIFE/3/FIFE/<br>FIFE//INDIAN G/14/SWD/T.TIMOPHEEVII//RED EGYPTIAN(PI-170925)/KENYA BF4-<br>3B.10.V.1/4/GB/KENIA RF 324/3/BTA/CHI//AMO25E/PELON<br>33C/17/LR64/15/H/4/3*HRC/FIFE//IM/3/HRC/FIFE//KRYMKI/5/FA/6/KT/7/FRTR/4/RT//SPIJK/SHD/<br>3/AKA/13/DRM/MI//KRYMKI/12/KRYMKI/7/FIFE/2*RIBA/6/FIFE/5/FIFE//FIFE/FIFE/4/FIFE/3/FIF<br>E/FIFE//INDIAN G/8/DIEHL/MI/3/PP-AUS//FIFE/ETAWAH/9/ORO/10/DIEHL/MI/3/PP-<br>AUS//FIFE/ETAWAH/11/ORO/8/KRYMKI/7/FIFE/2*RIBA/6/FIFE/5/FIFE//FIFE/FIFE/4/FIFE/3/FIFE/<br>FIFE//INDIAN G/14/2*H/4/3*HRC/FIFE//IM/3/HRC/FIFE//KRYMKI/5/FA/6/EGYPT<br>NA101/7/SWD/T.TIMOPHEEVII	
TX218	FGO/USA2111//AE.SQUARROSA<br>(658)/23/PRL/21/T.AEST/19/TP/17/CNO/16/LR64/15/H/4/3*HRC/FIFE//IM/3/HRC/FIFE//KRYMKI/5/<br>FA/6/KT/7/FRTR/4/RT//SPIJK/SHD/3/AKA/13/DRM/MI//KRYMKI/12/KRYMKI/7/FIFE/2*RIBA/6/FI<br>FE/5/FIFE//FIFE/FIFE/4/FIFE/3/FIFE/FIFE//INDIAN G/8/DIEHL/MI/3/PP-<br>AUS//FIFE/ETAWAH/9/ORO/10/DIEHL/MI/3/PP-<br>AUS//FIFE/ETAWAH/11/ORO/8/KRYMKI/7/FIFE/2*RIBA/6/FIFE/5/FIFE//FIFE/FIFE/4/FIFE/3/FIFE/<br>FIFE//INDIAN G/14/2*H/4/3*HRC/FIFE//IM/3/HRC/FIFE//KRYMKI/5/FA/6/EGYPT<br>NA101/7/SWD/T.TIMOPHEEVII/18/CNO/15/FRTR/4/RT//SPIJK/SHD/3/AKA/5/RED EGYPTIAN(PI-<br>170925)/KENYA BF4-<br>3B.10.V.1/6/H/4/3*HRC/FIFE//IM/3/HRC/FIFE//KRYMKI/13/DRM/MI//KRYMKI/12/KRYMKI/7/FIF<br>E/2*RIBA/6/FIFE/5/FIFE//FIFE/FIFE/4/FIFE/3/FIFE/FIFE//INDIAN G/8/DIEHL/MI/3/PP-<br>AUS//FIFE/ETAWAH/9/ORO/10/DIEHL/MI/3/PP-<br>AUS//FIFE/ETAWAH/11/ORO/8/KRYMKI/7/FIFE/2*RIBA/6/FIFE/5/FIFE//FIFE/FIFE/4/FIFE/3/FIFE/<br>FIFE//INDIAN G/14/SWD/T.TIMOPHEEVII//RED EGYPTIAN(PI-170925)/KENYA BF4-<br>3B.10.V.1/4/GB/KENIA RF 324/3/BTA/CHI//AMO25E/PELON<br>33C/20/KT/6/H/4/3*HRC/FIFE//IM/3/HRC/FIFE//KRYMKI/5/FA/7/H/4/3*HRC/FIFE//IM/3/HRC/FIFE<br>//KRYMKI/5/FA/6/KT/8/KT/5/FRTR/4/RT//SPIJK/SHD/3/AKA/6/H/4/3*HRC/FIFE//IM/3/HRC/FIFE//<br>KRYMKI/5/FA/9/SWD/T.TIMOPHEEVII//RED EGYPTIAN(PI-170925)/KENYA BF4-<br>3B.10.V.1/4/GB/KENIA RF 324/3/BTA/CHI//AMO25E/PELON<br>33C/16/LR64/15/FRTR/4/RT//SPIJK/SHD/3/AKA/5/RED EGYPTIAN(PI-170925)/KENYA BF4-<br>3B.10.V.1/6/H/4/3*HRC/FIFE//IM/3/HRC/FIFE//KRYMKI/13/DRM/MI//KRYMKI/12/KRYMKI/7/FIF<br>E/2*RIBA/6/FIFE/5/FIFE//FIFE/FIFE/4/FIFE/3/FIFE/FIFE//INDIAN G/8/DIEHL/MI/3/PP-<br>AUS//FIFE/ETAWAH/9/ORO/10/DIEHL/MI/3/PP-<br>AUS//FIFE/ETAWAH/11/ORO/8/KRYMKI/7/FIFE/2*RIBA/6/FIFE/5/FIFE//FIFE/FIFE/4/FIFE/3/FIFE/<br>FIFE//INDIAN G/14/SWD/T.TIMOPHEEVII//RED EGYPTIAN(PI-170925)/KENYA BF4-<br>3B.10.V.1/4/GB/KENIA RF 324/3/BTA/CHI//AMO25E/PELON<br>33C/17/LR64/15/H/4/3*HRC/FIFE//IM/3/HRC/FIFE//KRYMKI/5/FA/6/KT/7/FRTR/4/RT//SPIJK/SHD/<br>3/AKA/13/DRM/MI//KRYMKI/12/KRYMKI/7/FIFE/2*RIBA/6/FIFE/5/FIFE//FIFE/FIFE/4/FIFE/3/FIF<br>E/FIFE//INDIAN G/8/DIEHL/MI/3/PP-AUS//FIFE/ETAWAH/9/ORO/10/DIEHL/MI/3/PP-<br>AUS//FIFE/ETAWAH/11/ORO/8/KRYMKI/7/FIFE/2*RIBA/6/FIFE/5/FIFE//FIFE/FIFE/4/FIFE/3/FIFE/<br>FIFE//INDIAN G/14/2*H/4/3*HRC/FIFE//IM/3/HRC/FIFE//KRYMKI/5/FA/6/EGYPT<br>NA101/7/SWD/T.TIMOPHEEVII/22/TTM/VEE/24/ATTILA	MEXICO
TX219	LHNKE/AE.SQUARROSA (205)//KAUZ/3/DHARWAR DRY/4/WBLL1	MEXICO
TX220	AUS 4930.7/2*PASTOR	MEXICO
TX221	T.TAU.83.2.29/23/PRL/21/T.AEST/19/TP/17/CNO/16/LR64/15/H/4/3*HRC/FIFE//IM/3/HRC/FIFE//K<br>RYMKI/5/FA/6/KT/7/FRTR/4/RT//SPIJK/SHD/3/AKA/13/DRM/MI//KRYMKI/12/KRYMKI/7/FIFE/2*<br>RIBA/6/FIFE/5/FIFE//FIFE/FIFE/4/FIFE/3/FIFE/FIFE//INDIAN G/8/DIEHL/MI/3/PP-<br>AUS//FIFE/ETAWAH/9/ORO/10/DIEHL/MI/3/PP-<br>AUS//FIFE/ETAWAH/11/ORO/8/KRYMKI/7/FIFE/2*RIBA/6/FIFE/5/FIFE//FIFE/FIFE/4/FIFE/3/FIFE/<br>FIFE//INDIAN G/14/2*H/4/3*HRC/FIFE//IM/3/HRC/FIFE//KRYMKI/5/FA/6/EGYPT<br>NA101/7/SWD/T.TIMOPHEEVII/18/CNO/15/FRTR/4/RT//SPIJK/SHD/3/AKA/5/RED EGYPTIAN(PI-<br>170925)/KENYA BF4-<br>3B.10.V.1/6/H/4/3*HRC/FIFE//IM/3/HRC/FIFE//KRYMKI/13/DRM/MI//KRYMKI/12/KRYMKI/7/FIF<br>E/2*RIBA/6/FIFE/5/FIFE//FIFE/FIFE/4/FIFE/3/FIFE/FIFE//INDIAN G/8/DIEHL/MI/3/PP-	MEXICO

	AUS//FIFE/ETAWAH/9/ORO/10/DIEHL/MI/3/PP-<br>AUS//FIFE/ETAWAH/11/ORO/8/KRYMKI/7/FIFE/2*RIBA/6/FIFE/5/FIFE//FIFE/FIFE/4/FIFE/3/FIFE/<br>FIFE//INDIAN G/14/SWD/T.TIMOPHEEVII//RED EGYPTIAN(PI-170925)/KENYA BF4-<br>3B.10.V.1/4/GB/KENIA RF 324/3/BTA/CHI//AMO25E/PELON<br>33C/20/KT/6/H/4/3*HRC/FIFE//IM/3/HRC/FIFE//KRYMKI/5/FA/7/H/4/3*HRC/FIFE//IM/3/HRC/FIFE<br>//KRYMKI/5/FA/6/KT/8/KT/5/FRTR/4/RT//SPIJK/SHD/3/AKA/6/H/4/3*HRC/FIFE//IM/3/HRC/FIFE//<br>KRYMKI/5/FA/9/SWD/T.TIMOPHEEVII//RED EGYPTIAN(PI-170925)/KENYA BF4-<br>3B.10.V.1/4/GB/KENIA RF 324/3/BTA/CHI//AMO25E/PELON<br>33C/16/LR64/15/FRTR/4/RT//SPIJK/SHD/3/AKA/5/RED EGYPTIAN(PI-170925)/KENYA BF4-<br>3B.10.V.1/6/H/4/3*HRC/FIFE//IM/3/HRC/FIFE//KRYMKI/13/DRM/MI//KRYMKI/12/KRYMKI/7/FIF<br>E/2*RIBA/6/FIFE/5/FIFE//FIFE/FIFE/4/FIFE/3/FIFE/FIFE//INDIAN G/8/DIEHL/MI/3/PP-<br>AUS//FIFE/ETAWAH/9/ORO/10/DIEHL/MI/3/PP-<br>AUS//FIFE/ETAWAH/11/ORO/8/KRYMKI/7/FIFE/2*RIBA/6/FIFE/5/FIFE//FIFE/FIFE/4/FIFE/3/FIFE/<br>FIFE//INDIAN G/14/SWD/T.TIMOPHEEVII//RED EGYPTIAN(PI-170925)/KENYA BF4-<br>3B.10.V.1/4/GB/KENIA RF 324/3/BTA/CHI//AMO25E/PELON<br>33C/17/LR64/15/H/4/3*HRC/FIFE//IM/3/HRC/FIFE//KRYMKI/5/FA/6/KT/7/FRTR/4/RT//SPIJK/SHD/<br>3/AKA/13/DRM/MI//KRYMKI/12/KRYMKI/7/FIFE/2*RIBA/6/FIFE/5/FIFE//FIFE/FIFE/4/FIFE/3/FIF<br>E/FIFE//INDIAN G/8/DIEHL/MI/3/PP-AUS//FIFE/ETAWAH/9/ORO/10/DIEHL/MI/3/PP-<br>AUS//FIFE/ETAWAH/11/ORO/8/KRYMKI/7/FIFE/2*RIBA/6/FIFE/5/FIFE//FIFE/FIFE/4/FIFE/3/FIFE/<br>FIFE//INDIAN G/14/2*H/4/3*HRC/FIFE//IM/3/HRC/FIFE//KRYMKI/5/FA/6/EGYPT<br>NA101/7/SWD/T.TIMOPHEEVII/22/TTM/VEE/24/LHNKE/AE.SQUARROSA<br>(224)/19/BJY/18/KT/6/H/4/3*HRC/FIFE//IM/3/HRC/FIFE//KRYMKI/5/FA/7/H/4/3*HRC/FIFE//IM/3/H<br>RC/FIFE//KRYMKI/5/FA/6/KT/8/KT/5/FRTR/4/RT//SPIJK/SHD/3/AKA/6/H/4/3*HRC/FIFE//IM/3/HR<br>C/FIFE//KRYMKI/5/FA/9/SWD/T.TIMOPHEEVII//RED EGYPTIAN(PI-170925)/KENYA BF4-<br>3B.10.V.1/4/GB/KENIA RF 324/3/BTA/CHI//AMO25E/PELON<br>33C/16/LR64/15/FRTR/4/RT//SPIJK/SHD/3/AKA/5/RED EGYPTIAN(PI-170925)/KENYA BF4-<br>3B.10.V.1/6/H/4/3*HRC/FIFE//IM/3/HRC/FIFE//KRYMKI/13/DRM/MI//KRYMKI/12/KRYMKI/7/FIF<br>E/2*RIBA/6/FIFE/5/FIFE//FIFE/FIFE/4/FIFE/3/FIFE/FIFE//INDIAN G/8/DIEHL/MI/3/PP-<br>AUS//FIFE/ETAWAH/9/ORO/10/DIEHL/MI/3/PP-<br>AUS//FIFE/ETAWAH/11/ORO/8/KRYMKI/7/FIFE/2*RIBA/6/FIFE/5/FIFE//FIFE/FIFE/4/FIFE/3/FIFE/<br>FIFE//INDIAN G/14/SWD/T.TIMOPHEEVII//RED EGYPTIAN(PI-170925)/KENYA BF4-<br>3B.10.V.1/4/GB/KENIA RF 324/3/BTA/CHI//AMO25E/PELON<br>33C/17/LR64/15/H/4/3*HRC/FIFE//IM/3/HRC/FIFE//KRYMKI/5/FA/6/KT/7/FRTR/4/RT//SPIJK/SHD/<br>3/AKA/13/DRM/MI//KRYMKI/12/KRYMKI/7/FIFE/2*RIBA/6/FIFE/5/FIFE//FIFE/FIFE/4/FIFE/3/FIF<br>E/FIFE//INDIAN G/8/DIEHL/MI/3/PP-AUS//FIFE/ETAWAH/9/ORO/10/DIEHL/MI/3/PP-<br>AUS//FIFE/ETAWAH/11/ORO/8/KRYMKI/7/FIFE/2*RIBA/6/FIFE/5/FIFE//FIFE/FIFE/4/FIFE/3/FIFE/<br>FIFE//INDIAN G/14/2*H/4/3*HRC/FIFE//IM/3/HRC/FIFE//KRYMKI/5/FA/6/EGYPT<br>NA101/7/SWD/T.TIMOPHEEVII	
TX222	CETA/AE.SQUARROSA (327)//2*SUNLIN	MEXICO
TX223	CETA/AE.SQUARROSA (327)//2*SUNLIN	MEXICO
TX224	T.DICOCCON PI225332/AE.SQUARROSA (895)//WBLL1/3/2*WBLL1	MEXICO
TX225	T.DICOCCON PI94625/AE.SQUARROSA (372)//3*PASTOR	MEXICO
TX226	T.DICOCCON PI225332/AE.SQUARROSA (895)//WBLL1/3/2*WBLL1	MEXICO
TX227	T.DICOCCON PI225332/AE.SQUARROSA (895)//WBLL1/3/2*WBLL1	MEXICO
TX228	T.DICOCCON PI94625/AE.SQUARROSA (372)//3*PASTOR	MEXICO
TX229	T.DICOCCON PI94625/AE.SQUARROSA (372)//3*PASTOR	MEXICO
TX230	T.DICOCCON PI225332/AE.SQUARROSA (895)//WBLL1/3/2*WBLL1	MEXICO
TX231	CHEN/AE.SQ/19/2*BJY/18/KT/6/H/4/3*HRC/FIFE//IM/3/HRC/FIFE//KRYMKI/5/FA/7/H/4/3*HRC/FI<br>FE//IM/3/HRC/FIFE//KRYMKI/5/FA/6/KT/8/KT/5/FRTR/4/RT//SPIJK/SHD/3/AKA/6/H/4/3*HRC/FIF<br>E//IM/3/HRC/FIFE//KRYMKI/5/FA/9/SWD/T.TIMOPHEEVII//RED EGYPTIAN(PI-170925)/KENYA<br>BF4-3B.10.V.1/4/GB/KENIA RF 324/3/BTA/CHI//AMO25E/PELON<br>33C/16/LR64/15/FRTR/4/RT//SPIJK/SHD/3/AKA/5/RED EGYPTIAN(PI-170925)/KENYA BF4-<br>3B.10.V.1/6/H/4/3*HRC/FIFE//IM/3/HRC/FIFE//KRYMKI/13/DRM/MI//KRYMKI/12/KRYMKI/7/FIF<br>E/2*RIBA/6/FIFE/5/FIFE//FIFE/FIFE/4/FIFE/3/FIFE/FIFE//INDIAN G/8/DIEHL/MI/3/PP-<br>AUS//FIFE/ETAWAH/9/ORO/10/DIEHL/MI/3/PP-<br>AUS//FIFE/ETAWAH/11/ORO/8/KRYMKI/7/FIFE/2*RIBA/6/FIFE/5/FIFE//FIFE/FIFE/4/FIFE/3/FIFE/<br>FIFE//INDIAN G/14/SWD/T.TIMOPHEEVII//RED EGYPTIAN(PI-170925)/KENYA BF4-<br>3B.10.V.1/4/GB/KENIA RF	MEXICO
TX232	CHEN/AE.SQ/19/2*BJY/18/KT/6/H/4/3*HRC/FIFE//IM/3/HRC/FIFE//KRYMKI/5/FA/7/H/4/3*HRC/FI<br>FE//IM/3/HRC/FIFE//KRYMKI/5/FA/6/KT/8/KT/5/FRTR/4/RT//SPIJK/SHD/3/AKA/6/H/4/3*HRC/FIF<br>E//IM/3/HRC/FIFE//KRYMKI/5/FA/9/SWD/T.TIMOPHEEVII//RED EGYPTIAN(PI-170925)/KENYA<br>BF4-3B.10.V.1/4/GB/KENIA RF 324/3/BTA/CHI//AMO25E/PELON<br>33C/16/LR64/15/FRTR/4/RT//SPIJK/SHD/3/AKA/5/RED EGYPTIAN(PI-170925)/KENYA BF4-<br>3B.10.V.1/6/H/4/3*HRC/FIFE//IM/3/HRC/FIFEI//KRYMKI/12/KRYMKI/7/FIFE/2*RIBA/6/FIFE/5/FIF<br>E//FIFE/FIFE/4/FIFE/3/FIFE/FIFE//INDIAN G/8/DIEH	MEXICO
TX233	CHEN/AE.SQ/19/2*BJY/18/KT/6/H/4/3*HRC/FIFE//IM/3/HRC/FIFE//KRYMKI/5/FA/7/H/4/3*HRC/FI<br>FE//IM/3/HRC/FIFE//KRYMKI/5/FA/6/KT/8/KT/5/FRTR/4/RT//SPIJK/SHD/3/AKA/6/H/4/3*HRC/FIF<br>E//IM/3/HRC/FIFE//KRYMKI/5/FA/9/SWD/T.TIMOPHEEVII//RED EGYPTIAN(PI-170925)/KENYA<br>BF4-3B.10.V.1/4/GB/KENIA RF 324/3/BTA/CHI//AMO25E/PELON	MEXICO

	33C/16/LR64/15/FRTR/4/RT//SPIJK/SHD/3/AKA/5/RED EGYPTIAN(PI-170925)/KENYA BF4-3B.10.V.1/6/H/4/3*HRC/FIFE//IM/3/HRC/FIFE//KRYMKI/13/DRM/MI//KRYMKI/12/KRYMKI/7/FIFE/2*RIBA/6/FIFE/5/FIFE//FIFE/FIFE/4/FIFE/3/FIFE/FIFE//INDIAN G/8/DIEHL/MI/3/PP-AUS//FIFE/ETAWAH/9/ORO/10/DIEHL/MI/3/PP-AUS//FIFE/ETAWAH/11/ORO/8/KRYMKI/7/FIFE/2*RIBA/6/FIFE/5/FIFE//FIFE/FIFE/4/FIFE/3/FIFE/FIFE//INDIAN	
TX234	FRET2/20/CHEN/AE.SQ/19/2*BJY/18/KT/6/H/4/3*HRC/FIFE//IM/3/HRC/FIFE//KRYMKI/5/FA/7/H/4/3*HRC/FIFE//IM/3/HRC/FIFE//KRYMKI/5/FA/6/KT/8/KT/5/FRTR/4/RT//SPIJK/SHD/3/AKA/6/H/4/3*HRC/FIFE//IM/3/HRC/FIFE//KRYMKI/5/FA/9/SWD/T.TIMOPHEEVII//RED EGYPTIAN(PI-170925)/KENYA BF4-3B.10.V.1/4/GB/KENIA RF 324/3/BTA/CHI//AMO25E/PELON 33C/16/LR64/15/FRTR/4/RT//SPIJK/SHD/3/AKA/5/RED EGYPTIAN(PI-170925)/KENYA BF4-3B.10.V.1/6/H/4/3*HRC/FIFE//IM/3/HRC/FIFE//KRYMKI/13/DRM/MI//KRYMKI/12/KRYMKI/7/FIFE/2*RIBA/6/FIFE/5/FIFE//FIFE/FIFE/4/FIFE/3/FIFE/FIFE//INDIAN G/8/DIEHL/MI/3/PP-AUS//FIFE/ETAWAH/9/ORO/10/DIEHL/MI/3/PP-AUS//FIFE/ETAWAH/11/ORO/8/KRYMKI/7/FIFE/2*RIBA/6/FIFE/5/FIFE//FIFE/FIFE/4/FIFE/3/FIFE/FIFE//INDIAN G/14/SWD/T.TIMOPHEEVII//RED EGYPTIAN(PI-170925)/KENYA BF4-3B.10.V.1/4/GB/KENIA RF 324/3/BTA/CHI//AMO25E/PELON 33C/17/LR64/15/H/4/3*HRC/FIFE//IM/3/HRC/FIFE//KRYMKI/5/FA/6/KT/7/FRTR/4/RT//SPIJK/SHD/3/AKA/13/DRM/MI//KRYMKI/12/KRYMKI/7/FIFE/2*RIBA/6/FIFE/5/FIFE//FIFE/FIFE/4/FIFE/3/FIFE/FIFE//INDIAN G/8/DIEHL/MI/3/PP-AUS//FIFE/ETAWAH/9/ORO/10/DIEHL/MI/3/PP-AUS//FIFE/ETAWAH/11/ORO/8/KRYMKI/7/FIFE/2*RIBA/6/FIFE/5/FIFE//FIFE/FIFE/4/FIFE/3/FIFE/FIFE//INDIAN G/14/2*H/4/3*HRC/FIFE//IM/3/HRC/FIFE//KRYMKI/5/FA/6/EGYPT NA101/7/SWD/T.TIMOPHEEVII	MEXICO
TX235	D65152/D6148//R143/3/ENTE/STR/4/AEGILOPS SQUARROSA (TAUS)/5/WEAVER/6/WEAVER/7/2*FRET2	MEXICO
TX236	EITI 6/UNKNOWN,TUN/8/HEITI/STW//RL-1344/CTN/3/NGT/6/2*CVC1/4/HEITI/STW//RL-1344/CTN/3/NGT/5/UNKNOWN,TUN/7/ALB/9/GOO//ALB/CRA/10/AE.SQUARROSA (193)/11/TILHI/12/FRET2	MEXICO
TX237	SCA/AE.SQUARROSA (518)/3/VEE/JUN//KAUZ/4/VEE/JUN//KAUZ	MEXICO
TX238	ARLIN/AE.SQUARROSA (1017)//ATTILA/3/ATTILA*2/M10 (MUTATED C-306)	MEXICO
TX239	ARLIN/AE.SQUARROSA (1017)//PARUS/3/VEE//VEE*2/PRL	MEXICO
TX240	68.111/RUGBY//WARD/3/FGO/4/RABI/5/AE.SQUARROSA (882)/6/ATTILA/21/ATTILA*2/20/KT/OI/6/FRTR/4/RT//SPIJK/SHD/3/AKA/5/U/7/H/4/3*HRC/FIFE//IM/3/HRC/FIFE//KRYMKI/5/FA/6/KT/8/26591-1T-7M-OY-115Y-OM/9/ALDAN/10/VEE/11/VEE/19/BJY/18/KT/6/H/4/3*HRC/FIFE//IM/3/HRC/FIFE//KRYMKI/5/FA/7/H/4/3*HRC/FIFE//IM/3/HRC/FIFE//KRYMKI/5/FA/6/KT/8/KT/5/FRTR/4/RT//SPIJK/SHD/3/AKA/6/H/4/3*HRC/FIFE//IM/3/HRC/FIFE//KRYMKI/5/FA/9/SWD/T.TIMOPHEEVII//RED EGYPTIAN(PI-170925)/KENYA BF4-3B.10.V.1/4/GB/KENIA RF 324/3/BTA/CHI//AMO25E/PELON 33C/16/LR64/15/FRTR/4/RT//SPIJK/SHD/3/AKA/5/RED EGYPTIAN(PI-170925)/KENYA BF4-3B.10.V.1/6/H/4/3*HRC/FIFE//IM/3/HRC/FIFE//KRYMKI/13/DRM/MI//KRYMKI/12/KRYMKI/7/FIFE/2*RIBA/6/FIFE/5/FIFE//FIFE/FIFE/4/FIFE/3/FIFE/FIFE//INDIAN G/8/DIEHL/MI/3/PP-AUS//FIFE/ETAWAH/9/ORO/10/DIEHL/MI/3/PP-AUS//FIFE/ETAWAH/11/ORO/8/KRYMKI/7/FIFE/2*RIBA/6/FIFE/5/FIFE//FIFE/FIFE/4/FIFE/3/FIFE/FIFE//INDIAN G/14/SWD/T.TIMOPHEEVII//RED EGYPTIAN(PI-170925)/KENYA BF4-3B.10.V.1/4/GB/KENIA RF 324/3/BTA/CHI//AMO25E/PELON 33C/17/LR64/15/H/4/3*HRC/FIFE//IM/3/HRC/FIFE//KRYMKI/5/FA/6/KT/7/FRTR/4/RT//SPIJK/SHD/3/AKA/13/DRM/MI//KRYMKI/12/KRYMKI/7/FIFE/2*RIBA/6/FIFE/5/FIFE//FIFE/FIFE/4/FIFE/3/FIFE/FIFE//INDIAN G/8/DIEHL/MI/3/PP-AUS//FIFE/ETAWAH/9/ORO/10/DIEHL/MI/3/PP-AUS//FIFE/ETAWAH/11/ORO/8/KRYMKI/7/FIFE/2*RIBA/6/FIFE/5/FIFE//FIFE/FIFE/4/FIFE/3/FIFE/FIFE//INDIAN G/14/2*H/4/3*HRC/FIFE//IM/3/HRC/FIFE//KRYMKI/5/FA/6/EGYPT NA101/7/SWD/T.TIMOPHEEVII	MEXICO
TX241	68.111/RUGBY//WARD/3/FGO/4/RABI/5/AE.SQUARROSA (882)/6/ATTILA/21/ATTILA*2/20/KT/OI/6/FRTR/4/RT//SPIJK/SHD/3/AKA/5/U/7/H/4/3*HRC/FIFE//IM/3/HRC/FIFE//KRYMKI/5/FA/6/KT/8/26591-1T-7M-OY-115Y-OM/9/ALDAN/10/VEE/11/VEE/19/BJY/18/KT/6/H/4/3*HRC/FIFE//IM/3/HRC/FIFE//KRYMKI/5/FA/7/H/4/3*HRC/FIFE//IM/3/HRC/FIFE//KRYMKI/5/FA/6/KT/8/KT/5/FRTR/4/RT//SPIJK/SHD/3/AKA/6/H/4/3*HRC/FIFE//IM/3/HRC/FIFE//KRYMKI/5/FA/9/SWD/T.TIMOPHEEVII//RED EGYPTIAN(PI-170925)/KENYA BF4-3B.10.V.1/4/GB/KENIA RF 324/3/BTA/CHI//AMO25E/PELON 33C/16/LR64/15/FRTR/4/RT//SPIJK/SHD/3/AKA/5/RED EGYPTIAN(PI-170925)/KENYA BF4-3B.10.V.1/6/H/4/3*HRC/FIFE//IM/3/HRC/FIFE//KRYMKI/13/DRM/MI//KRYMKI/12/KRYMKI/7/FIFE/2*RIBA/6/FIFE/5/FIFE//FIFE/FIFE/4/FIFE/3/FIFE/FIFE//INDIAN G/8/DIEHL/MI/3/PP-AUS//FIFE/ETAWAH/9/ORO/10/DIEHL/MI/3/PP-AUS//FIFE/ETAWAH/11/ORO/8/KRYMKI/7/FIFE/2*RIBA/6/FIFE/5/FIFE//FIFE/FIFE/4/FIFE/3/FIFE/FIFE//INDIAN G/14/SWD/T.TIMOPHEEVII//RED EGYPTIAN(PI-170925)/KENYA BF4-3B.10.V.1/4/GB/KENIA RF 324/3/BTA/CHI//AMO25E/PELON 33C/17/LR64/15/H/4/3*HRC/FIFE//IM/3/HRC/FIFE//KRYMKI/5/FA/6/KT/7/FRTR/4/RT//SPIJK/SHD/3/AKA/13/DRM/MI//KRYMKI/12/KRYMKI/7/FIFE/2*RIBA/6/FIFE/5/FIFE//FIFE/FIFE/4/FIFE/3/FIFE/FIFE//INDIAN G/8/DIEHL/MI/3/PP-AUS//FIFE/ETAWAH/9/ORO/10/DIEHL/MI/3/PP-AUS//FIFE/ETAWAH/11/ORO/8/KRYMKI/7/FIFE/2*RIBA/6/FIFE/5/FIFE//FIFE/FIFE/4/FIFE/3/FIFE/	MEXICO

	FIFE//INDIAN G/14/2*H/4/3*HRC/FIFE//IM/3/HRC/FIFE//KRYMKI/5/FA/6/EGYPT NA101/7/SWD/T.TIMOPHEEVII	
TX242	FALCIN/AE.SQUARROSA (312)/3/THB/CEP7780//SHA4/LIRA/4/FRET2	MEXICO
TX243	LHNKE/AE.SQUARROSA (224)/19/2*BJY/18/KT/6/H/4/3*HRC/FIFE//IM/3/HRC/FIFE//KRYMKI/5/FA/7/H/4/3*HRC/FIFE//IM/3/HRC/FIFE//KRYMKI/5/FA/6/KT/8/KT/5/FRTR/4/RT//SPIJK/SHD/3/AKA/6/H/4/3*HRC/FIFE//IM/3/HRC/FIFE//KRYMKI/5/FA/9/SWD/T.TIMOPHEEVII//RED EGYPTIAN(PI-170925)/KENYA BF4-3B.10.V.1/4/GB/KENIA RF 324/3/BTA/CHI//AMO25E/PELON 33C/16/LR64/15/FRTR/4/RT//SPIJK/SHD/3/AKA/5/RED EGYPTIAN(PI-170925)/KENYA BF4-3B.10.V.1/6/H/4/3*HRC/FIFE//IM/3/HRC/FIFE//KRYMKI/13/DRM/MI//KRYMKI/12/KRYMKI/7/FIFE/2*RIBA/6/FIFE/5/FIFE//FIFE/FIFE/4/FIFE/3/FIFE/FIFE//INDIAN G/8/DIEHL/MI/3/PP-AUS//FIFE/ETAWAH/9/ORO/10/DIEHL/MI/3/PP-AUS//FIFE/ETAWAH/11/ORO/8/KRYMKI/7/FIFE/2*RIBA/6/FIFE/5/FIFE//FIFE/FIFE/4/FIFE/3/FIFE/FIFE//INDIAN G/14/SWD/T.TIMOPHEEVII//RED EGYPTIAN(PI-170925)/KENYA BF4-3B.10.V.1/4/GB/KENIA RF 324/3/BTA/CHI//AMO25E/PELON 33C/17/LR64/15/H/4/3*HRC/FIFE//IM/3/HRC/FIFE//KRYMKI/5/FA/6/KT/7/FRTR/4/RT//SPIJK/SHD/3/AKA/13/DRM/MI//KRYMKI/12/KRYMKI/7/FIFE/2*RIBA/6/FIFE/5/FIFE//FIFE/FIFE/4/FIFE/3/FIFE/FIFE//INDIAN G/8/DIEHL/MI/3/PP-AUS//FIFE/ETAWAH/9/ORO/10/DIEHL/MI/3/PP-AUS//FIFE/ETAWAH/11/ORO/8/KRYMKI/7/FIFE/2*RIBA/6/FIFE/5/FIFE//FIFE/FIFE/4/FIFE/3/FIFE/FIFE//INDIAN G/14/2*H/4/3*HRC/FIFE//IM/3/HRC/FIFE//KRYMKI/5/FA/6/EGYPT NA101/7/SWD/T.TIMOPHEEVII/20/THB/CEP7780//SHA4/LIRA/21/FRET2	MEXICO
TX244	GA/AE.SQUARROSA (211)/3/HAVIK/KAUZ//KAUZ/4/KASO2	MEXICO
TX245	GA/AE.SQUARROSA (211)/3/HAVIK/KAUZ//KAUZ/4/KASO2	MEXICO
TX246	SCOT/STR//AE.SQUARROSA (314)/21/FRTR/4/RT//SPIJK/SHD/3/AKA/5/HRC/FIFE//IM/3/HRC/FIFE//KRYMKI/4/BTA/AMO44D//BTA/CHI*2/15/H/4/3*HRC/FIFE//IM/3/HRC/FIFE//KRYMKI/5/FA/6/KT/7/FRTR/4/RT//SPIJK/SHD/3/AKA/13/DRM/MI//KRYMKI/12/KRYMKI/7/FIFE/2*RIBA/6/FIFE/5/FIFE//FIFE/FIFE/4/FIFE/3/FIFE/FIFE//INDIAN G/8/DIEHL/MI/3/PP-AUS//FIFE/ETAWAH/9/ORO/10/DIEHL/MI/3/PP-AUS//FIFE/ETAWAH/11/ORO/8/KRYMKI/7/FIFE/2*RIBA/6/FIFE/5/FIFE//FIFE/FIFE/4/FIFE/3/FIFE/FIFE//INDIAN G/14/3*KT/5/FRTR/4/RT//SPIJK/SHD/3/AKA/6/H/4/3*HRC/FIFE//IM/3/HRC/FIFE//KRYMKI/5/FA/16/LR64/15/H/4/3*HRC/FIFE//IM/3/HRC/FIFE//KRYMKI/5/FA/6/KT/7/FRTR/4/RT//SPIJK/SHD/3/AKA/13/DRM/MI//KRYMKI/12/KRYMKI/7/FIFE/2*RIBA/6/FIFE/5/FIFE//FIFE/FIFE/4/FIFE/3/FIFE/FIFE//INDIAN G/8/DIEHL/MI/3/PP-AUS//FIFE/ETAWAH/9/ORO/10/DIEHL/MI/3/PP-AUS//FIFE/ETAWAH/11/ORO/8/KRYMKI/7/FIFE/2*RIBA/6/FIFE/5/FIFE//FIFE/FIFE/4/FIFE/3/FIFE/FIFE//INDIAN G/14/2*H/4/3*HRC/FIFE//IM/3/HRC/FIFE//KRYMKI/5/FA/6/EGYPT NA101/7/SWD/T.TIMOPHEEVII/18/CNO/16/H/4/3*HRC/FIFE//IM/3/HRC/FIFE//KRYMKI/5/FA/6/KT/7/FRTR/4/RT//SPIJK/SHD/3/AKA/13/DRM/MI//KRYMKI/12/KRYMKI/7/FIFE/2*RIBA/6/FIFE/5/FIFE//FIFE/FIFE/4/FIFE/3/FIFE/FIFE//INDIAN G/8/DIEHL/MI/3/PP-AUS//FIFE/ETAWAH/9/ORO/10/DIEHL/MI/3/PP-AUS//FIFE/ETAWAH/11/ORO/8/KRYMKI/7/FIFE/2*RIBA/6/FIFE/5/FIFE//FIFE/FIFE/4/FIFE/3/FIFE/FIFE//INDIAN G/14/2*H/4/3*HRC/FIFE//IM/3/HRC/FIFE//KRYMKI/5/FA/6/EGYPT NA101/7/SWD/T.TIMOPHEEVII/15/FRTR/4/RT//SPIJK/SHD/3/AKA/5/HRC/FIFE//IM/3/HRC/FIFE//KRYMKI/4/BTA/AMO44D//BTA/CHI/6/P4160/17/CRIEWENER 104/CENT.PETKUSER/8/CLTA/5/FRTR/4/RT//SPIJK/SHD/3/AKA/6/UKA/7/KRYMKI//MI/MI/3/KL033/9/BEZ1/19/MN72252/20/SHI4414/CROW/22/METSO	MEXICO
TX247	SCOT/STR//AE.SQUARROSA (314)/21/FRTR/4/RT//SPIJK/SHD/3/AKA/5/HRC/FIFE//IM/3/HRC/FIFE//KRYMKI/4/BTA/AMO44D//BTA/CHI*2/15/H/4/3*HRC/FIFE//IM/3/HRC/FIFE//KRYMKI/5/FA/6/KT/7/FRTR/4/RT//SPIJK/SHD/3/AKA/13/DRM/MI//KRYMKI/12/KRYMKI/7/FIFE/2*RIBA/6/FIFE/5/FIFE//FIFE/FIFE/4/FIFE/3/FIFE/FIFE//INDIAN G/8/DIEHL/MI/3/PP-AUS//FIFE/ETAWAH/9/ORO/10/DIEHL/MI/3/PP-AUS//FIFE/ETAWAH/11/ORO/8/KRYMKI/7/FIFE/2*RIBA/6/FIFE/5/FIFE//FIFE/FIFE/4/FIFE/3/FIFE/FIFE//INDIAN G/14/3*KT/5/FRTR/4/RT//SPIJK/SHD/3/AKA/6/H/4/3*HRC/FIFE//IM/3/HRC/FIFE//KRYMKI/5/FA/16/LR64/15/H/4/3*HRC/FIFE//IM/3/HRC/FIFE//KRYMKI/5/FA/6/KT/7/FRTR/4/RT//SPIJK/SHD/3/AKA/13/DRM/MI//KRYMKI/12/KRYMKI/7/FIFE/2*RIBA/6/FIFE/5/FIFE//FIFE/FIFE/4/FIFE/3/FIFE/FIFE//INDIAN G/8/DIEHL/MI/3/PP-AUS//FIFE/ETAWAH/9/ORO/10/DIEHL/MI/3/PP-AUS//FIFE/ETAWAH/11/ORO/8/KRYMKI/7/FIFE/2*RIBA/6/FIFE/5/FIFE//FIFE/FIFE/4/FIFE/3/FIFE/FIFE//INDIAN G/14/2*H/4/3*HRC/FIFE//IM/3/HRC/FIFE//KRYMKI/5/FA/6/EGYPT NA101/7/SWD/T.TIMOPHEEVII/18/CNO/16/H/4/3*HRC/FIFE//IM/3/HRC/FIFE//KRYMKI/5/FA/6/KT/7/FRTR/4/RT//SPIJK/SHD/3/AKA/13/DRM/MI//KRYMKI/12/KRYMKI/7/FIFE/2*RIBA/6/FIFE/5/FIFE//FIFE/FIFE/4/FIFE/3/FIFE/FIFE//INDIAN G/8/DIEHL/MI/3/PP-AUS//FIFE/ETAWAH/9/ORO/10/DIEHL/MI/3/PP-AUS//FIFE/ETAWAH/11/ORO/8/KRYMKI/7/FIFE/2*RIBA/6/FIFE/5/FIFE//FIFE/FIFE/4/FIFE/3/FIFE/FIFE//INDIAN G/14/2*H/4/3*HRC/FIFE//IM/3/HRC/FIFE//KRYMKI/5/FA/6/EGYPT NA101/7/SWD/T.TIMOPHEEVII/15/FRTR/4/RT//SPIJK/SHD/3/AKA/5/HRC/FIFE//IM/3/HRC/FIFE//KRYMKI/4/BTA/AMO44D//BTA/CHI/6/P4160/17/CRIEWENER 104/CENT.PETKUSER/8/CLTA/5/FRTR/4/RT//SPIJK/SHD/3/AKA/6/UKA/7/KRYMKI//MI/MI/3/KL033/9/BEZ1/19/MN72252/20/SHI4414/CROW/22/METSO	MEXICO

TX248	D67.2/PARANA 66.270//AE.SQUARROSA<br>(220)/23/PRL/21/T.AEST/19/TP/17/CNO/16/LR64/15/H/4/3*HRC/FIFE//IM/3/HRC/FIFE//KRYMKI/5/<br>FA/6/KT/7/FRTR/4/RT//SPIJK/SHD/3/AKA/13/DRM/MI//KRYMKI/12/KRYMKI/7/FIFE/2*RIBA/6/FI<br>FE/5/FIFE//FIFE/FIFE/4/FIFE/3/FIFE/FIFE//INDIAN G/8/DIEHL/MI/3/PP-<br>AUS//FIFE/ETAWAH/9/ORO/10/DIEHL/MI/3/PP-<br>AUS//FIFE/ETAWAH/11/ORO/8/KRYMKI/7/FIFE/2*RIBA/6/FIFE/5/FIFE//FIFE/FIFE/4/FIFE/3/FIFE/<br>FIFE//INDIAN G/14/2*H/4/3*HRC/FIFE//IM/3/HRC/FIFE//KRYMKI/5/FA/6/EGYPT<br>NA101/7/SWD/T.TIMOPHEEVII/18/CNO/15/FRTR/4/RT//SPIJK/SHD/3/AKA/5/RED EGYPTIAN(PI-<br>170925)/KENYA BF4-<br>3B.10.V.1/6/H/4/3*HRC/FIFE//IM/3/HRC/FIFE//KRYMKI/13/DRM/MI//KRYMKI/12/KRYMKI/7/FIF<br>E/2*RIBA/6/FIFE/5/FIFE//FIFE/FIFE/4/FIFE/3/FIFE/FIFE//INDIAN G/8/DIEHL/MI/3/PP-<br>AUS//FIFE/ETAWAH/9/ORO/10/DIEHL/MI/3/PP-<br>AUS//FIFE/ETAWAH/11/ORO/8/KRYMKI/7/FIFE/2*RIBA/6/FIFE/5/FIFE//FIFE/FIFE/4/FIFE/3/FIFE/<br>FIFE//INDIAN G/14/SWD/T.TIMOPHEEVII//RED EGYPTIAN(PI-170925)/KENYA BF4-<br>3B.10.V.1/4/GB/KENIA RF 324/3/BTA/CHI//AMO25E/PELON<br>33C/20/KT/6/H/4/3*HRC/FIFE//IM/3/HRC/FIFE//KRYMKI/5/FA/7/H/4/3*HRC/FIFE//IM/3/HRC/FIFE<br>//KRYMKI/5/FA/6/KT/8/KT/5/FRTR/4/RT//SPIJK/SHD/3/AKA/6/H/4/3*HRC/FIFE//IM/3/HRC/FIFE//<br>KRYMKI/5/FA/9/SWD/T.TIMOPHEEVII//RED EGYPTIAN(PI-170925)/KENYA BF4-<br>3B.10.V.1/4/GB/KENIA RF 324/3/BTA/CHI//AMO25E/PELON<br>33C/16/LR64/15/FRTR/4/RT//SPIJK/SHD/3/AKA/5/RED EGYPTIAN(PI-170925)/KENYA BF4-<br>3B.10.V.1/6/H/4/3*HRC/FIFE//IM/3/HRC/FIFE//KRYMKI/13/DRM/MI//KRYMKI/12/KRYMKI/7/FIF<br>E/2*RIBA/6/FIFE/5/FIFE//FIFE/FIFE/4/FIFE/3/FIFE/FIFE//INDIAN G/8/DIEHL/MI/3/PP-<br>AUS//FIFE/ETAWAH/9/ORO/10/DIEHL/MI/3/PP-<br>AUS//FIFE/ETAWAH/11/ORO/8/KRYMKI/7/FIFE/2*RIBA/6/FIFE/5/FIFE//FIFE/FIFE/4/FIFE/3/FIFE/<br>FIFE//INDIAN G/14/SWD/T.TIMOPHEEVII//RED EGYPTIAN(PI-170925)/KENYA BF4-<br>3B.10.V.1/4/GB/KENIA RF 324/3/BTA/CHI//AMO25E/PELON<br>33C/17/LR64/15/H/4/3*HRC/FIFE//IM/3/HRC/FIFE//KRYMKI/5/FA/6/KT/7/FRTR/4/RT//SPIJK/SHD/<br>3/AKA/13/DRM/MI//KRYMKI/12/KRYMKI/7/FIFE/2*RIBA/6/FIFE/5/FIFE//FIFE/FIFE/4/FIFE/3/FIF<br>E/FIFE//INDIAN G/8/DIEHL/MI/3/PP-AUS//FIFE/ETAWAH/9/ORO/10/DIEHL/MI/3/PP-<br>AUS//FIFE/ETAWAH/11/ORO/8/KRYMKI/7/FIFE/2*RIBA/6/FIFE/5/FIFE//FIFE/FIFE/4/FIFE/3/FIFE/<br>FIFE//INDIAN G/14/2*H/4/3*HRC/FIFE//IM/3/HRC/FIFE//KRYMKI/5/FA/6/EGYPT<br>NA101/7/SWD/T.TIMOPHEEVII/22/TTM/VEE/24/METSO	MEXICO
TX249	CETA/AE.SQUARROSA (1027)/3/VEE/JUN//KAUZ/4/VEE/JUN//KAUZ	MEXICO
TX250	NING CHUN<br>20/3/MYNA/VUL//JUN/7/FILIN/IRENA/6/D65152/D6148//R143/3/ENTE/STR/4/AEGILOPS<br>SQUARROSA (TAUS)/5/WEAVER	MEXICO
TX251	NING CHUN<br>20/3/MYNA/VUL//JUN/7/FILIN/IRENA/6/D65152/D6148//R143/3/ENTE/STR/4/AEGILOPS<br>SQUARROSA (TAUS)/5/WEAVER	MEXICO
TX252	NORM.2-A<br>BC178/20/CHEN/AE.SQ/19/2*BJY/18/KT/6/H/4/3*HRC/FIFE//IM/3/HRC/FIFE//KRYMKI/5/FA/7/H/4<br>/3*HRC/FIFE//IM/3/HRC/FIFE//KRYMKI/5/FA/6/KT/8/KT/5/FRTR/4/RT//SPIJK/SHD/3/AKA/6/H/4/3<br>*HRC/FIFE//IM/3/HRC/FIFE//KRYMKI/5/FA/9/SWD/T.TIMOPHEEVII//RED EGYPTIAN(PI-<br>170925)/KENYA BF4-3B.10.V.1/4/GB/KENIA RF 324/3/BTA/CHI//AMO25E/PELON<br>33C/16/LR64/15/FRTR/4/RT//SPIJK/SHD/3/AKA/5/RED EGYPTIAN(PI-170925)/KENYA BF4-<br>3B.10.V.1/6/H/4/3*HRC/FIFE//IM/3/HRC/FIFE//KRYMKI/13/DRM/MI//KRYMKI/12/KRYMKI/7/FIF<br>E/2*RIBA/6/FIFE/5/FIFE//FIFE/FIFE/4/FIFE/3/FIFE/FIFE//INDIAN G/8/DIEHL/MI/3/PP-<br>AUS//FIFE/ETAWAH/9/ORO/10/DIEHL/MI/3/PP-<br>AUS//FIFE/ETAWAH/11/ORO/8/KRYMKI/7/FIFE/2*RIBA/6/FIFE/5/FIFE//FIFE/FIFE/4/FIFE/3/FIFE/<br>FIFE//INDIAN G/14/SWD/T.TIMOPHEEVII//RED EGYPTIAN(PI-170925)/KENYA BF4-<br>3B.10.V.1/4/GB/KENIA RF 324/3/BTA/CHI//AMO25E/PELON<br>33C/17/LR64/15/H/4/3*HRC/FIFE//IM/3/HRC/FIFE//KRYMKI/5/FA/6/KT/7/FRTR/4/RT//SPIJK/SHD/<br>3/AKA/13/DRM/MI//KRYMKI/12/KRYMKI/7/FIFE/2*RIBA/6/FIFE/5/FIFE//FIFE/FIFE/4/FIFE/3/FIF<br>E/FIFE//INDIAN G/8/DIEHL/MI/3/PP-AUS//FIFE/ETAWAH/9/ORO/10/DIEHL/MI/3/PP-<br>AUS//FIFE/ETAWAH/11/ORO/8/KRYMKI/7/FIFE/2*RIBA/6/FIFE/5/FIFE//FIFE/FIFE/4/FIFE/3/FIFE/<br>FIFE//INDIAN G/14/2*H/4/3*HRC/FIFE//IM/3/HRC/FIFE//KRYMKI/5/FA/6/EGYPT<br>NA101/7/SWD/T.TIMOPHEEVII	MEXICO
TX253	NORM.2-A<br>BC178/20/CHEN/AE.SQ/19/2*BJY/18/KT/6/H/4/3*HRC/FIFE//IM/3/HRC/FIFE//KRYMKI/5/FA/7/H/4<br>/3*HRC/FIFE//IM/3/HRC/FIFE//KRYMKI/5/FA/6/KT/8/KT/5/FRTR/4/RT//SPIJK/SHD/3/AKA/6/H/4/3<br>*HRC/FIFE//IM/3/HRC/FIFE//KRYMKI/5/FA/9/SWD/T.TIMOPHEEVII//RED EGYPTIAN(PI-<br>170925)/KENYA BF4-3B.10.V.1/4/GB/KENIA RF 324/3/BTA/CHI//AMO25E/PELON<br>33C/16/LR64/15/FRTR/4/RT//SPIJK/SHD/3/AKA/5/RED EGYPTIAN(PI-170925)/KENYA BF4-<br>3B.10.V.1/6/H/4/3*HRC/FIFE//IM/3/HRC/FIFE//KRYMKI/13/DRM/MI//KRYMKI/12/KRYMKI/7/FIF<br>E/2*RIBA/6/FIFE/5/FIFE//FIFE/FIFE/4/FIFE/3/FIFE/FIFE//INDIAN G/8/DIEHL/MI/3/PP-<br>AUS//FIFE/ETAWAH/9/ORO/10/DIEHL/MI/3/PP-<br>AUS//FIFE/ETAWAH/11/ORO/8/KRYMKI/7/FIFE/2*RIBA/6/FIFE/5/FIFE//FIFE/FIFE/4/FIFE/3/FIFE/<br>FIFE//INDIAN G/14/SWD/T.TIMOPHEEVII//RED EGYPTIAN(PI-170925)/KENYA BF4-<br>3B.10.V.1/4/GB/KENIA RF 324/3/BTA/CHI//AMO25E/PELON<br>33C/17/LR64/15/H/4/3*HRC/FIFE//IM/3/HRC/FIFE//KRYMKI/5/FA/6/KT/7/FRTR/4/RT//SPIJK/SHD/	MEXICO

	3/AKA/13/DRM/MI//KRYMKI/12/KRYMKI/7/FIFE/2*RIBA/6/FIFE/5/FIFE//FIFE/FIFE/4/FIFE/3/FIFE/FIFE//INDIAN G/8/DIEHL/MI/3/PP-AUS//FIFE/ETAWAH/9/ORO/10/DIEHL/MI/3/PP-AUS//FIFE/ETAWAH/11/ORO/8/KRYMKI/7/FIFE/2*RIBA/6/FIFE/5/FIFE//FIFE/FIFE/4/FIFE/3/FIFE/FIFE//INDIAN G/14/2*H/4/3*HRC/FIFE//IM/3/HRC/FIFE//KRYMKI/5/FA/6/EGYPT NA101/7/SWD/T.TIMOPHEEVII	
TX254	JAGGER/20/CHEN/AE.SQ/19/2*BJY/18/KT/6/H/4/3*HRC/FIFE//IM/3/HRC/FIFE//KRYMKI/5/FA/7/H/4/3*HRC/FIFE//IM/3/HRC/FIFE//KRYMKI/5/FA/6/KT/8/KT/5/FRTR/4/RT//SPIJK/SHD/3/AKA/6/H/4/3*HRC/FIFE//IM/3/HRC/FIFE//KRYMKI/5/FA/9/SWD/T.TIMOPHEEVII//RED EGYPTIAN(PI-170925)/KENYA BF4-3B.10.V.1/4/GB/KENIA RF 324/3/BTA/CHI//AMO25E/PELON 33C/16/LR64/15/FRTR/4/RT//SPIJK/SHD/3/AKA/5/RED EGYPTIAN(PI-170925)/KENYA BF4-3B.10.V.1/6/H/4/3*HRC/FIFE//IM/3/HRC/FIFE//KRYMKI/13/DRM/MI//KRYMKI/12/KRYMKI/7/FIFE/2*RIBA/6/FIFE/5/FIFE//FIFE/FIFE/4/FIFE/3/FIFE/FIFE//INDIAN G/8/DIEHL/MI/3/PP-AUS//FIFE/ETAWAH/9/ORO/10/DIEHL/MI/3/PP-AUS//FIFE/ETAWAH/11/ORO/8/KRYMKI/7/FIFE/2*RIBA/6/FIFE/5/FIFE//FIFE/FIFE/4/FIFE/3/FIFE/FIFE//INDIAN G/14/SWD/T.TIMOPHEEVII//RED EGYPTIAN(PI-170925)/KENYA BF4-3B.10.V.1/4/GB/KENIA RF 324/3/BTA/CHI//AMO25E/PELON 33C/17/LR64/15/H/4/3*HRC/FIFE//IM/3/HRC/FIFE//KRYMKI/5/FA/6/KT/7/FRTR/4/RT//SPIJK/SHD/3/AKA/13/DRM/MI//KRYMKI/12/KRYMKI/7/FIFE/2*RIBA/6/FIFE/5/FIFE//FIFE/FIFE/4/FIFE/3/FIFE/FIFE//INDIAN G/8/DIEHL/MI/3/PP-AUS//FIFE/ETAWAH/9/ORO/10/DIEHL/MI/3/PP-AUS//FIFE/ETAWAH/11/ORO/8/KRYMKI/7/FIFE/2*RIBA/6/FIFE/5/FIFE//FIFE/FIFE/4/FIFE/3/FIFE/FIFE//INDIAN G/14/2*H/4/3*HRC/FIFE//IM/3/HRC/FIFE//KRYMKI/5/FA/6/EGYPT NA101/7/SWD/T.TIMOPHEEVII	MEXICO
TX255	JAGGER/20/CHEN/AE.SQ/19/2*BJY/18/KT/6/H/4/3*HRC/FIFE//IM/3/HRC/FIFE//KRYMKI/5/FA/7/H/4/3*HRC/FIFE//IM/3/HRC/FIFE//KRYMKI/5/FA/6/KT/8/KT/5/FRTR/4/RT//SPIJK/SHD/3/AKA/6/H/4/3*HRC/FIFE//IM/3/HRC/FIFE//KRYMKI/5/FA/9/SWD/T.TIMOPHEEVII//RED EGYPTIAN(PI-170925)/KENYA BF4-3B.10.V.1/4/GB/KENIA RF 324/3/BTA/CHI//AMO25E/PELON 33C/16/LR64/15/FRTR/4/RT//SPIJK/SHD/3/AKA/5/RED EGYPTIAN(PI-170925)/KENYA BF4-3B.10.V.1/6/H/4/3*HRC/FIFE//IM/3/HRC/FIFE//KRYMKI/13/DRM/MI//KRYMKI/12/KRYMKI/7/FIFE/2*RIBA/6/FIFE/5/FIFE//FIFE/FIFE/4/FIFE/3/FIFE/FIFE//INDIAN G/8/DIEHL/MI/3/PP-AUS//FIFE/ETAWAH/9/ORO/10/DIEHL/MI/3/PP-AUS//FIFE/ETAWAH/11/ORO/8/KRYMKI/7/FIFE/2*RIBA/6/FIFE/5/FIFE//FIFE/FIFE/4/FIFE/3/FIFE/FIFE//INDIAN G/14/SWD/T.TIMOPHEEVII//RED EGYPTIAN(PI-170925)/KENYA BF4-3B.10.V.1/4/GB/KENIA RF 324/3/BTA/CHI//AMO25E/PELON 33C/17/LR64/15/H/4/3*HRC/FIFE//IM/3/HRC/FIFE//KRYMKI/5/FA/6/KT/7/FRTR/4/RT//SPIJK/SHD/3/AKA/13/DRM/MI//KRYMKI/12/KRYMKI/7/FIFE/2*RIBA/6/FIFE/5/FIFE//FIFE/FIFE/4/FIFE/3/FIFE/FIFE//INDIAN G/8/DIEHL/MI/3/PP-AUS//FIFE/ETAWAH/9/ORO/10/DIEHL/MI/3/PP-AUS//FIFE/ETAWAH/11/ORO/8/KRYMKI/7/FIFE/2*RIBA/6/FIFE/5/FIFE//FIFE/FIFE/4/FIFE/3/FIFE/FIFE//INDIAN G/14/2*H/4/3*HRC/FIFE//IM/3/HRC/FIFE//KRYMKI/5/FA/6/EGYPT NA101/7/SWD/T.TIMOPHEEVII	MEXICO
TX256	KINCI/20/CHEN/AE.SQ/19/2*BJY/18/KT/6/H/4/3*HRC/FIFE//IM/3/HRC/FIFE//KRYMKI/5/FA/7/H/4/3*HRC/FIFE//IM/3/HRC/FIFE//KRYMKI/5/FA/6/KT/8/KT/5/FRTR/4/RT//SPIJK/SHD/3/AKA/6/H/4/3*HRC/FIFE//IM/3/HRC/FIFE//KRYMKI/5/FA/9/SWD/T.TIMOPHEEVII//RED EGYPTIAN(PI-170925)/KENYA BF4-3B.10.V.1/4/GB/KENIA RF 324/3/BTA/CHI//AMO25E/PELON 33C/16/LR64/15/FRTR/4/RT//SPIJK/SHD/3/AKA/5/RED EGYPTIAN(PI-170925)/KENYA BF4-3B.10.V.1/6/H/4/3*HRC/FIFE//IM/3/HRC/FIFE//KRYMKI/13/DRM/MI//KRYMKI/12/KRYMKI/7/FIFE/2*RIBA/6/FIFE/5/FIFE//FIFE/FIFE/4/FIFE/3/FIFE/FIFE//INDIAN G/8/DIEHL/MI/3/PP-AUS//FIFE/ETAWAH/9/ORO/10/DIEHL/MI/3/PP-AUS//FIFE/ETAWAH/11/ORO/8/KRYMKI/7/FIFE/2*RIBA/6/FIFE/5/FIFE//FIFE/FIFE/4/FIFE/3/FIFE/FIFE//INDIAN G/14/SWD/T.TIMOPHEEVII//RED EGYPTIAN(PI-170925)/KENYA BF4-3B.10.V.1/4/GB/KENIA RF 324/3/BTA/CHI//AMO25E/PELON 33C/17/LR64/15/H/4/3*HRC/FIFE//IM/3/HRC/FIFE//KRYMKI/5/FA/6/KT/7/FRTR/4/RT//SPIJK/SHD/3/AKA/13/DRM/MI//KRYMKI/12/KRYMKI/7/FIFE/2*RIBA/6/FIFE/5/FIFE//FIFE/FIFE/4/FIFE/3/FIFE/FIFE//INDIAN G/8/DIEHL/MI/3/PP-AUS//FIFE/ETAWAH/9/ORO/10/DIEHL/MI/3/PP-AUS//FIFE/ETAWAH/11/ORO/8/KRYMKI/7/FIFE/2*RIBA/6/FIFE/5/FIFE//FIFE/FIFE/4/FIFE/3/FIFE/FIFE//INDIAN G/14/2*H/4/3*HRC/FIFE//IM/3/HRC/FIFE//KRYMKI/5/FA/6/EGYPT NA101/7/SWD/T.TIMOPHEEVII	MEXICO
TX257	NL90.15.2.3	MEXICO
TX258	NL90.15.2.11	MEXICO
TX259	NL90.15.2.44	MEXICO
TX260	NL90.15.2.57	MEXICO
TX261	PBL94.14.39	MEXICO
TX262	QRO94.2.117	MEXICO
TX263	HGO94.8.59	MEXICO
TX264	HGO94.9.1.3	MEXICO
TX265	HGO94.9.1.23	MEXICO
TX266	HGO94.9.1.37	MEXICO
TX267	HGO94.9.2.10	MEXICO
TX268	HGO94.9.2.29	MEXICO
TX269	MEX94.2.39	MEXICO

TX270	MEX94.15.47	MEXICO
TX271	MEX94.22.97	MEXICO
TX272	CHIH95.2.58	MEXICO
TX273	CHIH95.4.3	MEXICO
TX274	CHIH95.8.20	MEXICO
TX275	OAX93.10.1	MEXICO
TX276	OAX93.10.1	MEXICO
TX277	OAX93.10.1	MEXICO
TX278	OAX93.10.1	MEXICO
TX279	PF70354/BOW	MEXICO
TX280	BELEM	PORTUGAL
TX281	HRC/FIFE//IM/3/HRC/FIFE//KRYMKI*6/4/GAZA/2*BOBIN//BUTTON/KENYA 73D2I1C/7/HRC/FIFE//KOTA/3/K39788/5/HRC/FIFE//IM/3/HRC/FIFE//KRYMKI/4/HRC/FIFE//KOTA/6/CDT-U/8/S615.11	MEXICO
TX282	CASTILLA-AG	
TX283	AEGES	
TX284	ELVAS 60P17	PORTUGAL
TX285	ELVAS 61-37	PORTUGAL
TX286	G67786	GREECE
TX287	HRC/FIFE//IM/3/HRC/FIFE//KRYMKI/6/RL-2265/5/3*HRC/FIFE/3/HRC/FIFE//GEHUN/FRASER/4/HRC/FIFE//KRYMKI*2/7/OI/8/CB100	
TX288	HYBRIDE 56 VILMORIN	FRANCE
TX289	KIMMO	
TX290	KIURU	FINLAND
TX291	M-708//G25/NURSI 163	ISRAEL
TX292	CONTO MARZOTTO/9/EUREKA-(=BARLETTA COMUN)/CItr 12362//2*GB/5/RT//SPIJK/SHD/3/AKA/4/6*GB/8/FIFE//VNSS/INDIAN G*2/3/CRETAN/4/KAMBOURICO/5/CLEVARD/SANDS//KENYA C6042/3/BOBIN/GAZA/7/GB/6/GB*2/3/BASIL/JONATHAN//C112364/5/RT//SPIJK/SHD/3/AKA/4/6*GB	
TX293	MISKAAGANI	LEBANON
TX294	MOCHO DE ESPIGA BLANCA	PORTUGAL
TX295	MONDEGO	
TX296	MOYSTAD	
TX297	ODESSA EXP STA 17725	GREECE
TX298	PANE-2	
TX299	H/4/3*HRC/FIFE//IM/3/HRC/FIFE//KRYMKI/5/FA/6/KT/7/FRTR/4/RT//SPIJK/SHD/3/AKA/13/DRM/MI//KRYMKI/12/KRYMKI/7/FIFE/2*RIBA/6/FIFE/5/FIFE//FIFE/FIFE/4/FIFE/3/FIFE/FIFE//INDIAN G/8/DIEHL/MI/3/PP-AUS//FIFE/ETAWAH/9/ORO/10/DIEHL/MI/3/PP-AUS//FIFE/ETAWAH/11/ORO/8/KRYMKI/7/FIFE/2*RIBA/6/FIFE/5/FIFE//FIFE/FIFE/4/FIFE/3/FIFE/FIFE//INDIAN G	
TX300	DGC/9/DGC/8/KRYMKI/7/FIFE/2*RIBA/6/FIFE/5/FIFE//FIFE/FIFE/4/FIFE/3/FIFE/FIFE//INDIAN G	
TX301	ROLLO	
TX302	HRC/FIFE//GEHUN/FRASER/3/PIKA/4/HRC/FIFE//GEHUN/FRASER/3/PIKA/5/LD/FIFE//RIGA M	FINLAND
TX303	SEVILLANO	
TX304	STANLEY(CAN)	
TX305	WEIBULLS 8388/WEIBULLS-8244	
TX306	GA/AE.TA	MEXICO
TX307	KARN/3/FIFE/LD//PERAGIS/SAUMUR DE MARS	
TX308	RT//SPIJK/SHD/3/AKA*3/4/KENIA RF 324/8/H/4/3*HRC/FIFE//IM/3/HRC/FIFE//KRYMKI/5/FA/6/2*ME/7/SUPREZA//H/MI/13/DRM/MI//KRYMKI/12/KRYMKI/7/FIFE/2*RIBA/6/FIFE/5/FIFE//FIFE/FIFE/4/FIFE/3/FIFE/FIFE//INDIAN G/8/DIEHL/MI/3/PP-AUS//FIFE/ETAWAH/9/ORO/10/DIEHL/MI/3/PP-AUS//FIFE/ETAWAH/11/ORO/8/KRYMKI/7/FIFE/2*RIBA/6/FIFE/5/FIFE//FIFE/FIFE/4/FIFE/3/FIFE/FIFE//INDIAN G/15/3*H/4/3*HRC/FIFE//IM/3/HRC/FIFE//KRYMKI/5/FA/6/KT/7/FRTR/4/RT//SPIJK/SHD/3/AKA/13/DRM/MI//KRYMKI/12/KRYM	
TX309	PSSU/5/FRTR/4/RT//SPIJK/SHD/3/AKA	BRAZIL
TX310	BOBIN*2/GAZA	AUSTRALIA
TX311	H/4/3*HRC/FIFE//IM/3/HRC/FIFE//KRYMKI/5/FA/6/KT/7/FRTR/4/RT//SPIJK/SHD/3/AKA/13/DRM/MI//KRYMKI/12/KRYMKI/7/FIFE/2*RIBA/6/FIFE/5/FIFE//FIFE/FIFE/4/FIFE/3/FIFE/FIFE//INDIAN G/8/DIEHL/MI/3/PP-AUS//FIFE/ETAWAH/9/ORO/10/DIEHL/MI/3/PP-AUS//FIFE/ETAWAH/11/ORO/8/KRYMKI/7/FIFE/2*RIBA/6/FIFE/5/FIFE//FIFE/FIFE/4/FIFE/3/FIFE/FIFE//INDIAN G/14/2*H/4/3*HRC/FIFE//IM/3/HRC/FIFE//KRYMKI/5/FA/6/EGYPT NA101/7/SWD/T.TIMOPHEEVII/15/KLH147/KL033/4/BTA/AMO44D//BTA/CHI/3/KL033	MEXICO
TX312	FRTR/4/RT//SPIJK/SHD/3/AKA/5/HRC/FIFE//IM/3/HRC/FIFE//KRYMKI/4/BTA/AMO44D//BTA/CHI/6/PL/15/FRTR/4/RT//SPIJK/SHD/3/AKA/5/RED EGYPTIAN(PI-170925)/KENYA BF4-3B.10.V.1/6/H/4/3*HRC/FIFE//IM/3/HRC/FIFE//KRYMKI/13/DRM/MI//KRYMKI/12/KRYMKI/7/FIF	MEXICO

	E/2*RIBA/6/FIFE/5/FIFE//FIFE/FIFE/4/FIFE/3/FIFE/FIFE//INDIAN G/8/DIEHL/MI/3/PP-AUS//FIFE/ETAWAH/9/ORO/10/DIEHL/MI/3/PP-AUS//FIFE/ETAWAH/11/ORO/8/KRYMKI/7/FIFE/2*RIBA/6/FIFE/5/FIFE//FIFE/FIFE/4/FIFE/3/FIFE/FIFE//INDIAN G/14/SWD/T.TIMOPHEEVII//RED EGYPTIAN(PI-170925)/KENYA BF4-3B.10.V.1/4/GB/KENIA RF 324/3/BTA/CHI//AMO25E/PELON 33C	
TX313	HD1220/15/3*FRTR/4/RT//SPIJK/SHD/3/AKA/5/RED EGYPTIAN(PI-170925)/KENYA BF4-3B.10.V.1/6/H/4/3*HRC/FIFE//IM/3/HRC/FIFE//KRYMKI/13/DRM/MI//KRYMKI/12/KRYMKI/7/FIFE/2*RIBA/6/FIFE/5/FIFE//FIFE/FIFE/4/FIFE/3/FIFE/FIFE//INDIAN G/8/DIEHL/MI/3/PP-AUS//FIFE/ETAWAH/9/ORO/10/DIEHL/MI/3/PP-AUS//FIFE/ETAWAH/11/ORO/8/KRYMKI/7/FIFE/2*RIBA/6/FIFE/5/FIFE//FIFE/FIFE/4/FIFE/3/FIFE/FIFE//INDIAN G/14/SWD/T.TIMOPHEEVII//RED EGYPTIAN(PI-170925)/KENYA BF4-3B.10.V.1/4/GB/KENIA RF 324/3/BTA/CHI//AMO25E/PELON 33C/16/BJY	MEXICO
TX314	PFAU/VEE//BOW	MEXICO
TX315	CHIDDAM D´AUTOMNE A EPI R/PRINCE ALBERT/3/NOE//BLE SEIGLE/HICKLING´S PROLIFIC/4/CHIDDAM BLANC/BLE SEIGLE//NOE/BLE SEIGLE/5/HBR-DU/13/KRYMKI/7/FIFE/2*RIBA/6/FIFE/5/FIFE//FIFE/FIFE/4/FIFE/3/FIFE/FIFE//INDIAN G/8/DIEHL/MI/3/PP-AUS//FIFE/ETAWAH/9/ORO/10/DIEHL/MI/3/PP-AUS//FIFE/ETAWAH/11/ORO/8/KRYMKI/7/FIFE/2*RIBA/6/FIFE/5/FIFE//FIFE/FIFE/4/FIFE/3/FIFE/FIFE//INDIAN G/12/DRM/MI//KRYMKI/16/FRTR/4/RT//SPIJK/SHD/3/AKA/5/RED EGYPTIAN(PI-170925)/KENYA BF4-3B.10.V.1/6/H/4/3*HRC/FIFE//IM/3/HRC/FIFE//KRYMKI/13/DRM/MI//KRYMKI/12/KRYMKI/7/FIFE/2*RIBA/6/FIFE/5/FIFE//FIFE/FIFE/4/FIFE/3/FIFE/FIFE//INDIAN G/8/DIEHL/MI/3/PP-AUS	MEXICO
TX316	FRTR/4/RT//SPIJK/SHD/3/AKA/5/RED EGYPTIAN(PI-170925)/KENYA BF4-3B.10.V.1/6/H/4/3*HRC/FIFE//IM/3/HRC/FIFE//KRYMKI/13/DRM/MI//KRYMKI/12/KRYMKI/7/FIFE/2*RIBA/6/FIFE/5/FIFE//FIFE/FIFE/4/FIFE/3/FIFE/FIFE//INDIAN G/8/DIEHL/MI/3/PP-AUS//FIFE/ETAWAH/9/ORO/10/DIEHL/MI/3/PP-AUS//FIFE/ETAWAH/11/ORO/8/KRYMKI/7/FIFE/2*RIBA/6/FIFE/5/FIFE//FIFE/FIFE/4/FIFE/3/FIFE/FIFE//INDIAN G	MEXICO
TX317	FRTR/4/RT//SPIJK/SHD/3/AKA/5/RED EGYPTIAN(PI-170925)/KENYA BF4-3B.10.V.1/6/H/4/3*HRC/FIFE//IM/3/HRC/FIFE//KRYMKI/13/DRM/MI//KRYMKI/12/KRYMKI/7/FIFE/2*RIBA/6/FIFE/5/FIFE//FIFE/FIFE/4/FIFE/3/FIFE/FIFE//INDIAN G/8/DIEHL/MI/3/PP-AUS//FIFE/ETAWAH/9/ORO/10/DIEHL/MI/3/PP-AUS//FIFE/ETAWAH/11/ORO/8/KRYMKI/7/FIFE/2*RIBA/6/FIFE/5/FIFE//FIFE/FIFE/4/FIFE/3/FIFE/FIFE//INDIAN G/14/SWD/T.TIMOPHEEVII//RED EGYPTIAN(PI-170925)/KENYA BF4-3B.10.V.1/4/GB/KENIA RF 324/3/BTA/CHI//AMO25E/PELON 33C	MEXICO
TX318	II53.388/7/KT/5/FRTR/4/RT//SPIJK/SHD/3/AKA/6/H/4/3*HRC/FIFE//IM/3/HRC/FIFE//KRYMKI/5/FA/14/H/4/3*HRC/FIFE//IM/3/HRC/FIFE//KRYMKI/5/FA/6/KT/7/FRTR/4/RT//SPIJK/SHD/3/AKA/13/DRM/MI//KRYMKI/12/KRYMKI/7/FIFE/2*RIBA/6/FIFE/5/FIFE//FIFE/FIFE/4/FIFE/3/FIFE/FIFE//INDIAN G/8/DIEHL/MI/3/PP-AUS//FIFE/ETAWAH/9/ORO/10/DIEHL/MI/3/PP-AUS//FIFE/ETAWAH/11/ORO/8/KRYMKI/7/FIFE/2*RIBA/6/FIFE/5/FIFE//FIFE/FIFE/4/FIFE/3/FIFE/FIFE//INDIAN G/15/RT//SPIJK/SHD/3/AKA*3/4/KENIA RF 324/8/H/4/3*HRC/FIFE//IM/3/HRC/FIFE//KRYMKI/5/FA/6/2*ME/7/SUPREZA//H/MI/16/B4946.A.4.18.2.1Y/7/H/4/3*HRC/FIFE//IM/3/HRC/FIFE//KRYMKI/5/FA/6/EGYPT NA101//SWD/T.TIMOPHEEVII/8/3*H/4/3*HRC/FIFE//IM/3/HRC/FIFE//KRYMKI/5/FA	MEXICO
TX319	H/4/3*HRC/FIFE//IM/3/HRC/FIFE//KRYMKI/5/FA/6/KT/7/FRTR/4/RT//SPIJK/SHD/3/AKA/13/DRM/MI//KRYMKI/12/KRYMKI/7/FIFE/2*RIBA/6/FIFE/5/FIFE//FIFE/FIFE/4/FIFE/3/FIFE/FIFE//INDIAN G/8/DIEHL/MI/3/PP-AUS//FIFE/ETAWAH/9/ORO/10/DIEHL/MI/3/PP-AUS//FIFE/ETAWAH/11/ORO/8/KRYMKI/7/FIFE/2*RIBA/6/FIFE/5/FIFE//FIFE/FIFE/4/FIFE/3/FIFE/FIFE//INDIAN G/14/2*H/4/3*HRC/FIFE//IM/3/HRC/FIFE//KRYMKI/5/FA/6/EGYPT NA101/7/SWD/T.TIMOPHEEVII	MEXICO
TX320	URUGUAY 1084/NX DIRK 48	N/A

Supplementary Table S8. Details of the spring wheat reference set (SWRS) accessions used in the present study.

Gene	Primer Sequence (5'-3')
TraesCS5B02G193100<br>(Trehalose-6-Phosphate)	Forward: TCAAAGTCCAAGGCCAAAGG<br>Reverse: AGCCGTTGATGCACCTCTTC
TraesCS5B02G193200<br>(APETALA2/Ethylene-responsive factor	Forward: GATGCAGCAGCTCGACTTCAG<br>Reverse: TCGATCTCATACGACGGGTACTT
TraesCS5A02G401800<br>(DNA-binding One Zinc Finger; Dof TF	Forward: CCTCCAACACCAAGTTCTGCTACT<br>Reverse: CGGCACGTCTTGCAGAAGT
TraesCS2A02G547600<br>(Gibberellin-dioxygenases; GAox	Forward: CGGTGGAGGAAGGTGATGTC<br>Reverse: AGGCTCCGTTCTCTTCTTGCT
TraesCS1B02G055800<br>(SET)	Forward: GTCCCGCAGGAGGAACTTG<br>Reverse: CGCCACGGACGACATACTG
TaActin	Forward: CCTTGTTTGCGACAATGGAA<br>Reverse: AGCCCTTGGTGCATCATCTC

Supplementary Table S9. Primer sequences of genes utilized in qRT-PCR expression analysis.
